# Supplementary material for: Burdens of non-communicable disease attributable to metabolic risk factors in Australia, 1990–2019: joinpoint regression analysis of the Global Burden of Disease Study
Source: BMJ Open. 2023 Jul 14;13(7):e071319. doi: 10.1136/bmjopen-2022-071319 (PMC10351265; doi:10.1136/bmjopen-2022-071319)
Supplement: Supplementary data [file bmjopen-2022-071319supp001.pdf]

The burdens of non-communicable disease attributable to metabolic risk factors in Australia, 1990–2019: Joinpoint Regression Analysis of the Global Burden of Disease Study

Kelemu Tilahun Kibret[1*], Kathryn Backholer[1] Anna Peeters[1] Fisaha Tesfay[1] Melanie Nichols[1]

[1]Deakin University, Global Centre for Preventive Health and Nutrition, Institute for Health Transformation, School of Health and Social Development, Faculty of Health, 1 Gheringhap Street, Geelong, VIC 3220, Australia.

*Corresponding Author: kelemu.kibret@deakin.edu.au

Supplementary Tables

Supplementary Table 1 Age standardised rates of summary exposure value (SEV) of metabolic risk factors and average annual percentage change, 1990 - 2019

Risk factor	1990 SEV	2010 SEV	2019 SEV	AAPC 1990-2019	AAPC 2010-19
Metabolic risk factors (combined)	24.0 (19.1, 29.7)	30.8 (25.3, 36.7)	33.2 (27.4, 39.3)	1.1 (1.1, 1.1) *	0.8 (0.8,0.9) *
High systolic blood pressure	28.0 (26.1, 30.1)	21.8 (19.8, 23.9)	24.4 (20.7, 28.3)	-0.5 (-0.5, -0.4) *	1.2 (1.1,1.3) *
High body mass index	22.9 (16.7, 31.1)	33.4 (26.1, 41.5)	35.8 (27.9, 44.1)	1.5 (1.5, 1.6) *	0.8 (0.7,0.8) *
Fasting plasma glucose	4.56 (4.1, 5.1)	7.7 (6.7, 8.7)	8.3 (7.2, 9.6)	2.1 (1.6, 2.6) *	0.6 (0.5,1.7) *
High LDL	49.4 (46.5, 52.3)	47.3 (44.2, 50.3)	48.6 (45.5, 51.7)	0.0 (-0.1, 0.1)	0.4 (0.4,0.5) *

AAPC- average annual percentage change, SEV- summary exposure value, * indicates AAPC is significantly different from zero at the alpha = 0.05 level

Supplementary Table 2. Numbers, age standardised rates and proportions of metabolic risk related NCDs death and DALYs by sex in Australia between 1990 and 2019.

	Number (95% UI)				Age standardised Proportion, % (95%UI)				Age standardised rate per 100,000 (95% UI)			
	1990	2010	2019	% Change 1990-2019	1990	2010	2019	% Change 1990-2019	1990	2010	2019	% Change 1990-2019
**Deaths**												
Male	23052 (21126, 24865)	21726 (19333, 24152)	26,399 (23330, 29621)	13.5 (10.4, 9.1)	41.6 (37.9, 5.1)	33.6 (30.0, 37.3)	32.5 (28.8, 36.3)	-20.2 (-23.9, -19.6)	302.0 (274.7, 327.6)	150.0 (133.7,166.7)	133.9 (118.6, 149.9)	-51.3 (-56.9, -54.3)
Female	22523 (20061, 24693)	23767 (20558, 26597)	26,970 (23156, 30516)	19.8 (15.43, 23.58)	43.7 (38.9, 48.0)	34.8 (30.2, 39.0)	32.8 (28.2, 37.2)	-25.0 (-27.5, -22.5)	199.8 (177.6, 219.0)	107.2 (93.2, 120.2)	94.7 (81.6, 107.4)	52.6 (-54.1, -51.0)
Both	45575 (41530, 49465)	45492 (40228, 50512)	53369 (46725, 59687)	17.1 (12.51, 20.67)	43.0 (38.8, 46.8)	34.4 (30.5, 38.2)	32.8 (28.7, 36.6)	-23.8 (-26.0, -21.8)	245.3 (221.7, 266.8)	127.3 (112.6, 141.1)	113.3 (99.5, 126.6)	-53.8 (-55.1, -52.6)
**DALYs**												
Male	524488 (478223, 573378)	482011 (421511, 548065)	571369 (497917, 659110)	8.9 (4.1, 15.0)	26.6 (23.8, 29.6)	19.4 (17.0, 22.1)	18.9 (16.4, 21.5)	-30.0 (-31.0, -27.5)	6124.6 (5580.6, 6704.6)	3,321.8 (2903.8, 3787.0)	3095.8 (2692.8, 3578.5)	-49.5 (-51.8, -46.6)

Female	411233 (366423, 458999)	425811 (362905, 493866)	489350 (411090, 574086)	19.0 (12.2, 25.1)	19.6 (16.8, 22.6)	14.6 (12.28, 16.9)	14.0 (11.8, 16.4)	-28.8 (-29.7, -27.3)	3,764.7 (3346.1, 4212.5)	2,351.2 (1973.1, 2770.7)	2198.7 (1827.9, 2623.3)	-41.6 (-45.4, -37.7)
Both	935,721 (846039, 1031253)	907822 (786590, 1040661)	1,060719 (911391, 1228621)	13.4 (7.7, 19.1)	23.4 (20.6, 26.4)	17.0 (14.7, 19.6)	16.4 (14.15, 19.0)	-29.7 (-31.1, -28.1)	4,870.9 (4402.1, 5377.5)	2818.2 (2439.8, 3246.5)	2,630.3 (2,246.3, 3063.7)	-46.0 (-49.0, -43.0)

Supplementary Table 3. Burden of non-communicable diseases (deaths and DALYs) attributed to metabolic risk factors in Australia, 2019

Risk factors	Deaths (95%UI)			DALYS (95%UI)		
	Number	Proportion, %(95%UI)	ASR (per 100,000)	Number	Proportion, % (95%UI)	ASR (per 100,000)
Metabolic risk factors combined	53369 (46725,59687)	34.4(30.1, 38.5	113.33(99.49- 126.60)	1060720(911391,1228621)	20.0(17.3, 22.8)	2630.33(246.30,3063.65)
HBP	25498 (20153,30851)	16.4 (12.9, 19.8)	53.8 (43.4, 64.4)	406445 (342906, 471339)	7.7 (6.3, 9.1)	966.5 (824.7, 1111.5)
BMI	18713 (11915,26149)	12.0 (7.7, 16.8)	41.7 (27.0, 57.5)	525135 (353676, 706414)	9.9 (6.8, 12.9)	1387.7 (954.66, 1855.0)
FPG	17640 (12073,25501)	11.4 (7.8, 16.4)	37.2 (25.7, 53.1)	381681 (286916, 499517)	7.2 (5.7, 9.1)	919.9 (698.2, 1187.7)
High LDL	14013 (9490,19216)	9.0 (6.1, 12.4)	30.0 (21.1, 40.10)	212393 (163123, 265910)	4.0 (3.0, 5.1)	525.2 (420.9, 639.0)

ASR- age standardised rate; DALY- disability adjusted life years; UI – uncertainty intervals

Supplementary Table 4. Crude numbers, proportions of specific NCD deaths and DALYs attributable to metabolic risks in 1990 and 2019

Specific Causes of NCDs	Deaths attributable to combined metabolic risks n (95% UI)		DALYs attributable to combined metabolic risks n (95% UI)	
	1990	2019	1990	2019
All NCDs	45575 (49465, 41530)	53369 (59688, 46725)	935721 (846039, 1031253)	1060720 (911391, 1228621)
Neoplasms	2220 (1184, 3500)	5644 (3273, 8658)	47916 (26339, 73613)	110091 (65433, 164880)
Cardiovascular diseases	38412 (34749, 41552)	34921 (29085, 39517)	711313 (657997, 759437)	537319 (475508, 595062)
Chronic respiratory diseases	216 (122, 337)	160 (98, 236)	18915 (30673, 10535)	24758 (15038, 38755)
Neurological disorders	729 (118, 2228)	2954 (529, 8488)	11625 (3291, 29053)	41079 (12673, 98146)
Diabetes mellitus	2198 (2060, 2304.)	4239 (3726, 4629)	78885 (66264, 93765)	186528 (142543, 236662)
Chronic kidney disease	1712 (1557, 1827)	5207 (4293, 5893)	36869 (33602, 40498)	86550 (76642, 96199)
	Proportion % (95% UI)		Proportion % (95% UI)	
All NCDs	42.9 (39.0, 46.5)	34.4 (30.1, 38.5)	24.4 (2.3, 27.5)	20.0 (17.4, 22.8)
Neoplasms	7.1 (3.8, 11.0)	10.5 (6.1, 16.0)	6.4 (3.5, 9.8)	10.0 (5.9, 14.9)
Cardiovascular diseases	75.0 (69.5, 80.2)	67.7 (60.7, 73.7)	75.3 (70.7, 79.6)	68.3 (62.8, 73.3)
Chronic respiratory diseases	3.1 (1.8, 4.8)	1.4 (0.9, 2.1)	7.6 (4.4, 11.6)	7.5 (4.8, 11.1)
Neurological disorders	12.4 (4.0, 24.3)	17.5 (6.5, 30.4)	5.3 (1.8, 12.0)	9.8 (3.8, 19.4)
Diabetes mellitus	100.0 (100.0, 100.0)	100 (100.0,100.0)	100 (100.0, 100.0)	100 (100.0,100.0)
Chronic kidney disease	100 (100.0,100.0)	100 (100.0,100.0)	100 (100.0,100.0)	100 (100.0,100.0)

DALY- disability adjusted life years; NCDs – Non-communicable diseases; UI- uncertainty intervals

Supplementary Table 5. Trends in metabolic risk-related death rates of specific NCDs (age standardised rates) in Australia, 1990 – 2019

Specific Causes of NCDs	Age-standardized death rate (95% UI) per 100,000)			AAPC (95% CI)	
	1990	2010	2019	1990-2019	2010-2019
**Metabolic risks combined**					
Neoplasms	11.4 (6.1 18.0)	13.1 (7.6 , 20.0)	13.0 (7.5, 19.9)	0.5 (0.4, 0.6) *	0.0 (-0.0, 0.1)
Cardiovascular diseases	206.8 (185.1, 224.5)	86.2 (74.6, 96.2)	73.6 (62.2, 82.8)	-3.5 (-3.7, -3.3) *	-1.9 (-2.2, -1.5) *
Chronic respiratory diseases	1.1 (0.6, 1.8)	0.4 (0.3, 0.6)	0.4 (0.2, 0.6)	-3.7 (-4.3, -3.1) *	-1.0 (-1.2, -0.8) *
Neurological disorders	4.5 (0.7, 13.3)	5.8 (1.0, 16.7)	5.8 (1.0, 16.6)	1.0 (0.9, 1.0) *	-0.0 (-0.1, 0.0)
Diabetes mellitus	11.6 (10.7, 12.1)	11.1 (9.9, 11.8)	9.2 (8.2, 10.0)	-0.8 (-1.0, -0.5) *	-2.1 (-2.7, -1.4) *
Chronic kidney disease	9.5 (8.6, 10.1)	10.1 (8.7, 11.0)	10.8 (9.03, 12.1)	0.5 (0.2, 0.9) *	0.7 (0.5, 0.9) *
**High body mass index**					
Neoplasms	7.5 (4.2, 11.4)	8.3 (5.1, 11.8)	8.5 (5.2, 12.0)	0.4 (0.3, 0.5) *	0.1 (0.1, 0.2) *
Cardiovascular diseases	45.6 (26.2, 68.1)	22.5 (14.1, 32.0)	20.1 (12.7, 28.4)	-2.8 (-3.0, -2.6) *	-1.4 (-1.7, -1.1) *
Chronic respiratory diseases	2.8 (1.4, 4.5)	3.5 (1.6, 5.5)	0.4 (0.2, 0.6)	-3.7 (-4.3, -3.1) *	-1.0 (-1.2, -0.8) *

Neurological disorders	3.1 (0.5, 9.5)	4.0 (0.7, 11.8)	4.1 (0.7, 12.2)	1.1 (1.0, 1.0) *	0.3 (0.2, 0.5) *
Diabetes mellitus	4.8 (3.1, 6.6)	5.1 (3.4, 6.9)	4.3 (2.9, 5.9)	-0.4 (-0.6, 0.1) *	-2.0 (-2.6, -1.4) *
Chronic kidney disease	2.8 (1.4, 4.5)	3.5 (1.6, 5.5)	3.8 (1.8, 6.0)	1.1 (0.8, 1.4) *	0.9 (0.5, 1.4) *
**High fast plasma glucose**					
Neoplasms	4.1 (1.1, 8.5)	5.1 (1.4, 10.5)	4.8 (1.3, 9.9)	0.6 (0.4, 0.8) *	-0.6 (-1.1, -0.0) *
Cardiovascular diseases	32.4 (19.9, 53.5)	26.0 (14.8, 44.2)	20.1 (11.8, 33.4)	-1.5 (-1.8, -1.2) *	-2.5 (-2.9, -2.1) *
Neurological disorders	1.6 (0.2, 6.3)	2.2 (0.2, 8.4)	2.1 (0.2, 7.8)	0.9 (0.8, 0.9) *	-0.8 (-1.0, -0.7) *
Diabetes mellitus	11.6 (10.7, 12.1)	11.1 (9.9, 11.8)	9.2 (8.2, 10.0)	-0.8 (-1.0, -0.5) *	-2.1 (-2.7, -1.4) *
Chronic kidney disease	0.6 (0.4, 0.8)	1.0 (0.9, 1.2)	1.0 (0.7, 1.5)	2.0 (1.7, 2.2) *	-0.4 (-0.6, -0.1) *
**High Systolic blood pressure**					
Cardiovascular diseases	149.6 (125.5, 170.5	54.7 (44.2, 65.5)	47.3 (37.5, 57.3)	-3.9 (-4.1, -3.7) *	-1.8 (-2.2, -1.5) *
Chronic kidney disease	6.1 (5.2, 6.9)	6.2 (5.2, 7.1)	6.5 (5.3, 7.7)	0.3 (-0.1, 0.6)	0.6 (0.4,0.8)
**High LDL cholesterol**					

Cardiovascular diseases	94.9 (70.4, 122.1	35.5 (24.8, 47.7)	30.0 (21.1, 40.1)	-3.9 (-4.1, -3.7) *	-2.0 (-2.4, -1.6) *

AAPC- Average annual percent change; LDL-low density lipoprotein; NCDs – Non-communicable diseases; UI- uncertainty intervals

*AAPC is significantly different from zero at the α = 0.05 levels

Supplementary Table 6. Trends in metabolic risk-attributed DALY rates of specific NCDs (age standardised rates) in Australia

	Age-standardized DALY rates (95% UI) per 100,000)			AAPC (95% CI)	
Specific Causes of NCDs	1990	2010	2019	1990-2019	2010-2019
**Combined metabolic risks**					
Neoplasms	247.4 (136.6, 379.1)	274.8 (162.7, 410.0)	274.2 (164.4, 407.6)	0.4 (0.3, 0.4) *	-0.0 (-0.1, 0.0)
Cardiovascular diseases	3689.4 (3407.5, 3945.1)	1479.7 (1338.2, 1615.6)	1279.8 (1145.9, 1410.1)	-3.6 (-3.8, -3.4) *	-1.7 (-2.0, -1.4) *
Chronic respiratory diseases	104.0 (58.7, 167.7)	83.7 (50.8, 127.7)	88.52 (54.1, 138.7)	-0.6 (-0.8, -0.4) *	0.9 (0.7, 1.1) *
Neurological disorders	65.0 (18.1, 161.8)	84.6 (26.5, 198.8)	85.1 (26.4, 202.8)	1.0 (0.9, 1.0) *	0.1 (0.0, 0.2) *
Diabetes mellitus	409.5 (343.5, 486.2)	484.9 (386.0, 610.1)	480.8 (365.6, 613.0)	0.6 (0.5, 0.6) *	-0.1 (-0.6, 0.5)
Chronic kidney disease	195.4 (178.0, 214.3)	198.5 (177.7, 220.9)	208.5 (184.8, 233.2)	0.3 (0.0, 0.5) *	0.5 (-0.2, 1.3) *
**High body mass index**					
Neoplasms	173.6 (99.7, 260.5)	187.5 (116.6, 264.1)	190.3 (118.8, 265.9)	0.3 (0.2, 0.4) *	0.1 (0.0, 0.2) *
Cardiovascular diseases	1057.9 (633.0, 1511.9)	532.5 (361.1, 716.4)	477.8 (327.8, 641.5)	-2.7 (-3.0, -2.5) *	-1.2 (-1.5, -0.1) *
Chronic respiratory diseases	104.0 (58.7, 167.7)	83.7 (50.8, 127.7)	88.5 (54.1, 138.7)	-0.6 (-0.8, -0.4) *	0.9 (0.7, 1.1) *

Neurological disorders	46.3 (11.4, 120.2)	59.4 (16.6, 144.5)	61.6 (17.4, 152.5)	1.0 (0.9, 1.0) *	0.4 (0.3, 0.5) *
Diabetes mellitus	198.7 (134.2, 270.9)	270.2 (187.7, 366.0)	279.5 (192.1, 383.4)	1.2 (1.0, 1.5) *	0.5 (0.4, 0.5) *
Chronic kidney disease	64.6 (37.0, 95.1)	76.0 (47.0, 106.9)	82.7 (52.6, 116.2)	0.9 (0.7, 1.1) *	0.9 (0.2, 1.6) *
**High fast plasma glucose**					
Neoplasms	76.9 (19.7, 161.3)	92.2 (24.6, 189.4)	89.1 (23.9, 184.0)	0.6 (0.5, 0.7) *	-0.2 (-0.3, 0.2) *
Cardiovascular diseases	471.5 (314.5, 714.9)	353.4 (218.7, 561.1)	289.8 (186.2, 455.0)	-1.5 (-1.8, -1.3) *	-1.9 (-2.3, -1.5) *
Neurological disorders	21.7 (3.5,74.0)	30.9 (5.2, 101.0)	29.1 (5.1, 95.7)	1.0 (0.9, 1.1) *	-0.7 (-0.8, -0.5) *
Diabetes mellitus	409.5 (343.5, 486.2)	484.9 (386.0, 610.1)	480.8 (365.6, 613.0)	0.6 (0.5, 0.6) *	-0.1 (-0.1, 0.0)
Chronic kidney disease	19.9 (15.9, 24.8)	29.3 (24.8, 35.1)	29.1 (22.1, 37.3)	1.3 (1.3, 1.4) *	0.1 (-0.1, 0.2)
**High Systolic blood pressure**					
Cardiovascular diseases	2713.3 (2402.2, 3033.0)	974.9 (839.3, 1112.3)	855.6 (720.6, 989.9)	-3.9 (-4.1, -3.8) *	-1.6 (-1.9, -1.3) *
Chronic kidney disease	109.3 (92.8, 126.6)	104.7 (88.6, 120.8)	110.9 (92.3, 131.4)	0.1 (-0.0, 0.3)	0.7 (0.6, 0.9) *
**High LDL cholesterol**					
Cardiovascular diseases	1757.9 (1443.6, 2100.7)	623.8 (504.1, 761.0)	525.2 (420.9, 639.0)	-4.1 (-4.4, -3.8) *	-2.0 (-2.3, -1.7) *

AAPC- Average annual percent change; LDL-low density lipoprotein; NCDs – Non-communicable diseases; UI- uncertainty intervals; *AAPC is significantly different from zero at the alpha = 0.05 levels

Supplementary Figures

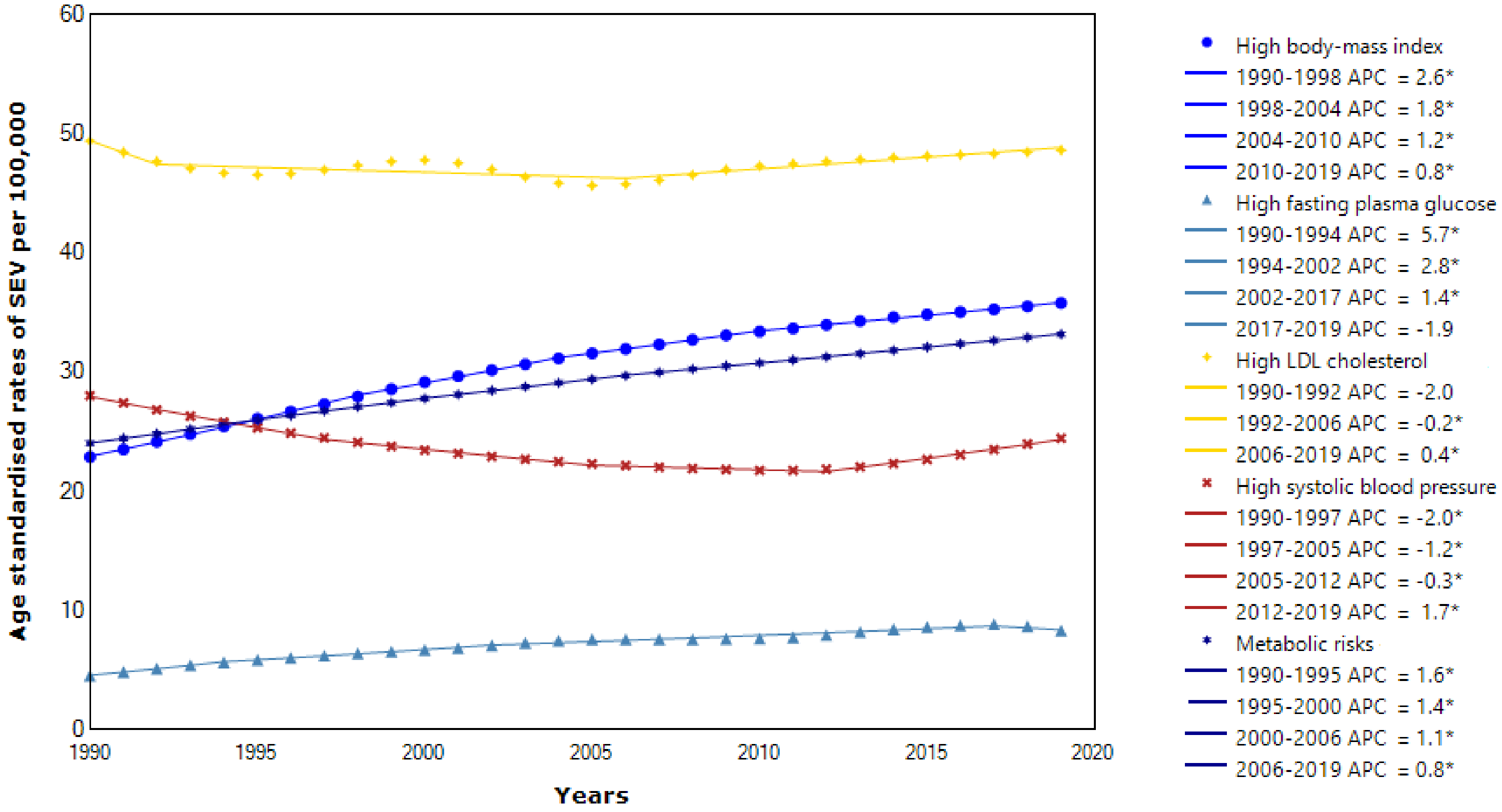

Supplementary Figure 1. Trends of SEV of combined and individual metabolic risks factors between 1990 and 2019, in Australia. Note: * indicates APC is significantly different from zero at the α = 0.05 level

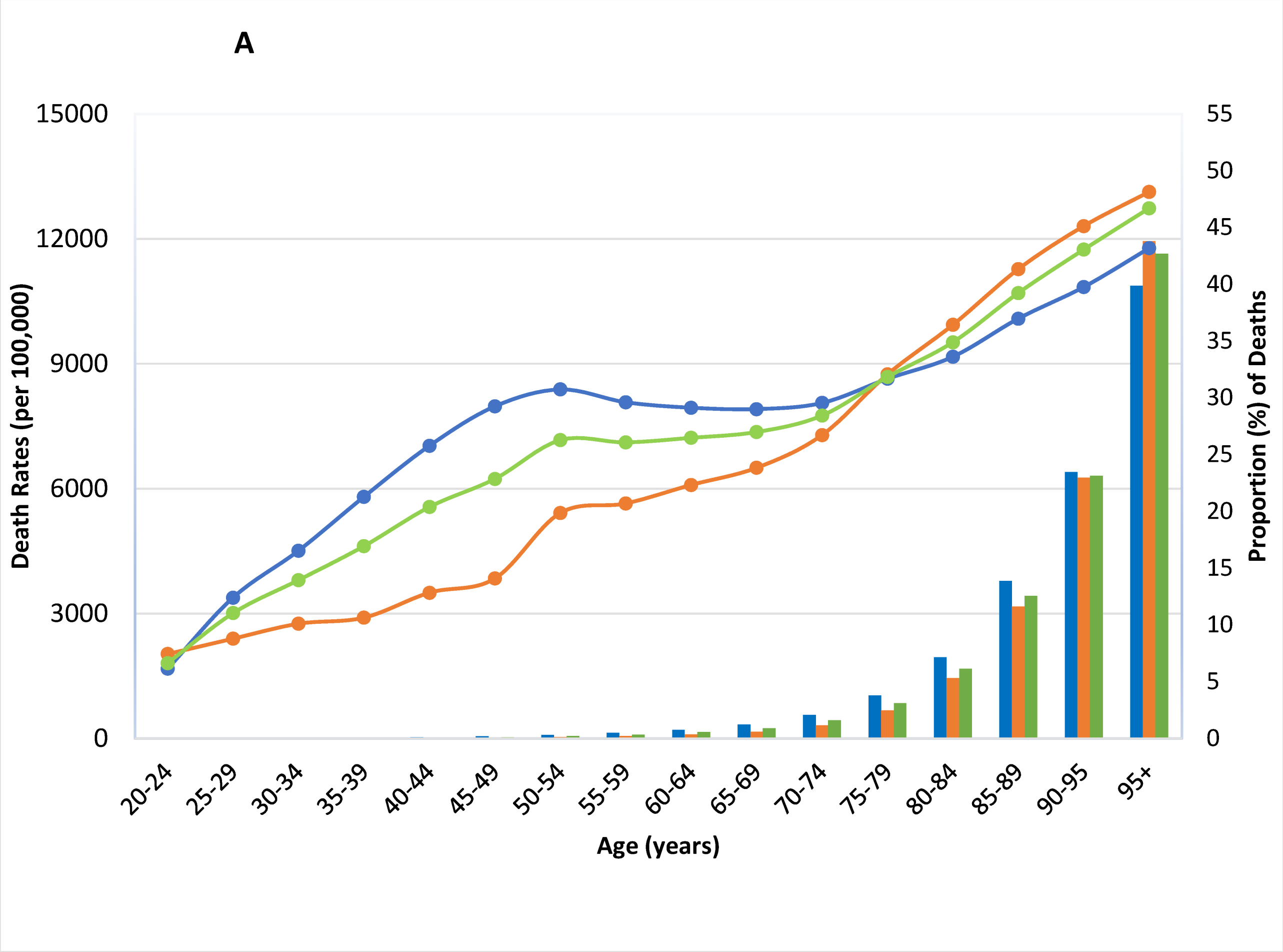
A
Death Rates (per 100,000)
15000
12000
9000
6000
3000
0
Proportion (%) of Deaths
55
50
45
40
35
30
25
20
15
10
5
0
20-24
25-29
30-34
35-39
40-44
45-49
50-54
55-59
60-64
65-69
70-74
75-79
80-84
85-89
90-95
95+
Age (years)

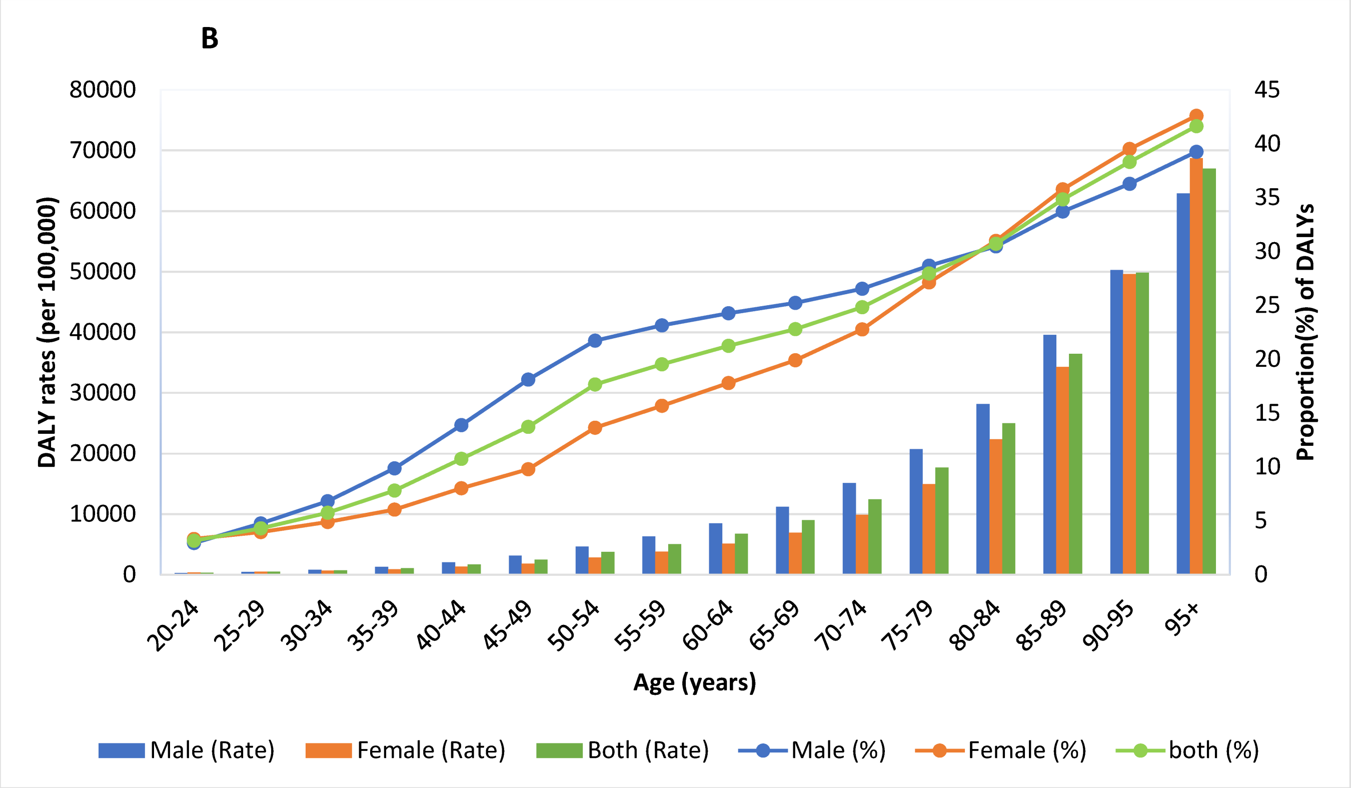

Supplementary Figure 2. Age-specific rates and proportions of non-communicable disease deaths (**A**), and DALYs (**B**) attributable to metabolic risk factors by sex in Australia in 2019 (Proportions are of all NCD deaths/DALYs).

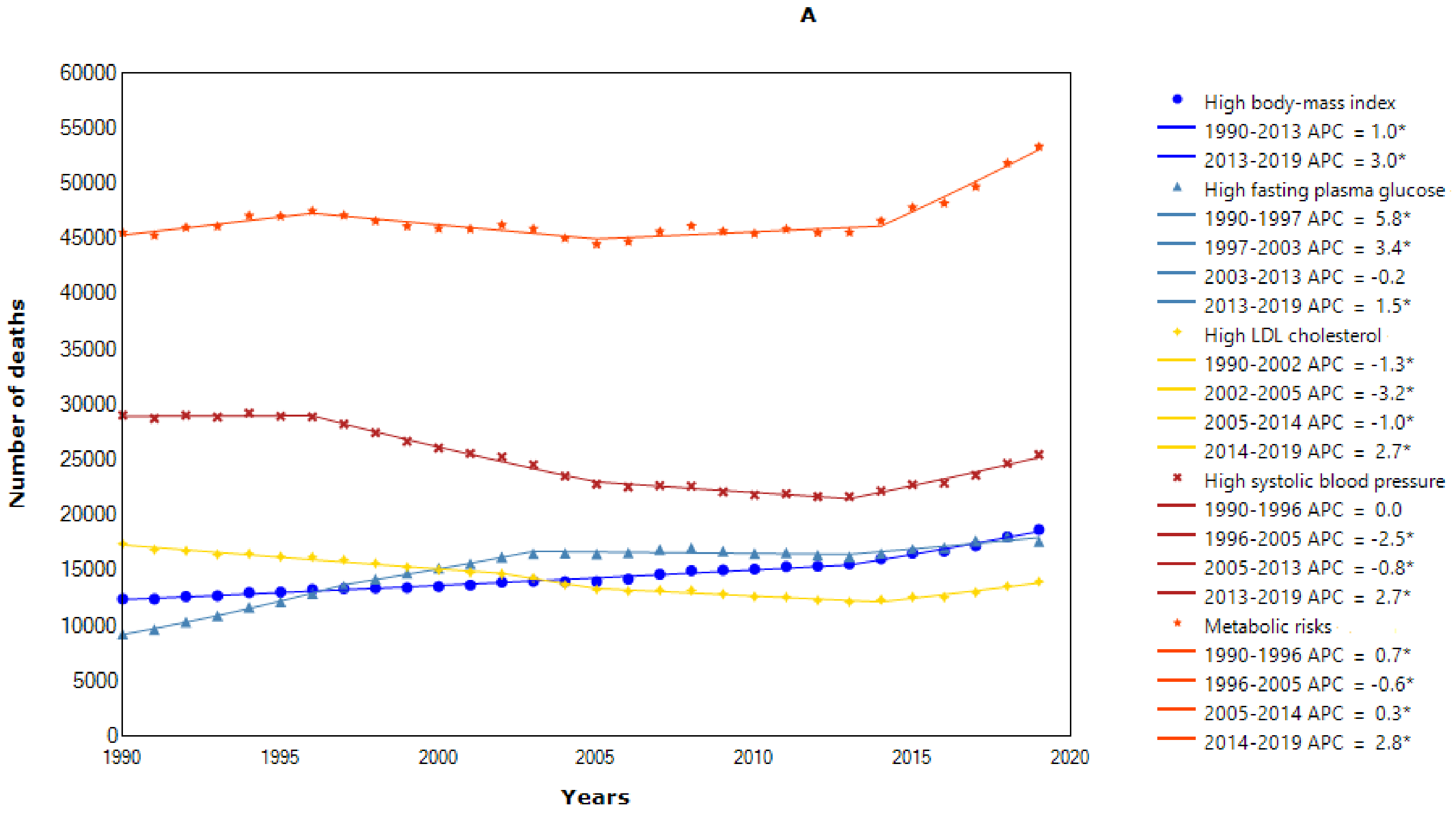
A
Number of deaths
60000
55000
50000
45000
40000
35000
30000
25000
20000
15000
10000
5000
0
1990
1995
2000
2005
2010
2015
2020
Years
High body-mass index
1990-2013 APC = 1.0*
2013-2019 APC = 3.0*
High fasting plasma glucose
1990-1997 APC = 5.8*
1997-2003 APC = 3.4*
2003-2013 APC = -0.2
2013-2019 APC = 1.5*
High LDL cholesterol
1990-2002 APC = -1.3*
2002-2005 APC = -3.2*
2005-2014 APC = -1.0*
2014-2019 APC = 2.7*
High systolic blood pressure
1990-1996 APC = 0.0
1996-2005 APC = -2.5*
2005-2013 APC = -0.8*
2013-2019 APC = 2.7*
Metabolic risks
1990-1996 APC = 0.7*
1996-2005 APC = -0.6*
2005-2014 APC = 0.3*
2014-2019 APC = 2.8*

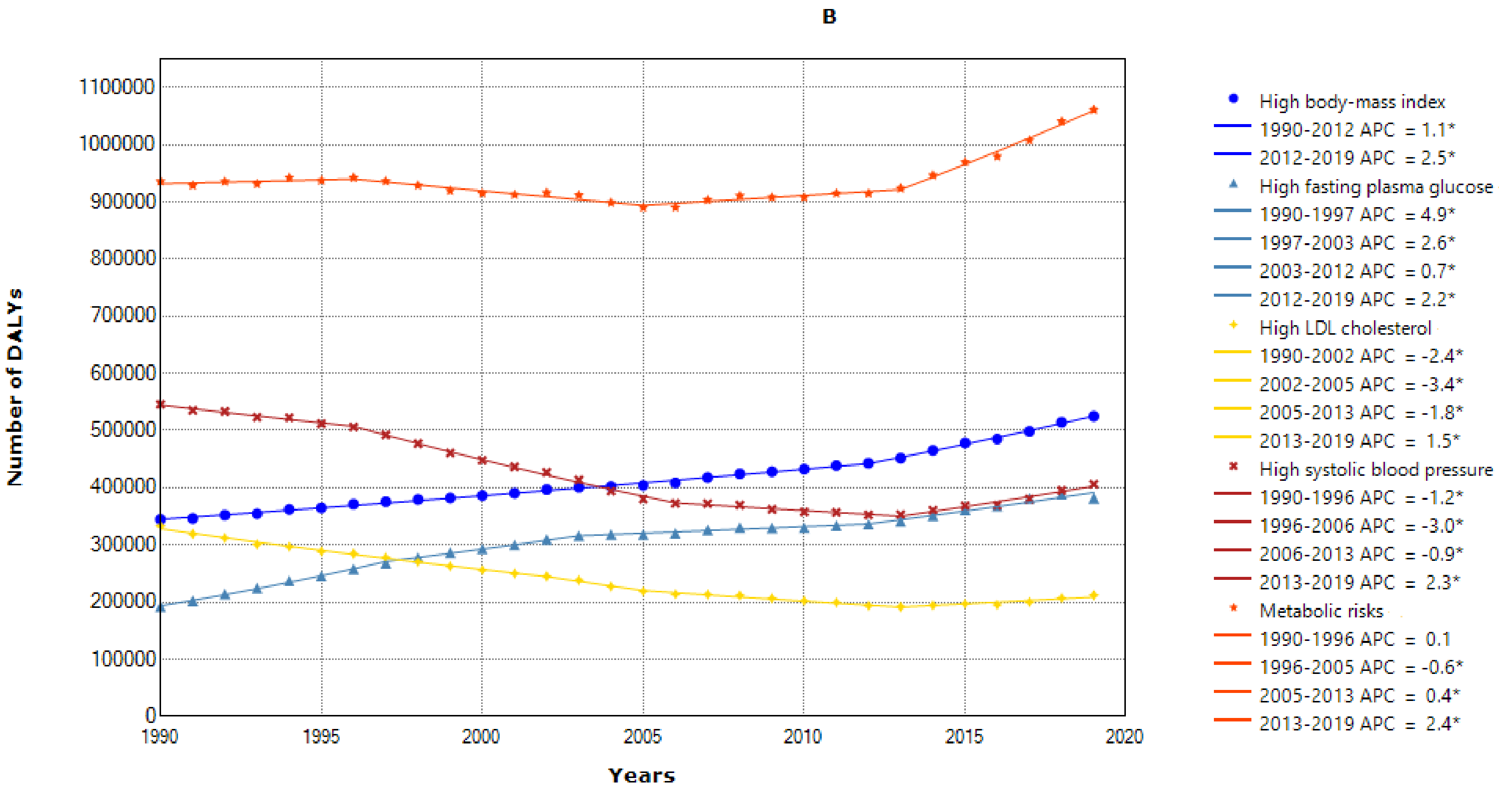

Supplementary Figures 3. Number of deaths (**A**) and DALYs (**B**) attributed to individual and combined metabolic risks overt time (1990-2019) in Australia from Join point regression analysis (Note: * indicates APC is significantly different from zero at the α = 0.05 levels).

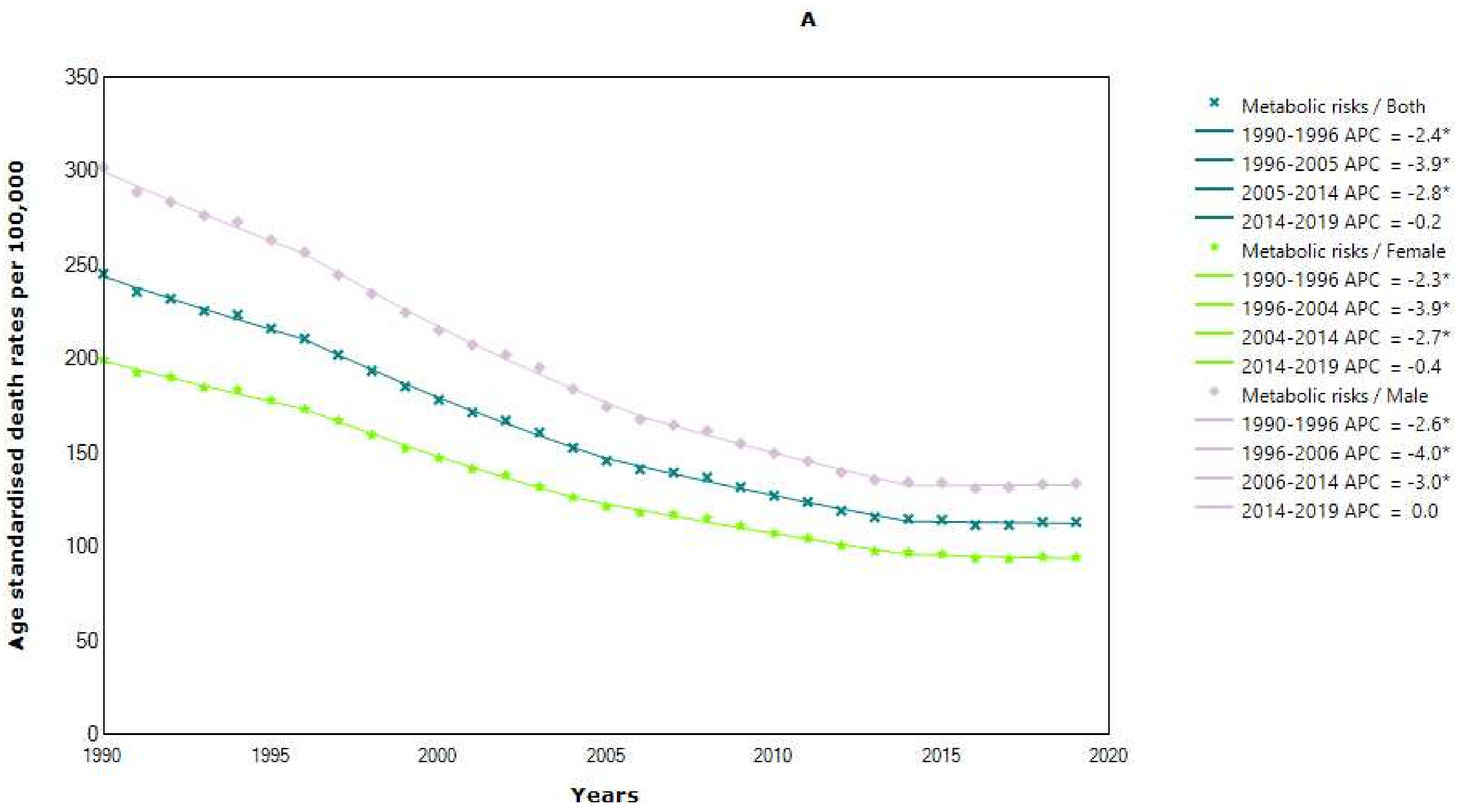
A
Age standardised death rates per 100,000
350
300
250
200
150
100
50
0
1990
1995
2000
2005
2010
2015
2020
Years
Metabolic risks / Both
1990-1996 APC = -2.4*
1996-2005 APC = -3.9*
2005-2014 APC = -2.8*
2014-2019 APC = -0.2
Metabolic risks / Female
1990-1996 APC = -2.3*
1996-2004 APC = -3.9*
2004-2014 APC = -2.7*
2014-2019 APC = -0.4
Metabolic risks / Male
1990-1996 APC = -2.6*
1996-2006 APC = -4.0*
2006-2014 APC = -3.0*
2014-2019 APC = 0.0

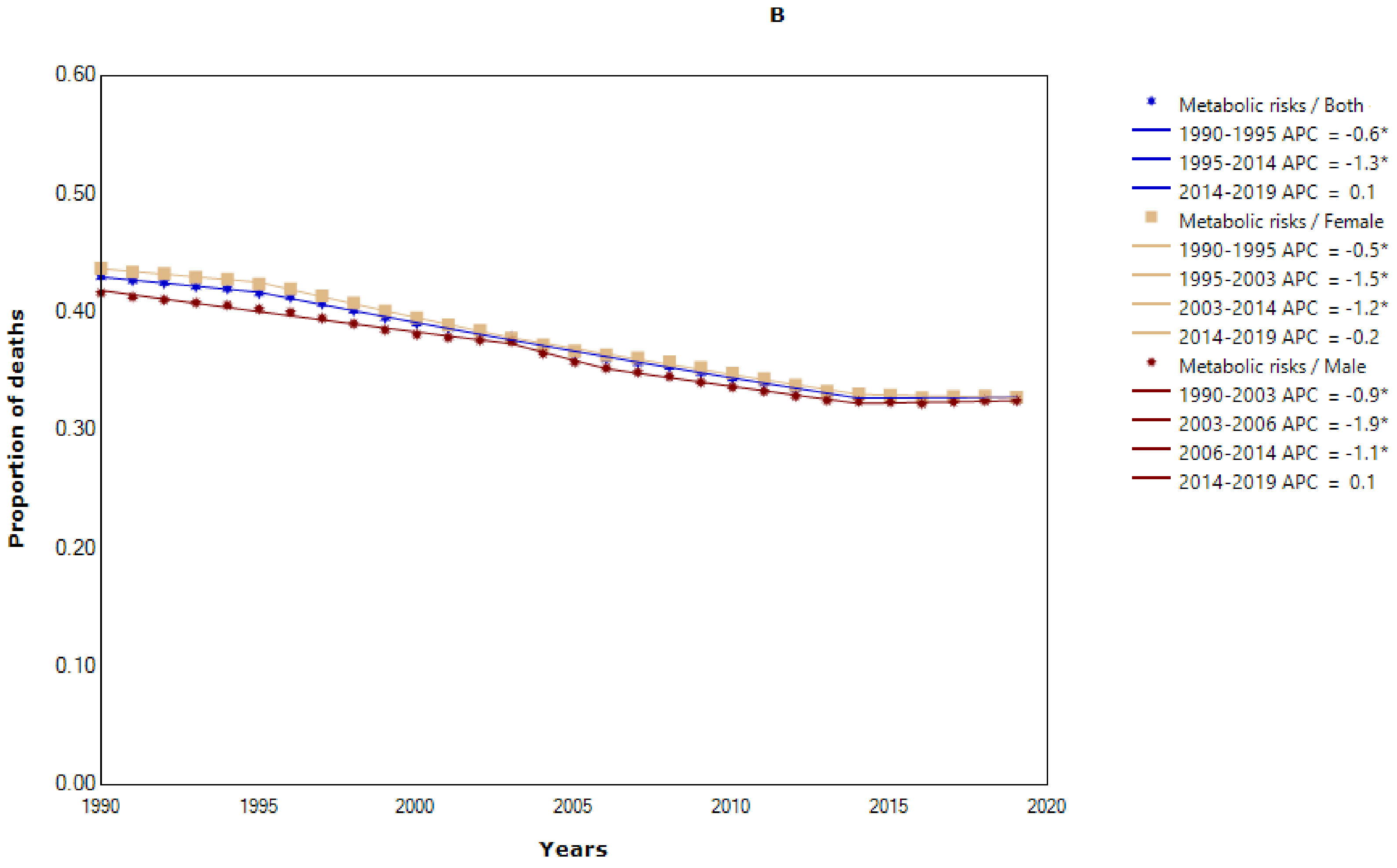
B
Proportion of deaths
0.60
0.50
0.40
0.30
0.20
0.10
0.00
1990
1995
2000
2005
2010
2015
2020
Years
Metabolic risks / Both
1990-1995 APC = -0.6*
1995-2014 APC = -1.3*
2014-2019 APC = 0.1
Metabolic risks / Female
1990-1995 APC = -0.5*
1995-2003 APC = -1.5*
2003-2014 APC = -1.2*
2014-2019 APC = -0.2
Metabolic risks / Male
1990-2003 APC = -0.9*
2003-2006 APC = -1.9*
2006-2014 APC = -1.1*
2014-2019 APC = 0.1

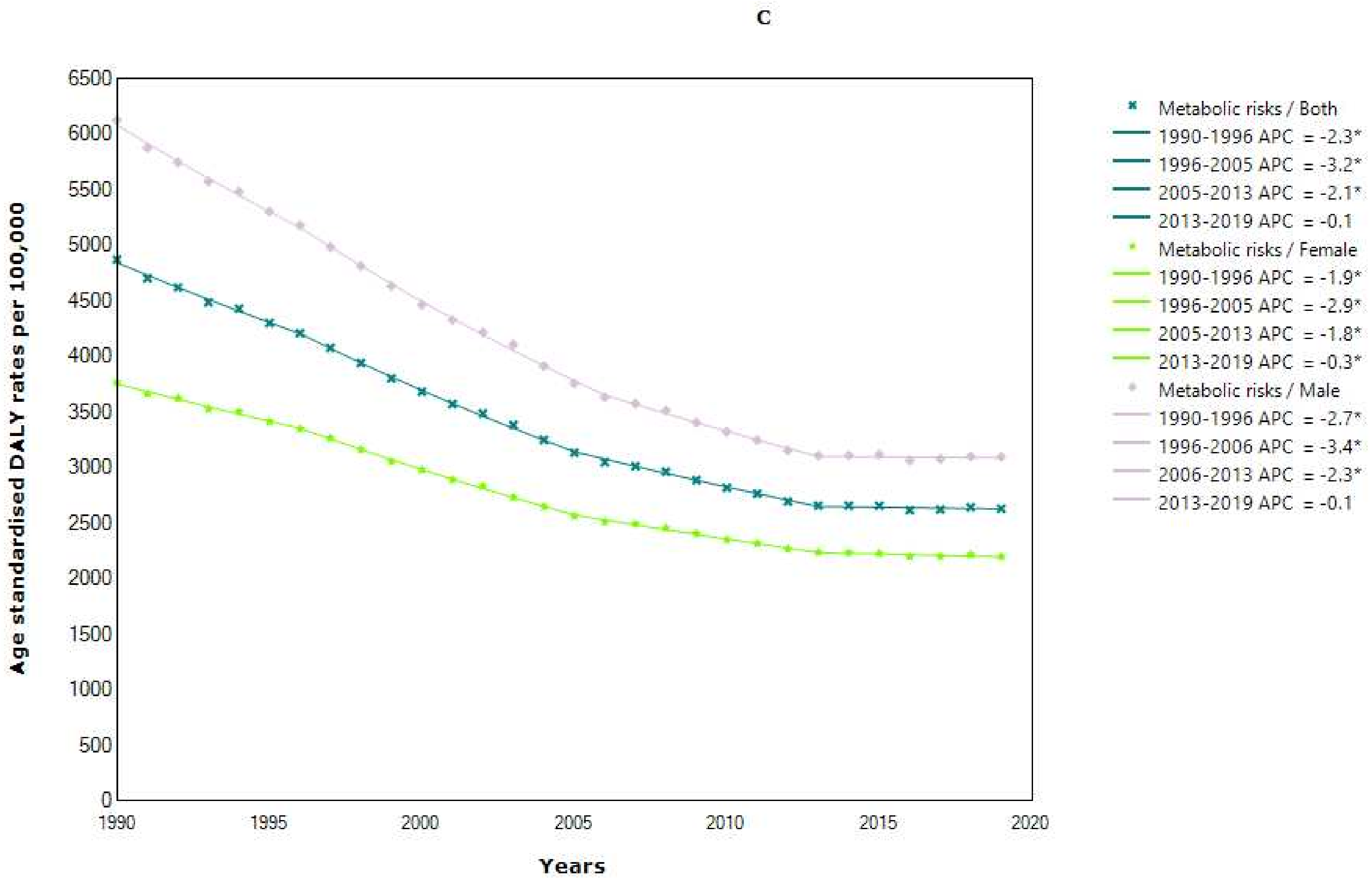
C
Age standardised DALY rates per 100,000
6500
6000
5500
5000
4500
4000
3500
3000
2500
2000
1500
1000
500
0
1990
1995
2000
2005
2010
2015
2020
Years
Metabolic risks / Both
1990-1996 APC = -2.3*
1996-2005 APC = -3.2*
2005-2013 APC = -2.1*
2013-2019 APC = -0.1
Metabolic risks / Female
1990-1996 APC = -1.9*
1996-2005 APC = -2.9*
2005-2013 APC = -1.8*
2013-2019 APC = -0.3*
Metabolic risks / Male
1990-1996 APC = -2.7*
1996-2006 APC = -3.4*
2006-2013 APC = -2.3*
2013-2019 APC = -0.1

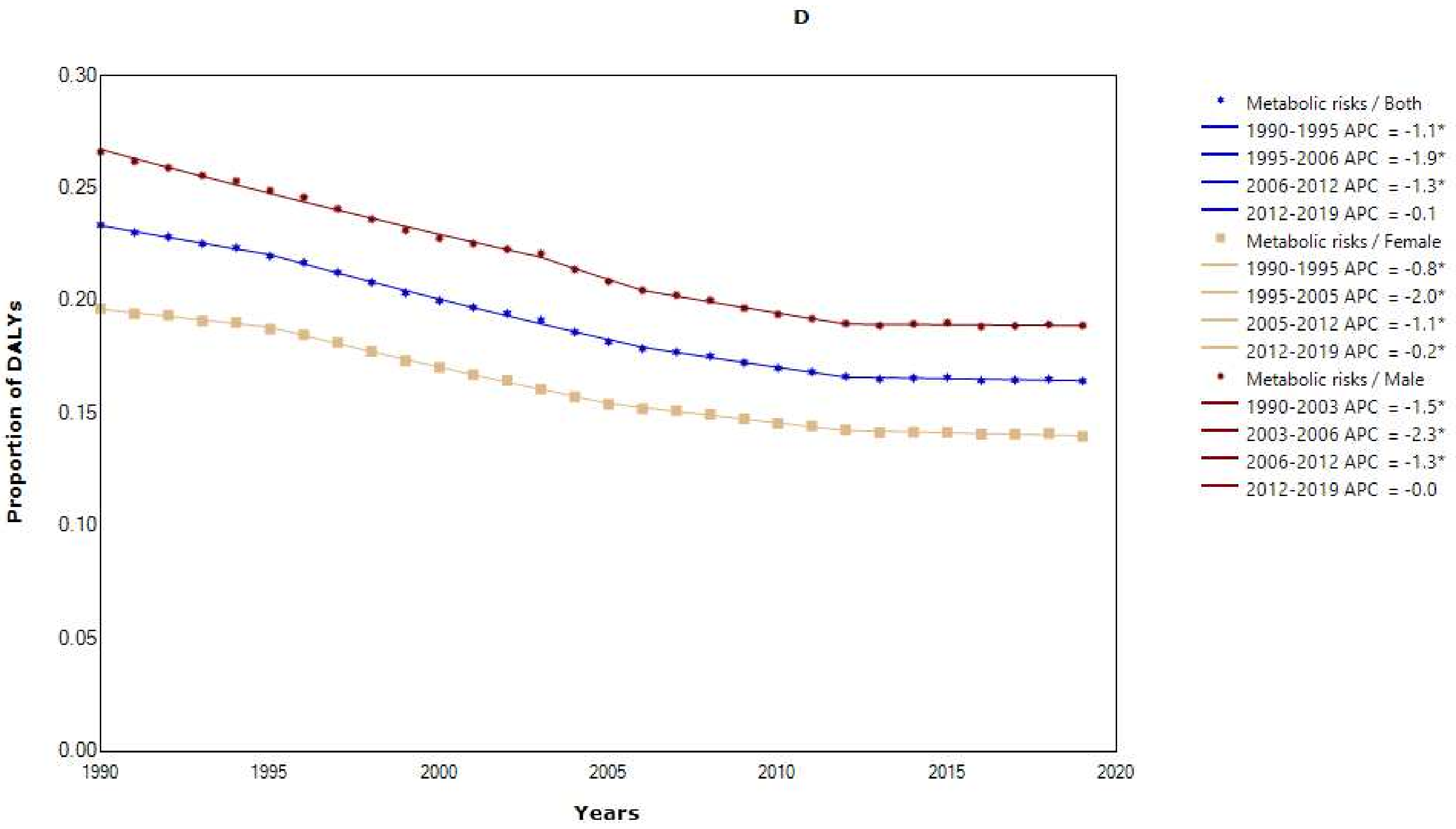

Supplementary Figure 4. Trends of NCDs deaths and DALYs attributable to combined metabolic risks over time (1990 to 2019) by sex in Australia from Join point regression analysis: Age-standardized death rates (**A**); proportions of deaths **(B)**; Age standardised DALY rates **(C)** proportion of DALYs (**D**) (Note: * indicates APC is significantly different from zero at the α = 0.05 levels)

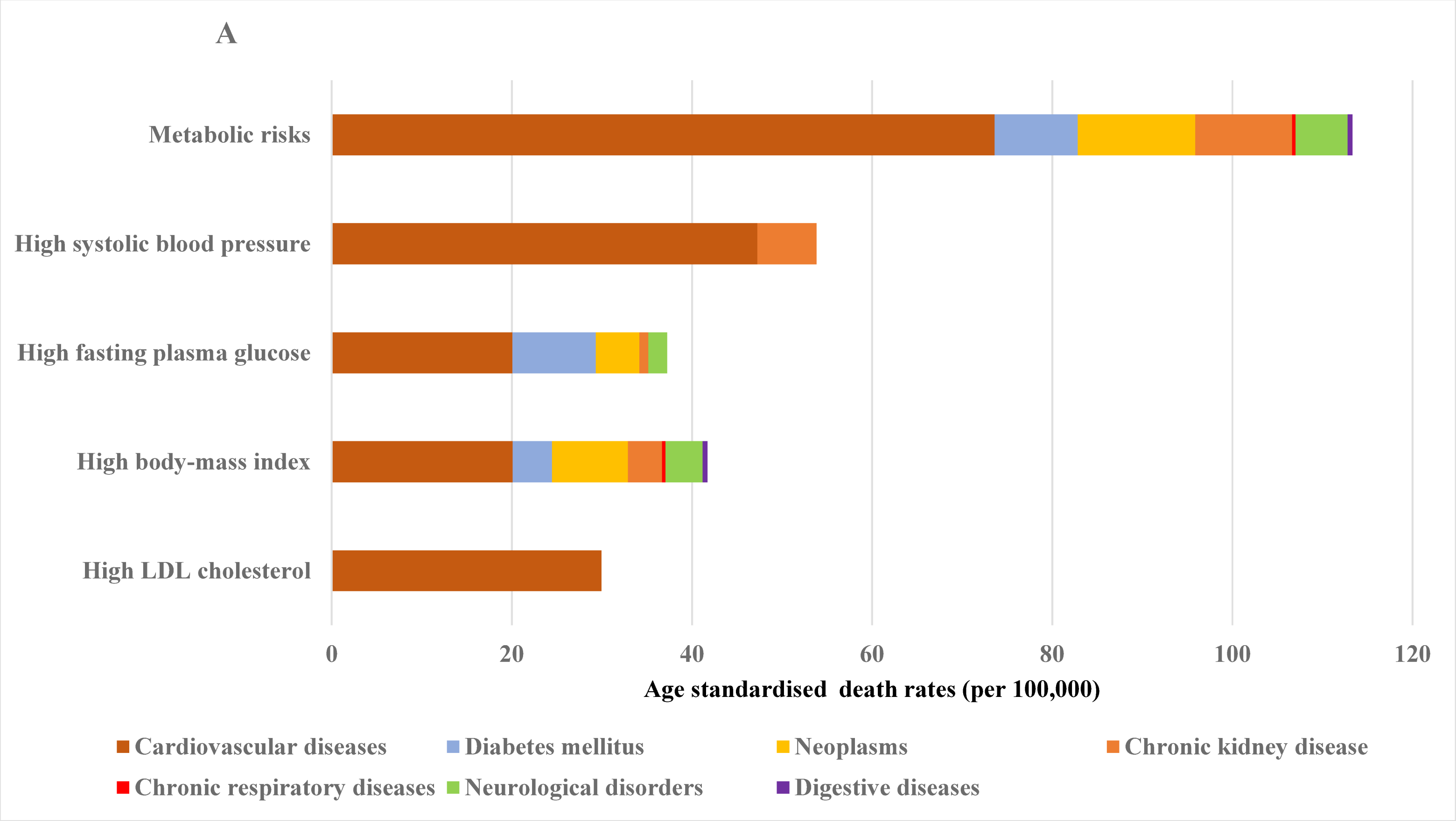
A
Metabolic risks
High systolic blood pressure
High fasting plasma glucose
High body-mass index
High LDL cholesterol
0
20
40
60
80
100
120
Age standardised death rates (per 100,000)
Cardiovascular diseases
Diabetes mellitus
Neoplasms
Chronic kidney disease
Chronic respiratory diseases
Neurological disorders
Digestive diseases

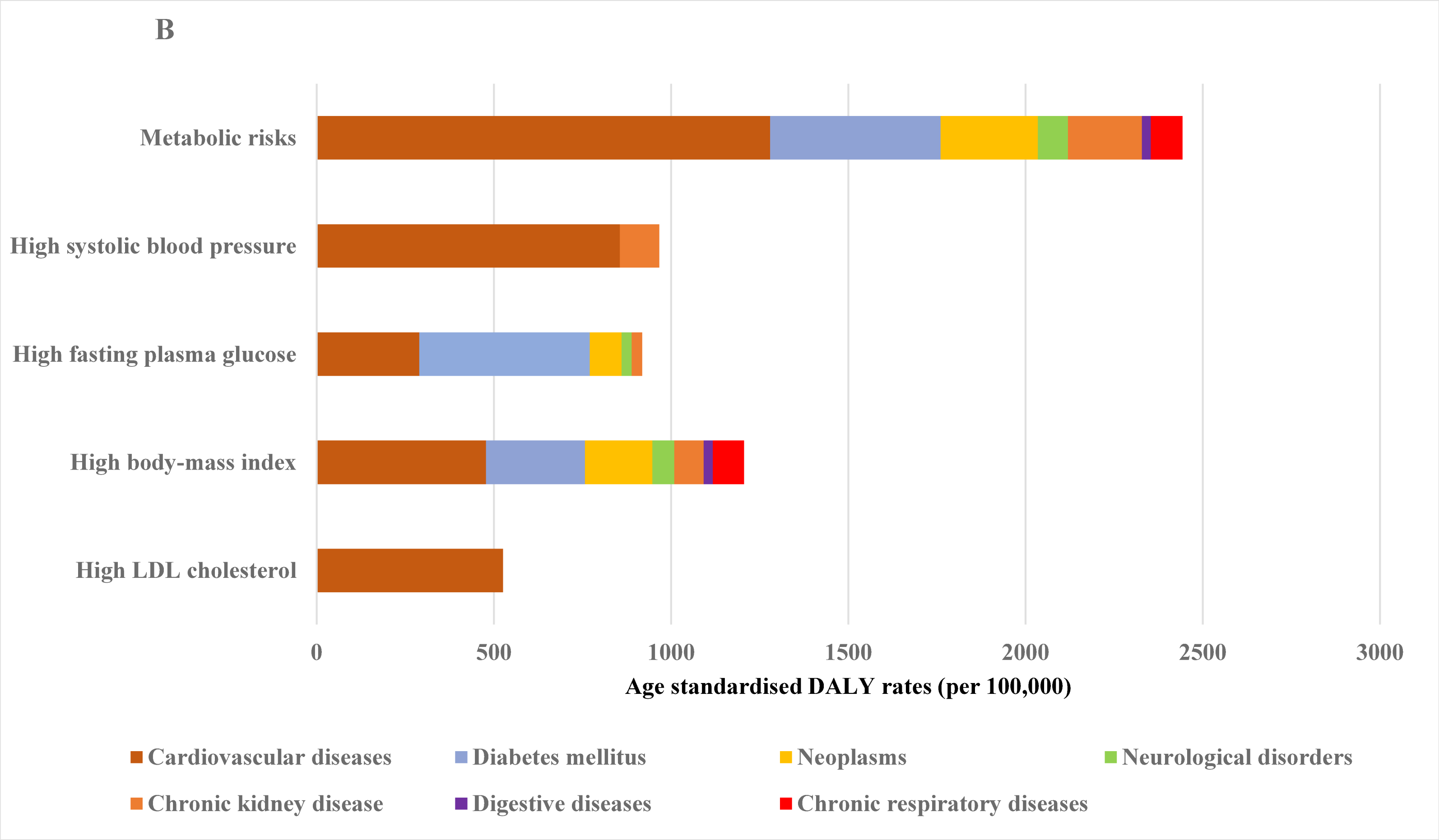

Supplementary Figure 5. Burden of specific NCDs attributable to individual and combined metabolic risks: age standardised death rates (A) and DALY rates (B) in Australia, 2019
