# Supplementary material for: Burdens of non-communicable disease attributable to metabolic risk factors in Australia, 1990–2019: joinpoint regression analysis of the Global Burden of Disease Study
Source: BMJ Open. 2023 Jul 14;13(7):e071319. doi: 10.1136/bmjopen-2022-071319 (PMC10351265; doi:10.1136/bmjopen-2022-071319)
Supplement: Author's manuscript [file bmjopen-2022-071319.draft_revisions.pdf]

BMJ Open

BMJ Open is committed to open peer review. As part of this commitment we make the peer review history of every article we publish publicly available.

When an article is published we post the peer reviewers' comments and the authors' responses online. We also post the versions of the paper that were used during peer review. These are the versions that the peer review comments apply to.

The versions of the paper that follow are the versions that were submitted during the peer review process. They are not the versions of record or the final published versions. They should not be cited or distributed as the published version of this manuscript.

BMJ Open is an open access journal and the full, final, typeset and author-corrected version of record of the manuscript is available on our site with no access controls, subscription charges or pay-per-view fees (http://bmjopen.bmj.com).

If you have any questions on BMJ Open's open peer review process please email info.bmjopen@bmj.com

BMJ Open

Trends and impacts of metabolic risk factors on non-communicable disease burdens in Australia, 1990–2019: Analysis of the Global Burden of Disease Study 2019

Journal:	*BMJ Open*
Manuscript ID	bmjopen-2022-071319
Article Type:	Original research
Date Submitted by the Author:	23-Dec-2022

Complete List of Authors:	Kibret, Kelemu; Deakin University, Faculty of Health<br>Backholer, Kathryn; Deakin University, Faculty of Health<br>Peeters, A; Deakin University, Faculty of Health<br>Tesfay, Fisaha; Deakin University, Faculty of Health<br>Nichols, Melanie; Deakin University, Faculty of Health

Keywords:	CARDIOLOGY, EPIDEMIOLOGY, PUBLIC HEALTH

SCHOLARONE™
Manuscripts

I, the Submitting Author has the right to grant and does grant on behalf of all authors of the Work (as defined in the below author licence), an exclusive licence and/or a non-exclusive licence for contributions from authors who are: i) UK Crown employees; ii) where BMJ has agreed a CC-BY licence shall apply, and/or iii) in accordance with the terms applicable for US Federal Government officers or employees acting as part of their official duties; on a worldwide, perpetual, irrevocable, royalty-free basis to BMJ Publishing Group Ltd ("BMJ") its licensees and where the relevant Journal is co-owned by BMJ to the co-owners of the Journal, to publish the Work in this journal and any other BMJ products and to exploit all rights, as set out in our licence.

The Submitting Author accepts and understands that any supply made under these terms is made by BMJ to the Submitting Author unless you are acting as an employee on behalf of your employer or a postgraduate student of an affiliated institution which is paying any applicable article publishing charge ("APC") for Open Access articles. Where the Submitting Author wishes to make the Work available on an Open Access basis (and intends to pay the relevant APC), the terms of reuse of such Open Access shall be governed by a Creative Commons licence – details of these licences and which Creative Commons licence will apply to this Work are set out in our licence referred to above.

Other than as permitted in any relevant BMJ Author's Self Archiving Policies, I confirm this Work has not been accepted for publication elsewhere, is not being considered for publication elsewhere and does not duplicate material already published. I confirm all authors consent to publication of this Work and authorise the granting of this licence.

For peer review only

Trends and impacts of metabolic risk factors on non-communicable disease burdens in Australia, 1990–2019: Analysis of the Global Burden of Disease Study 2019

Kelemu Tilahun Kibret[1*], Kathryn Backholer[1], Anna Peeters[1], Fisaha Tesfay[1], Melanie Nichols[1]

[1]Deakin University, Global Centre for Preventive Health and Nutrition, Institute for Health Transformation, School of Health and Social Development, Faculty of Health, 1 Gheringhap Street, Geelong, VIC 3220, Australia.

*Corresponding Author: kelemu.kibret@deakin.edu.au; 221 Burwood Highway, Burwood VIC 3125, Australia

For peer review only - http://bmjopen.bmj.com/site/about/guidelines.xhtml

Abstract

Objectives: We aimed to assess the trends and relative role of key metabolic risk factors on the total burden of non-communicable diseases in Australia over the last three decades.

Methods: The estimates of cause-specific NCD deaths and disability-adjusted life years (DALYs) attributable to key metabolic risks (high body mass index, fasting plasma glucose, blood pressure and cholesterol), were extracted from the Global Burden of Disease (GBD) 2019 study. Annual rates of change in NCD burden attributed to metabolic risk factors were estimated using a join-point regression model to assess trends over time.

Results: Between 1990 and 2019, the estimated absolute number of deaths from NCDs attributed to combined metabolic risks increased by 17%. However, the relative burden of NCDs related to metabolic risk in Australia decreased over the past three decades. In 2019, about one-third (34%) of NCD deaths and one-fifth (20%) of NCD DALYs were attributed to metabolic risk factors, compared to 42.9% and 24.4 % respectively in 1990. In 2019, cardiovascular diseases, neoplasms, and chronic kidney diseases were the most common NCD deaths attributed to metabolic risks. Of the metabolic risks, high body mass index accounted for the highest proportion of diabetes deaths (47%) and DALYs (58.1%) as well as chronic kidney diseases deaths (35.4%) and DALYs (39.7%). Similarly, high systolic blood pressure contributed to a high proportion of chronic kidney disease deaths (60.9%) and DALYs (53.2%), and CVD deaths (44%) and DALYs (46%).

Conclusion: Whilst the contribution of metabolic risk factors to the burden of NCDs have declined over time, their role in NCD death and disability remain a challenge as prevalence of these risk factors have increased. Prevention strategies should focus on metabolic risks particularly high body mass and index and high systolic blood pressure to substantially reduce NCD burden in the Australian population.

Key words: noncommunicable diseases; metabolic risk factors; deaths; disability adjusted life years, Australia

For peer review only - http://bmjopen.bmj.com/site/about/guidelines.xhtml

Strength and limitation of this study

- Application of longitudinal statistical method (Joinpoint regression analysis) which help to model the long-term trend of NCD burden attributed to metabolic risks
- The use of data from the GBD dataset, which is one of the most comprehensive and reliable data sources
- The likelihood that the metabolic risk related disease burden may vary between rural and urban areas, different regions or states, and socio-cultural conditions, which could not be characterised in this study.
- In GBD, the use of similar relative risks in certain age and gender groups in all countries may overestimate or underestimate the exact picture of disease burden, because metabolic risk factors may not influence disease outcomes in the same way in different population groups

Introduction

Currently, non-communicable diseases (NCDs) are the leading cause of mortality and morbidity worldwide, accounting for 71% of all deaths (1). In particular, cardiovascular diseases (CVDs) (e.g. ischemic heart disease and stroke) are leading causes of death in both high income and low and middle income countries (2, 3). Non-communicable diseases are multifactorial diseases caused by complex interactions of both behavioural and biological (metabolic) risk factors (4). Metabolic risk factors, including high systolic blood pressure (SBP), high body mass index (BMI) and high fasting plasma glucose (FPG) are among the top five leading risk factors (with smoking and unhealthy diet) for non-communicable diseases in the world (4). Globally, high SBP (SBP ≥ 130 mmHg) , high FPG (≥7 mmol/L), and high BMI (BMI ≥25 kg/m^2) were respectively responsible for 9.3%, 6.8% and 6.3% of disability-adjusted life years (DALYs) in 2019 (4). The largest proportion of disease burden in Australia in 2018 was attributed to metabolic risk factors - high body mass index (8.4%), high blood pressure (5.1%) and high blood plasma glucose (6.3%) (5).

Globally, the largest increases in the prevalence of modifiable risk factors from 2010 to 2019 were for metabolic risk factors such as high FPG and high BMI (4). According to the Australian National Health Survey 2017-18, the prevalence of high BMI (BMI ≥25 kg/m^2) was 67%, high blood pressure (SBP ≥ 130 mmHg or diastolic blood pressure (DBP) ≥ 90 mmHg was 22.8%,

For peer review only - http://bmjopen.bmj.com/site/about/guidelines.xhtml

high FPG was 5% in adults aged over 18 year (6). Further, 21% of individuals aged 65 years and over had high cholesterol level (≥240 mg/dL) (6).

NCDs have been recognised as a global priority in the United Nations' Sustainable Development Goals (SDG), which set a target of reducing premature death due to NCDs by one-third by 2030 (7). Similarly, the World Health Organization (WHO) has set a global target of a 25% reduction in premature death from CVDs, cancer, type 2 diabetes or chronic respiratory diseases by 2025, which can be achieved by reducing the burden of metabolic and behavioural risk factors using appropriate prevention approaches (8). A modelling study on the effect of risk factors on NCDs showed that premature mortality from the four main NCDs may be decreased worldwide by 22% in men and 19% in women if the SDG risk factor targets could be accomplished (9). A modelling study in Australia shows that a 25% relative reduction in premature deaths due to four NCDs in 2025, compared to 2010 levels, could be achieved if the age-standardised mortality rates continue to decrease by an average of 1.64% annually (10).

A clear understanding of the trends and impacts of metabolic risk factors for NCDs is necessary to prioritize prevention and control strategies to achieve NCD reduction goals. The evidence needs to be updated and tracked using the most recent available data (8, 11), and consistent, long-term and comparable findings at the national level are necessary. Assessing which risk factors notably increased or decreased over time and their impact on major NCDs is essential to prioritise and evaluate public health strategies and programmes at the regional or national level. Although some studies have examined the burden of diseases due to high BMI, blood glucose or cholesterol level in Australia covering short periods (5, 12, 13), long-term and comparative assessment of trends in NCD burden attributable to metabolic risk is still sparse. It is unclear which specific NCD burden attributed to metabolic risks has increased or decreased over time and for which metabolic risk factor. This study aims to investigate trends in the burden of NCDs attributable to metabolic risk factors in Australia between 1990 and 2019 using the latest Global Burden of Diseases (GBD) data. The objectives are to assess: 1) changes in exposure levels of metabolic risk factors in Australia between 1990 and 2019; 2) the burden of NCDs (deaths and DALYs) attributable to metabolic risk factors in 2019; 3) temporal trends in the burden of NCDs attributable to metabolic risk from 1990 to 2019.

Methods

Patient and public involvement

For peer review only - http://bmjopen.bmj.com/site/about/guidelines.xhtml

Patients or members of the public were not involved in this study

Data sources

The GBD 2019 study data were used for this study (4, 14). The GBD study is an ongoing global collaborative epidemiological study that comprehensively assesses the burden of diseases, injuries, and risk factors for 204 countries and territories by sex and age group, and currently includes data from 1990 to 2019. The GBD provides estimates of deaths and disability-adjusted life-years (DALYs) due to 369 diseases and injuries, and 87 risk factors (4, 14). The GBD study results can be used by global, regional, national, and local policy and decision-makers to gain insights into the burden and trends of population health challenges and optimise responses.

The GBD study incorporates data from many various data sources, including censuses, household surveys, vital statistics, disease registries, disease notifications, health service use, government and international websites and other sources. All the data sources used in GBD are available on the Global Health Data Exchange (GHDx) website (https://ghdx.healthdata.org/gbd-2019/data-input-sources). The collated data are used to generate standard epidemiological estimates, including prevalence, mortality, and DALYs, as well as the attributable burdens (mortality and DALYs) for specific risk factors using a comparative risk assessment framework. Through this framework, GBD investigators: 1) identify evidence-based risk–outcome pairs; 2) determine the exposure level associated with minimum risk, termed the theoretical minimum risk exposure level (TMREL); 3) estimate relative risks, population attributable fractions (PAFs) and attributable burdens (deaths and DALYs) for each risk factor-outcome pair. Detailed descriptions and methods used in GBD 2019 have been published elsewhere (4, 14).

Description of variables

Summary exposure value (SEV)

The GBD study estimated the exposure distributions for risk factors as a summary exposure value (SEV) (4). SEV is a relative risk-weighted prevalence of exposure, that measures the extent to which a population is exposed to a risk factor and the severity by which that risk contributes to the disease burden. SEV is reported on a scale of 0-100, where 0 means that no excess risk exists for a population, and 100 means that the population is at its highest risk. We

For peer review only - http://bmjopen.bmj.com/site/about/guidelines.xhtml

assessed the combined metabolic risks (high BMI, high SBP, high FPG and LDL cholesterol), and each individual metabolic risk.

Theoretical minimum risk exposure level (TMREL)

For each risk factor-outcome pair, the GBD study defines what level of exposure to a risk factor leads to the lowest amount of disease (4). The theoretical minimum risk exposure level value (TMREL) is the level of risk exposure associated with the lowest risk (4). Detailed TMREL estimation for each risk factor was described elsewhere (4). In the GBD, the TMREL for 'high body-mass index' for adults (ages 20+) was defined as BMI 20 – 25kg/m^2, and for children based upon International Obesity Task Force standards (15). The TMREL level for FPG is defined as 4.8-5.4 mmol/L; and for low-density lipoprotein (LDL) cholesterol the TMREL defined as 0.7–1.3 mmol/L. The TMREL level for SBP is defined as 110–115 mmHg (4).

Estimation of effect size, population attributable fractions and attributable burdens

The GBD 2019 evaluated the strength of epidemiological evidence on the causal relationship between each metabolic risk factor and disease outcome pair, using the World Cancer Research Fund evidence appraisal tool (16). For those risk factor-outcome pairs graded as having convincing or probable evidence, relative risks were estimated using data from meta-analysis of cohort, case-control and/or intervention studies (4). The outcome-specific burden of disease attributable to risk factors was determined by estimating the population attributable fraction (PAF) (4). PAF is the proportion of the disease burden that would be reduced in a population if exposure to a risk factor was equivalent to the level of TMREL. The PAFs provide estimates of the proportion of cases that might be prevented if a particular risk factor were to be reduced in the population. PAF incorporates the strength of association between risk factors and the outcome of interest, as well as the observed prevalence of risk factors in the population (17).

In the GBD study, the metrics used to quantify burden of disease were deaths and DALYs (3). DALYs are the sum of years of life lost and years lived with disability (14). For each risk-outcome pair, the attributable burden was estimated by multiplying the numbers of deaths or DALYs observed in the population by the PAF (4). In the GBD study, to remove the effect of age structure differences between populations, age-standardised rates (ASRs) (per 100,000) were estimated using the GBD world standard population, computed using population estimates for 2010–35 from the World Population Prospects (14, 18).

Data processing and statistical analysis

For peer review only - http://bmjopen.bmj.com/site/about/guidelines.xhtml

This study focused on the burden of NCDs attributable to individual metabolic risk factors (high BMI, high SBP, high FPG and high LDL cholesterol) and combined metabolic risks in Australia. We extracted numbers, proportions, and age-standardised rates (per 100,000) of metabolic risk-related deaths and DALYs and their 95% uncertainty intervals (UIs) of NCDs in Australia from 1990 to 2019 from the GBD study's GHDx results tool, (http://ghdx.healthdata.org/gbd-results-tool). We selected specific some NCDs (neoplasms, CVDs, diabetes mellitus, chronic kidney diseases (CKD), neurological disorders, chronic respiratory diseases (CRD)) that have summary estimates with common metabolic risk factors. We also extracted NCD deaths and DALYs attributable to metabolic risks by sex and age (20 years and over). Similarly, we extracted the age-standardised summary exposure values (SEV) for Australia for each metabolic risk factor from 1990 to 2019.

Using these extracted data from GBD results tool, the temporal trends in NCD burdens (deaths and DALYs) attributable to each metabolic risk factor over time (1990 to 2019) were assessed using the Joinpoint regression model (separately by sex) (19). This regression model segments the entire period and identifies the time points (years) at which there has been a significant change in trends (Joinpoints) (20). Then, the annual percentage change (APC) with its 95% confidence interval (CI) of rates between these inflection points is estimated. The average annual percentage change (AAPC) with its 95% CI for the entire period was also calculated, with respect to the underlying model fitted by the Joinpoint analysis. AAPC is estimated as a weighted average of the APCs using the Joinpoint segment lengths as weights (21). The AAPC is a summary measure of the trend for the entire period. If there are no Joinpoints identified, the model produces only one APC, which is equal to the overall AAPC.

We used the Grid Search Modelling Method with the default recommended parameter values - the minimum number of observations at either end of Joinpoint is two, and the minimum number of measurements between two Joinpoints is two. We specified a minimum of zero and a maximum of four Joinpoints and a Monte Carlo Permutation method was used to test significant changes in trends over the specified period. The permutation test was also used to determine whether APCs and AAPCs differed from zero using $p < 0.05$ as statistically significant (20). Data extracted from the GBD were processed in an excel sheet, and the trend analysis was carried out using the Joinpoint Regression Program version 4.9.1.0 (19).

For peer review only - http://bmjopen.bmj.com/site/about/guidelines.xhtml

Results

Trends in risk exposure for metabolic risk factors in Australia from 1990 to 2019

The age-standardised SEV of combined metabolic risks among the Australian population increased from 24.0 (95% UI: 19.1 - 29.7) in 1990 to 33.2 (95% UI: 27.4 -39.3) in 2019 (Supplementary Table 1). Most age standardised SEV of individual metabolic risk factors showed an increasing trend; the exposure to combined metabolic risk factors increased by 1.1% per year over the full period from 1990 to 2019, while the annual rate of increase was smaller in the most recent decade, at 0.8% from 2010 to 2019. Between 1990 and 2019, exposure to high BMI and high FPG increased by 1.5% and 2.0% per year, respectively, while exposure to high SBP decreased by 0.5% per year (Supplementary Table 1). The SEV of high FPG decreased in recent years (from 2017), with the APC estimated at 1.9% per year. Conversely, the SEV of high SBP increased, with an APC of 1.7% per year from 2012 onwards (Supplementary Fig.1).

The burden of NCDs attributable to metabolic risk factors in Australia

In 2019, metabolic risks were estimated to be responsible for about 34% of all NCD deaths (absolute numbers, n = 53369 (95% UI: 46725, 59687)) and 20% of NCD DALYs (n =1060720 (95% UI: 911391, 1228621) in Australia (Supplementary Table 2). The age-standardised proportions of metabolic risk-related NCD deaths were similar for both sexes in 2019; 32.5% (95% UI: 28.8,36.3) for males and 32.8% (95% UI: 28.2,37.2) for females (Table 1), but the age-standardised proportion of NCD DALYs attributed to metabolic risks was higher for males, 18.9% (95% UI: 16.4, 21.5) compared to females, 14.0% (95% UI: 11.8, 16.4) (Table 1). The age-standardised death rates of NCD linked with metabolic risks were 133.9 per 100,000 (95% UI: 118.6,149.9) for males and 94.7 per 100,000 (95% UI: 81.6,107.4) for females. Similarly, the metabolic risk-related age-standardised DALY rate was 3095.8 (2692.8, 3578.5) per 100,000 for males and 2198.7 (95% UI: 1827.9, 2623.3) per 100,00 for females in 2019 (Table 1).

For peer review only - http://bmjopen.bmj.com/site/about/guidelines.xhtml

Table 1. Numbers, age standardised rates and proportions of metabolic risk related NCDs death and DALYs by sex in Australia between 1990 and 2019.

	Number (95% UI)				Age standardised Proportion, % (95%UI)				Age standardised rate per 100,000 (95% UI)			
	1990	2010	2019	% Change 1990-2019	1990	2010	2019	% Change 1990-2019	1990	2010	2019	% Change 1990-2019
**Deaths**												
Male	23052 (21126, 24865)	21726 (19333, 24152)	26,399 (23330, 29621)	13.5 (10.4, 9.1)	41.6 (37.9, 5.1)	33.6 (30.0, 37.3)	32.5 (28.8, 36.3)	-20.2 (-23.9, -19.6)	302.0 (274.7, 327.6)	150.0 (133.7,166.7)	133.9 (118.6, 149.9)	-51.3 (-56.9, -54.3)
Female	22523 (20061, 24693)	23767 (20558, 26597)	26,970 (23156, 30516)	19.8 (15.43, 23.58)	43.7 (38.9, 48.0)	34.8 (30.2, 39.0)	32.8 (28.2, 37.2)	-25.0 (-27.5, -22.5)	199.8 (177.6, 219.0)	107.2 (93.2, 120.2)	94.7 (81.6, 107.4)	52.6 (-54.1, -51.0)
Both	45575 (41530, 49465)	45492 (40228, 50512)	53369 (46725, 59687)	17.1 (12.51, 20.67)	43.0 (38.8, 46.8)	34.4 (30.5, 38.2)	32.8 (28.7, 36.6)	-23.8 (-26.0, -21.8)	245.3 (221.7, 266.8)	127.3 (112.6, 141.1)	113.3 (99.5, 126.6)	-53.8 (-55.1, -52.6)
**DALYs**												
Male	524488 (478223, 573378)	482011 (421511, 548065)	571369 (497917, 659110)	8.9 (4.1, 15.0)	26.6 (23.8, 29.6)	19.4 (17.0, 22.1)	18.9 (16.4, 21.5)	-30.0 (-31.0, -27.5)	6124.6 (5580.6, 6704.6)	3,321.8 (2903.8, 3787.0)	3095.8 (2692.8, 3578.5)	-49.5 (-51.8, -46.6)
Female	411233	425811	489350	19.0	19.6	14.6	14.0	-28.8 (-	3,764.7	2,351.2	2198.7	-41.6 (-45.4,

For peer review only - http://bmjopen.bmj.com/site/about/guidelines.xhtml

	(366423, 458999)	(362905, 493866)	(411090, 574086)	(12.2, 25.1)	(16.8, 22.6)	(12.28, 16.9)	(11.8, 16.4)	29.7, -27.3)	(3346.1, 4212.5)	(1973.1, 2770.7)	(1827.9, 2623.3)	-37.7)
Both	935,721 (846039, 1031253)	907822 (786590, 1040661)	1,060719 (911391, 1228621)	13.4 (7.7, 19.1)	23.4 (20.6, 26.4)	17.0 (14.7, 19.6)	16.4 (14.15, 19.0)	-29.7 (-31.1, -28.1)	4,870.9 (4402.1, 5377.5)	2818.2 (2439.8, 3246.5)	2,630.3 (2,246.3, 3063.7)	-46.0 (-49.0, -43.0)

For peer review only

Age-specific rates and proportion of NCD deaths and DALYs related to metabolic risks increased with age in both sexes (Figures 2A, 2B). The age specific NCD deaths attributable to metabolic risks were higher among males than females (Figure 2A). Metabolic risk related NCD deaths has an increasing trend across men and women aged 20 years and over, except for the over 95 years age group (Figure 2A). Similarly, metabolic risk attributable NCD DALY rates increased with age and were higher in males than females between the ages of 30 and 90 years. (Figure 2B).

From the metabolic risk factors examined, high SBP and high BMI were the largest contributors to NCD deaths and DALYs. An estimated 16.4% of NCDs deaths (n = 25,498) and 8% of NCD DALYs (n = 406,445) were due to high SBP, while BMI was estimated to contribute to 12.0% of NCDs deaths (n=18,713) and 10% of NCD DALYs (n=525,135) (Supplementary Table 2). High FPG was also a significant contributor to NCD deaths (11.4%) and DALYs (7.2%), and the values were lower for high LDL (Supplementary Table 2).

Temporal trends of metabolic risk related NCD burden from 1990 to 2019 in Australia

Between 1990 and 2019, the estimated absolute number of NCD deaths attributed to combined metabolic risks increased by 17%, from 45,575 (95% UI: 41530, 49465) to 53,369 (46725, 59687) (Table 1 and Supplementary Table 1). Similarly, the number of metabolic risks related NCD DALYs increased by 13.4%, from 935721 (95% UI: 846039, 1031253) in 1990 to 1,060,719.82 (95% UI: 911391,1228621) DALYs in 2019 (Table 1, Supplementary Figure 2). However, the ASRs of NCD deaths and DALYs attributable to metabolic risks roughly halved between 1990 and 2019, with AAPCs of -2.6% (95% CI: -2.8, -2.5) and -2.1% (95% CI: -2.2, -2.0) respectively (Tables 1 & 2, Supplementary Table 1).

The proportions and ASRs of NCD deaths and DALYs related to metabolic risks have decreased over time for both males and females. The proportion of deaths attributable to metabolic risks were higher for females than males, and the ASRs were higher for males than females (Figure 3A & 3B, Table 1). Both the proportion and ASRs of metabolic risk attributable DALYs were higher among men (Table 1 & Figure 3C & D). The proportion of deaths attributable to metabolic risks for females significantly decreased by 0.5% 1.5% and 1.2% per year from 1990 to 1995, 1995 to 2003 and from 2003 to 2014 respectively. Similarly, the proportion of deaths attributable to metabolic risks in males significantly declined by 0.9%,

For peer review only - http://bmjopen.bmj.com/site/about/guidelines.xhtml

1.9% and 1.1% per year from 1990 to 2003, 2003 to 2006 and 2006 to 2014 respectively, but no change between 2014 and 2019 (Figure 3B).

Over the entire study period, the proportion of NCD deaths attributable to BMI and FPG increased slightly, with AAPCs of 0.2% (0.1%, 0.2%) and 0.7% (0.5%, 0.9%) respectively. In contrast, as prevalence of SBP and LDL decreased, the proportion of NCD deaths related to SBP and LDL significantly decreased, with AAPCs of -1.9% (-2.0%, -1.8%) and -2.2% (-2.3, -2.1%) respectively, even though plateaued in more recent years, demonstrated by the APCs between 2013-2019, which were not different from zero for either risk factor (Figure 1B). There was a significant decrease in age-standardised death rates attributable to high SBP (AAPC: -3.6 (95% CI: (-3.8, -3.5)), and high LDL cholesterol (AAPC: -3.9 (95% CI: -4.1, -3.7)), which resulted in 65.4% (95% UI: -67.0, -63.61)) and 68.4% (95% UI: -70.1, -67.2) total decline respectively (Table 2&3). Similarly, ASRs for NCD DALYs attributable to high SBP and high LDL decreased by an average 3.6% (-3.8, -3.5) and 4.1% (-4.4, -3.8) per year, which resulted in a 65.8% and 70.1% total reduction from 1990 to 2019, respectively (Table 2 & 3).

For peer review only - http://bmjopen.bmj.com/site/about/guidelines.xhtml

Table 2. The age-standardised death rates and disability adjusted life years (DALYs) rates of non-communicable diseases (NCDs) attributable to metabolic risks and their temporal trends between 1990 to 2019 by sex.

Risk factors	sex	Deaths per 100,000(95% UI)			DALYs per 100,000(95% UI)		
		1990	2019	AAPC (1990-2019)	1990	2019	AAPC (1990-2019)
Metabolic risk factors	Both	245.3 (221.7, 266.8)	113.3 (99.5, 126.6)	-2.6 (-2.8, -2.5) *	4870.9 (4402.1, 5377.5)	2630.3 (2246.3, 3063.7)	-2.1 (-2.2, -2.0) *
	Male	302.0 (274.7, 327.6)	133.9 (118.6, 149.9)	-2.8 (-2.9, -2.6) *	6124.6 (5580.6, 6704.6)	3095.8 (2692.8, 3578.5	-2.3 (-2.4, -2.2) *
	Female	199.8 (177.6, 219.0)	94.7 (81.5, 107.4)	-2.6 (-2.7, -2.4) *	3764.7 (3346.0, 4212.5)	2198.7 (1827.9, 2623.3)	-1.8 (-1.9, -1.7) *
High systolic blood pressure	Both	155.6 (131.2, 177.0)	53.8 (43.4, 64.4)	-3.6 (-3.8, -3.5) *	2822.6 (2506.5, 3140.8)	966.5 (824.7, 1111.5)	-3.6(-3.8, -3.5) *
	Male	67.9 (47.5, 98.8)	47.9 (33.0, 67.8)	-3.7 (-3.9, -3.5) *	1292.4 (1005.1, 1698.2)	1138.0 (878.9, 1451.8)	-3.7 (-3.9, -3.5) *
	Female	39.1 (27.6, 55.8)	28.3 (18.7, 42.)	-3.6 (-3.8, -3.4) *	784.2 (606.7, 1006.7	725.1 (538.8, 961.3)	-3.5 (-3.7, -3.4) *
High? Body mass index	Both	65.3 (38.2, 95.6)	41.7 (27.0, 57.5)	-1.5 (-1.7, -1.3) *	1801.0 (1102.2, 2556.7)	1387.7 (954.7, 1855.0)	-0.9 (-0.9, -0.8) *
	Male	75.6 (42.8, 113.0)	45.9 (27.9, 65.7)	-1.7 (-1.9, -1.6) *	2091.4 (1252.7, 3014.0)	1519.1 (1008.5, 2063.2)	-1.1 (-1.1, -1.0) *
	Female	55.1 (33.1, 81.4)	36.9 (24.4, 50.7)	-1.4 (-1.6, -1.1) *	1511.5 (947.8, 2146.6)	1255.7 (874.5, 1670.6	-0.6 (-0.7, -0.6) *
High fasting plasma glucose	Both	50.2 (35.9, 71.0)	37.2 (25.7, 53.1)	-0.9 (-1.2, -0.7) *	1001.0 (782.8, 1275.1)	919.9 (698.2, 1187.71)	-0.2 (-0.4, -0.1) *
	Male	67.9 (47.5, 98.8)	47.9 (33.0, 67.8)	-1.2 (-1.3, -1.0) *	1292.4 (1005.1, 1698.2)	1138.1 (878.9, 1451.9)	-0.4 (-0.5, -0.2) *
	Female	39.1 (27.6, 55.8)	28.3 (18.7, 42.1)	-1.0 (-1.3, -0.7) *	784.2 (606.7, 1006.7)	725.1 (538.8, 961.3)	-0.3 (-0.5, -0.0) *
High LDL cholesterol	Both	94.9 (70.4, 122.1)	30.0 (21.1, 40.1)	-3.9 (-4.1, -3.7) *	1758.0 (1443.6, 2100.7)	525.2 (420.9, 639.0)	-4.1 (-4.4, -3.8) *
	Male	124.9 (95.7, 157.5)	38.3 (27.9, 49.9)	-4.0 (-4.2, -3.8) *	2467.1 (2060.7, 2905.8)	732.6 (601.9, 876.7)	-4.1 (-4.4, -3.8) *
	Female	70.4 (49.1, 94.7)	22.5 (14.6, 31.7)	-3.9 (-4.1, -3.6) *	1116.1 (855.4, 1393.3)	331.9 (249.8, 430.0)	-4.1 (-4.3, -3.9) *

LDL- low-density lipoprotein, AAPC – Average annual percentage change, *AAPC is significantly different from zero at the alpha = 0.05 level

For peer review only - http://bmjopen.bmj.com/site/about/guidelines.xhtml

Specific type of NCD deaths and DALYs attributable to metabolic risk factors

In 2019, cardiovascular disease deaths (34,921) and DALYs (537319) attributed to metabolic risks were the highest, followed by neoplasms with 5644 deaths and 110,091 DALYs and chronic kidney diseases with 5207 deaths and 86,550 DALYs. (Supplementary Table 3). The age-standardised proportion of diabetes mellitus and chronic kidney disease deaths was 100% attributed to metabolic risks in Australia. Similarly, a high proportion of age-standardised death rates of CVDs (68%), neurological disorders (16.6%) and neoplasms (10.3%) were attributed to combined metabolic risks (Table 3).

Each metabolic risk factor was responsible for a sizeable proportion of age-standardised death and DALY rates of specific NCDs (Table 3 and Supplementary Figure 3). About 47.0% deaths and 58.1% DALYs of diabetes mellitus, and about 35.4% deaths and 39.7% DALYs of CKD were attributed to high BMI. Similarly, a significant proportion of CVD deaths, 18.61% (95% UI: 11.8, 26.1) and DALYs, 25.5% (95% UI: 17.6, 33.7) were attributable to high BMI (Table 3). The results show that 100% diabetes mellitus deaths and DALYs was attributable to high FPG. Furthermore, about 18.6% (11.0, 31.0) of deaths and 15.5% (95% UI: 10.1, 23.8) of DALY for CVDs related to high FPG (Table 3). High SBP was responsible for 60.9% deaths and 53.2% DALYs of CKD, and 44% deaths and 46% DALYs of CVD. In addition, 27.7% of cardiovascular disease deaths and 28.0% of DALYs were attributable to high LDL cholesterol (Table 3).

For peer review only

For peer review only - http://bmjopen.bmj.com/site/about/guidelines.xhtml

Table 3. Burden of specific NCDs (deaths and DALYs) attributable to metabolic risks in Australia, 2019.

Specific Causes of NCDs	Deaths		DALYs	
	Proportion, % (95% UI)	ASRs per 100,000(95% UI)	Proportion, % (95% UI)	ASR per 100,000(95% UI)
**Combined metabolic risks**				
Neoplasms	10.3 (6.0, 15.61)	13.0 (7.5, 19.9)	9.5 (5.7, 14.1)	274.2 (164.4, 407.6)
Cardiovascular diseases	68.1 (61.6, 73.8)	73.6 (62.2, 82.8)	68.3 (63.0, 72.9)	1279.8 (1145.9, 1410.1)
Chronic respiratory diseases	1.6 (1.0, 2.3)	0.4 (0.2, 0.6)	9.3 (6.1, 13.1)	88.5 (54.1, 138.7)
Neurological disorders	16.6 (5.9, 29.2)	5.8 (1.0, 16.6)	7.02 (2.5, 14.8)	85.1 (26.4, 202.8)
Diabetes mellitus	100.0 (100.0, 100.0)	9.2 (8.2, 10.0)	100.0 (100.0, 100.0)	480.8 (365.6, 613.0)
Chronic kidney disease	100.0 (100.0, 100.0)	10.8 (9.0, 12.1)	100.0 (100.0, 100.0)	208.5 (184.8, 233.2)
**High body mass index**				
Neoplasms	6.7 (4.1, 9.5)	8.4 (5.2, 12.0)	6.6 (4.1, 9.2)	190.3 (118.8, 265.9)
Cardiovascular diseases	18.6 (11.8, 26.1)	20.1 (12.7, 28.4)	25.5 (17.6, 33.7)	477.8 (327.8, 641.5)
Chronic respiratory diseases	1.6 (1.0, 2.3)	0.4 (0.2, 0.6)	9.3 (6.12, 13.1)	88.5 (54.1, 138.7)
Neurological disorders	11.8 (3.8, 23.2)	4.1 (0.7, 12.2)	5.1 (1.5, 11.4)	61.6 (17.4, 152.5)
Diabetes mellitus	47.0 (31.9, 62.2)	4.3 (2.9, 5.9)	58.1 (44.9, 68.6)	279.5 (192.1, 383.4)
Chronic kidney disease	35.4 (16.5, 55.5)	3.8 (1.8, 6.0)	39.7 (25.4, 54.5)	82.7 (52.6, 116.2)
**High fast plasma glucose**				
Neoplasms	3.8 (1.0, 7.9)	4.8 (1.3, 9.9)	31 (0.8, 6.4)	89.1 (23.9, 184.0)
Cardiovascular diseases	18.6 (11.0, 31.0)	20.1 (11.8, 33.4)	15.5 (10.1, 23.8)	289.8 (186.2, 455.0)

For peer review only - http://bmjopen.bmj.com/site/about/guidelines.xhtml

Neurological disorders	5.9 (1.1, 14.3)	2.1(0.2, 7.8)	2.4(0.4, 6.7)	29.1 (5.1, 95.7)
Diabetes mellitus	100.0 (100.0, 100.0)	9.2 (8.2, 10.0)	100.0 (100.0,100.0)	480.8 (365.6, 613.0)
Chronic kidney disease	9.5 (6.6, 13.6)	1.0 (0.7, 1.5)	13.9 (11.1, 17.0)	29.1 (22.1, 37.3)
**High Systolic blood pressure**				
Cardiovascular diseases	43.7 (36.2, 51.6)	47.3 (37.5, 57.3)	45.7 (39.6, 51.3)	855.6 (720.6, 989.9)
Chronic kidney disease	60.9 (52.7, 68.2)	6.5 (5.3, 7.7)	53.2 (46.0, 59.7)	110.9 (92.3, 131.4)
**High LDL cholesterol**				
Cardiovascular diseases	27.71 (20.0, 36.3)	30.0 (21.1, 40.1)	28.0 (23.0, 33.9)	525.2 (420.9, 639.0)

ASR- Age-standardized rate; DALYs- Disability adjusted life years; NCDs – Non-communicable diseases, UI- uncertainty interval

For peer review only

For peer review only - http://bmjopen.bmj.com/site/about/guidelines.xhtml

Trends of specific NCD deaths and DALYs attributable to metabolic risks

Over the last three decades (1990-2019), the metabolic risk-related age-standardised death rates of CVDs, chronic respiratory diseases and diabetes mellitus significantly declined by an average 3.5%, 3.7% and 0.8% per year (Table 4). In contrast, age-standardised death rates of neoplasms, neurological disorders and chronic kidney disease attributed to metabolic risks significantly increased by 0.5%, 1.0% and 0.5% per year, respectively (Table 4). Specifically, age-standardised death rates of chronic kidney disease related to BMI and FPG significantly increased by 1.1% and 2.0% per year, and death rates of neurological disorders linked to BMI and FPG increased by 1.0% and 0.9% per year. In contrast, age-standardised CVD death rates attributable to high BMI, high FPG, high SBP and high LDL cholesterol decreased by 2.8%, 1.5%, 3.9%, and 3.9% per year, respectively, between 1990 and 2019 (Table 4).

For peer review only - http://bmjopen.bmj.com/site/about/guidelines.xhtml

Table 4. Trends in metabolic risk-related death rates of specific NCDs (age standardised rates) in Australia, 1990 – 2019

Specific Causes of NCDs	Age-standardized death rate (95% UI) per 100,000)			AAPC (95% CI)	
	1990	2010	2019	1990-2019	2010-2019
**Metabolic risks combined**					
Neoplasms	11.4 (6.118.0)	13.1 (7.6, 20.0)	13.0 (7.5, 19.9)	0.5 (0.4, 0.6) *	0.0 (-0.0, 0.1)
Cardiovascular diseases	206.8 (185.1, 224.5)	86.2 (74.6, 96.2)	73.6 (62.2, 82.8)	-3.5 (-3.7, -3.3) *	-1.9 (-2.2, -1.5) *
Chronic respiratory diseases	1.1 (0.6, 1.8)	0.4 (0.3, 0.6)	0.4 (0.2, 0.6)	-3.7 (-4.3, -3.1) *	-1.0 (-1.2, -0.8) *
Neurological disorders	4.5 (0.7, 13.3)	5.8 (1.0, 16.7)	5.8 (1.0, 16.6)	1.0 (0.9, 1.0) *	-0.0 (-0.1, 0.0)
Diabetes mellitus	11.6 (10.7, 12.1)	11.1 (9.9, 11.8)	9.2 (8.2, 10.0)	-0.8 (-1.0, -0.5) *	-2.1 (-2.7, -1.4) *
Chronic kidney disease	9.5 (8.6, 10.1)	10.1 (8.7, 11.0)	10.8 (9.03, 12.1)	0.5 (0.2, 0.9) *	0.7 (0.5, 0.9) *
**High body mass index**					
Neoplasms	7.5 (4.2, 11.4)	8.3 (5.1, 11.8)	8.5 (5.2, 12.0)	0.4 (0.3, 0.5) *	0.1 (0.1, 0.2) *
Cardiovascular diseases	45.6 (26.2, 68.1)	22.5 (14.1, 32.0)	20.1 (12.7, 28.4)	-2.8 (-3.0, -2.6) *	-1.4 (-1.7, -1.1) *
Chronic respiratory diseases	2.8 (1.4, 4.5)	3.5 (1.6, 5.5)	0.4 (0.2, 0.6)	-3.7 (-4.3, -3.1) *	-1.0 (-1.2, -0.8) *
Neurological disorders	3.1 (0.5, 9.5)	4.0 (0.7, 11.8)	4.1 (0.7, 12.2)	1.1 (1.0, 1.0) *	0.3 (0.2, 0.5) *
Diabetes mellitus	4.8 (3.1, 6.6)	5.1 (3.4, 6.9)	4.3 (2.9, 5.9)	-0.4 (-0.6, 0.1) *	-2.0 (-2.6, -1.4) *
Chronic kidney disease	2.8 (1.4, 4.5)	3.5 (1.6, 5.5)	3.8 (1.8, 6.0)	1.1 (0.8, 1.4) *	0.9 (0.5, 1.4) *
**High fast plasma glucose**					
Neoplasms	4.1 (1.1, 8.5)	5.1 (1.4, 10.5)	4.8 (1.3, 9.9)	0.6 (0.4, 0.8) *	-0.6 (-1.1, -0.0) *
Cardiovascular diseases	32.4 (19.9, 53.5)	26.0 (14.8, 44.2)	20.1 (11.8, 33.4)	-1.5 (-1.8, -1.2) *	-2.5 (-2.9, -2.1) *

For peer review only - http://bmjopen.bmj.com/site/about/guidelines.xhtml

Neurological disorders	1.6 (0.2, 6.3)	2.2 (0.2, 8.4)	2.1 (0.2, 7.8)	0.9 (0.8, 0.9) *	-0.8 (-1.0, -0.7) *
Diabetes mellitus	11.6 (10.7, 12.1)	11.1 (9.9, 11.8)	9.2 (8.2, 10.0)	-0.8 (-1.0, -0.5) *	-2.1 (-2.7, -1.4) *
Chronic kidney disease	0.6 (0.4, 0.8)	1.0 (0.9, 1.2)	1.0 (0.7, 1.5)	2.0 (1.7, 2.2) *	-0.4 (-0.6, -0.1) *
**High Systolic blood pressure**					
Cardiovascular diseases	149.6 (125.5, 170.5	54.7 (44.2, 65.5)	47.3 (37.5, 57.3)	-3.9 (-4.1, -3.7) *	-1.8 (-2.2, -1.5) *
Chronic kidney disease	6.1 (5.2, 6.9)	6.2 (5.2, 7.1)	6.5 (5.3, 7.7)	0.3 (-0.1, 0.6)	0.6 (0.4,0.8)
**High LDL cholesterol**					
Cardiovascular diseases	94.9 (70.4, 122.1	35.5 (24.8, 47.7)	30.0 (21.1, 40.1)	-3.9 (-4.1, -3.7) *	-2.0 (-2.4, -1.6) *

AAPC- Average annual percent change; LDL-low density lipoprotein; NCDs – Non-communicable diseases; UI- uncertainty intervals

*AAPC is significantly different from zero at the α = 0.05 levels

For peer review only

For peer review only - http://bmjopen.bmj.com/site/about/guidelines.xhtml

The metabolic risk-related age-standardised DALY rates for CVD and chronic respiratory diseases significantly decreased by 3.6% and 0.6% per year over this period, whereas metabolic risk-related DALY rates of neoplasms, neurological disorders, diabetes mellitus and chronic kidney disease increased by 0.4%, 1.0%, 0.6% and 0.3% per year, respectively. However, the decline of metabolic risk-related CVD DALY rates in the past decade (2010-2019) (1.7% per year) were slow compared to over the last 30 years (3.6% per year) (Table 5).

Similarly, age-standardised DALY rates of CVD attributable to High BMI, high FPG, high SPB and high LDL cholesterol declined by 2.7%, 1.5%, 3.9% and 4.1% per year between 1990 and 2019. But between 2010 and 2019, the rate of decline has slowed with an average annual rate of change of 1.2%, 1.9%, 1.6%, and 2.0% for high BMI, high FPG, high SPB and high LDL cholesterol, respectively (Table 5).

For peer review only

For peer review only - http://bmjopen.bmj.com/site/about/guidelines.xhtml

Table 5. Trends in metabolic risk-attributed DALY rates of specific NCDs (age standardised rates) in Australia

	Age-standardized DALY rates (95% UI) per 100,000)			AAPC (95% CI)	
Specific Causes of NCDs	1990	2010	2019	1990-2019	2010-2019
**Combined metabolic risks**					
Neoplasms	247.4 (136.6, 379.1)	274.8 (162.7, 410.0)	274.2 (164.4, 407.6)	0.4 (0.3, 0.4) *	-0.0 (-0.1, 0.0)
Cardiovascular diseases	3689.4 (3407.5, 3945.1)	1479.7 (1338.2, 1615.6)	1279.8 (1145.9, 1410.1)	-3.6 (-3.8, -3.4) *	-1.7 (-2.0, -1.4) *
Chronic respiratory diseases	104.0 (58.7, 167.7)	83.7 (50.8, 127.7)	88.52 (54.1, 138.7)	-0.6 (-0.8, -0.4) *	0.9 (0.7, 1.1) *
Neurological disorders	65.0 (18.1, 161.8)	84.6 (26.5, 198.8)	85.1 (26.4, 202.8)	1.0 (0.9, 1.0) *	0.1 (0.0, 0.2) *
Diabetes mellitus	409.5 (343.5, 486.2)	484.9 (386.0, 610.1)	480.8 (365.6, 613.0)	0.6 (0.5, 0.6) *	-0.1 (-0.6, 0.5)
Chronic kidney disease	195.4 (178.0, 214.3)	198.5 (177.7, 220.9)	208.5 (184.8, 233.2)	0.3 (0.0, 0.5) *	0.5 (-0.2, 1.3) *
**High body mass index**					
Neoplasms	173.6 (99.7, 260.5)	187.5 (116.6, 264.1)	190.3 (118.8, 265.9)	0.3 (0.2, 0.4) *	0.1 (0.0, 0.2) *
Cardiovascular diseases	1057.9 (633.0, 1511.9)	532.5 (361.1, 716.4)	477.8 (327.8, 641.5)	-2.7 (-3.0, -2.5) *	-1.2 (-1.5, -0.1) *
Chronic respiratory diseases	104.0 (58.7, 167.7)	83.7 (50.8, 127.7)	88.5 (54.1, 138.7)	-0.6 (-0.8, -0.4) *	0.9 (0.7, 1.1) *
Neurological disorders	46.3 (11.4, 120.2)	59.4 (16.6, 144.5)	61.6 (17.4, 152.5)	1.0 (0.9, 1.0) *	0.4 (0.3, 0.5) *
Diabetes mellitus	198.7 (134.2, 270.9)	270.2 (187.7, 366.0)	279.5 (192.1, 383.4)	1.2 (1.0, 1.5) *	0.5 (0.4, 0.5) *
Chronic kidney disease	64.6 (37.0, 95.1)	76.0 (47.0, 106.9)	82.7 (52.6, 116.2)	0.9 (0.7, 1.1) *	0.9 (0.2, 1.6) *
**High fast plasma glucose**					
Neoplasms	76.9 (19.7, 161.3)	92.2 (24.6, 189.4)	89.1 (23.9, 184.0)	0.6 (0.5, 0.7) *	-0.2 (-0.3, 0.2) *

For peer review only - http://bmjopen.bmj.com/site/about/guidelines.xhtml

Cardiovascular diseases	471.5 (314.5, 714.9)	353.4 (218.7, 561.1)	289.8 (186.2, 455.0)	-1.5 (-1.8, -1.3) *	-1.9 (-2.3, -1.5) *
Neurological disorders	21.7 (3.5,74.0)	30.9 (5.2, 101.0)	29.1 (5.1, 95.7)	1.0 (0.9, 1.1) *	-0.7 (-0.8, -0.5) *
Diabetes mellitus	409.5 (343.5, 486.2)	484.9 (386.0, 610.1)	480.8 (365.6, 613.0)	0.6 (0.5, 0.6) *	-0.1 (-0.1, 0.0)
Chronic kidney disease	19.9 (15.9, 24.8)	29.3 (24.8, 35.1)	29.1 (22.1, 37.3)	1.3 (1.3, 1.4) *	0.1 (-0.1, 0.2)
**High Systolic blood pressure**					
Cardiovascular diseases	2713.3 (2402.2, 3033.0)	974.9 (839.3, 1112.3)	855.6 (720.6, 989.9)	-3.9 (-4.1, -3.8) *	-1.6 (-1.9, -1.3) *
Chronic kidney disease	109.3 (92.8, 126.6)	104.7 (88.6, 120.8)	110.9 (92.3, 131.4)	0.1 (-0.0, 0.3)	0.7 (0.6, 0.9) *
**High LDL cholesterol**					
Cardiovascular diseases	1757.9 (1443.6, 2100.7)	623.8 (504.1, 761.0)	525.2 (420.9, 639.0)	-4.1 (-4.4, -3.8) *	-2.0 (-2.3, -1.7) *

AAPC- Average annual percent change; LDL-low density lipoprotein; NCDs – Non-communicable diseases; UI- uncertainty intervals

*AAPC is significantly different from zero at the alpha = 0.05 levels

For peer review only - http://bmjopen.bmj.com/site/about/guidelines.xhtml

Discussion

We assessed the trends and impact of metabolic risk factors on the burden of NCDs in the Australian population across age and sex between 1990 and 2019. Although the relative burden of metabolic risk related NCDs in Australia has declined over the last three decades, metabolic risks continue to account for a significant proportion, and absolute number, of the NCD burden. Metabolic risk related NCD deaths and DALYs varied according to sex. In 2019, the relative proportion of NCD DALYs attributed to metabolic risks was higher for males (18.9%) than females (14.0%). Approximately one-third (34%) of NCD deaths and one-fifth (20%) of NCD DALYs were attributed to metabolic risk factors in 2019. Over two-thirds (68%) of CVD deaths and most deaths of type 2 diabetes and chronic kidney diseases were also related to metabolic risk factors.

The age-standardised rate of deaths and DALYs attributed to metabolic risks decreased over the last three decades, although the absolute number of metabolic risk-related deaths and DALYs have increased. The reductions in age standardised NCD burdens attributed to metabolic risks could be because of enhancement in prevention programs, and the availability of effective treatments (22-24). In addition, the increase in NCD burden due to increased prevalence of BMI and FPG could offset by reduced risks from SBP, and LDL. The rise in the absolute number of metabolic risk-related NCD burdens could be due to the growing population size and aging population in Australia and advances in diagnostic methods that help diagnose the disease at its early stages (25). The incidence of many NCDs increases with age and many older individuals now live with chronic health conditions (6). In addition to the metabolic risk factors, other non-metabolic factors (12) might contribute to the NCDs burden in Australia. For instance, dietary risks and tobacco significantly contributed for the burden of NCD in 2018 and are risk factors themselves for the metabolic risks we examined in this study (5).

We show that NCD deaths and DALYs related to metabolic risks differed substantially by sex. The burden of NCDs attributed to metabolic risks was higher for males than for females and decreased overtime for both sexes at different rate. This might be due to the difference in risk factor distributions among genders (e.g. proportion of overweight is higher in males (75%) than females (60%), and males had higher smoking rates (16.5%) than females (11%) (6).

High BMI, SBP and FPG were responsible for high proportions of metabolic risk attributable NCD burden in 2019, each accountable for 10-16% of age standardised NCD deaths, and 5-

For peer review only - http://bmjopen.bmj.com/site/about/guidelines.xhtml

8% of NCD DALYs. This is consistent with the results of the studies that show a high burden of NCD deaths was associated with high BMI and high SBP in Australian population in 2018 (5, 12). Metabolic risk factors are one of the health risk factors targeted for NCD prevention in the current Australian NCD National Framework (24). Metabolic risk factors along with other behavioural risk factors are important in Australia: nearly 90% of individuals over 45 years of age had at least one risk factor, and two-thirds of them had over three risk factors in 2012 (26). The prevalence of high BMI, SBP and FPG was also high in the Australian population in 2017/18 (6).

Our analysis of 2019 GBD data showed that high SBP, BMI, high FPG and high LDL cholesterol were ranked first, third (behind smoking), fourth and fifth among the top five leading risk factors of NCD deaths in Australia, respectively (27). BMI (first), SBP (third, behind smoking) and FPG (fourth) were the leading risk factors for NCD DALYs in the country (27). This metabolic risk related NCD burden is evident worldwide (28, 29), with evidence showing that a considerable proportion of NCD deaths is attributed to high blood pressure (13%), high blood sugar (6%), and overweight (5%) (30).

Our study showed that the proportion of the age standardised NCD death rates related to BMI and FPG slightly increased, while the proportion of NCDs attributable to SBP and LDL decreased, between 1990 to 2019. In line with these findings, BMI and FPG were the significant contributors to NCD deaths in Australia in 2018 (5, 12). The recent Australian Health Survey also revealed that the prevalence of high BMI has increased from 63% in 2014-15 to 67% in 2017-18 in the adult population (6). The other study findings also show that the proportions of individuals with obesity, high cholesterol, and high blood glucose increased between the year 2004 to 2013 (31). Sub-optimal dietary intake and low physical activity levels are risk factors for the metabolic risks examined in our study and therefore also NCDs (30). Reducing the burden of NCDs will therefore require, in additional to better treatment and management of metabolic risks, population-based prevention actions focussed on improving diets and increasing physical activity levels (29).

Our study demonstrated that, over the last three decades, age-standardised metabolic risk factors have increased. Particularly, exposure to high BMI, FPG and LDL cholesterol levels have increasing over this period. Throughout this period of increasing metabolic risk exposure, however, age-standardised CVD deaths in Australia declined, which is in line with global trends toward decreasing age-standardised CVD deaths, as reported in GBD 2019 (14). The

For peer review only - http://bmjopen.bmj.com/site/about/guidelines.xhtml

justification for the apparent paradox may be the effect of access to care and great improvements in treatment, sociocultural factors, cohort effects, and other behavioural and environmental factors not recorded in the GBD study (32).

The findings revealed that in 2019, CVDs, neoplasm, and CKD are the leading causes of NCDs deaths attributable to metabolic risk, and CVD, diabetes mellitus and neoplasms are the leading causes of NCD DALYs attributable to metabolic risks. Of metabolic risk factors, BMI was accountable for a substantial proportion of deaths and DALYs due to diabetes, CKD and CVD. This is in line with other study findings that metabolic risks have been a significant contributor for high burden of NCD, especially for CVD and diabetes in Australia (5) and globally (28). Notably, overweight and obesity have been the main driver of mortality due to CVD, diabetes and chronic disease in Australia and the United States (33). Worldwide, high BMI has an increasing trend and was responsible for a high burden of CVD and diabetes mellitus (34, 35).

The result of this study shows that age-standardised death rates of CVD related to combined metabolic risks have significantly decreased while age-standardised death rates of neurological disease and CKD related to combined metabolic risks have increased. This is in line with the evidence that shows deaths of CVD have substantially declined in Australia, whilst the burden of neurological disorders has increased (5). In 2018, neoplasm (34%) and CVDs (21%) were the leading causes of death in Australia and high BMI and FPG were the main contributors to this burden (5).

The findings of this study provide the estimates of the level and trends of the metabolic risk-related NCD burden at the national level covering three-decades. The results underscore the importance of metabolic risks for the prevention of NCD burden. The prevention of NCDs requires a multifaceted strategy that targets the ‘causes of the causes’ of NCDs (23), and prioritizing prevention strategies for metabolic risk factors to improve population health. Reducing overweight in Australia needs to be comprehensive and wide-ranging, involving multi-sectoral collaboration and regulatory reforms (36). The most recent National Obesity Strategy, launched in Australia in 2022, focuses on the addressing the causes of obesity to reduce the population prevalence of obesity. Australian governments (States, Territories and Commonwealth) should urgently implement the strategy (37) to address the rising absolute burden of NCDs. Further study should assess the spatiotemporal pattern of NCD burdens attributed to metabolic risks incorporating the sociocultural contexts to inform practices and strategies at the lower local levels.

For peer review only - http://bmjopen.bmj.com/site/about/guidelines.xhtml

This study has strengths and limitations. One of the strengths of this study is that we used a longitudinal statistical method (Joinpoint regression analysis) to model the long-term trend of NCD burden attributed to metabolic risks. This modelling method helps to segment the long period into parts and identify the changing pattern of NCD burden for each period interval. Another strength of this study is the use of data from the GBD dataset, which is one of the most comprehensive and reliable data sources.

The limitations of this study include the likelihood that the metabolic risk related disease burden may vary between rural and urban areas, different regions or states, and socio-cultural conditions, which could not be characterised in this study. In GBD, the use of similar relative risks in certain age and gender groups in all countries may overestimate or underestimate the exact picture of disease burden, because metabolic risk factors may not influence disease outcomes in the same way in different population groups (4). Additionally, because the GBD study used secondary data, there might be a lack of metabolic risk data to estimate exposure levels, although this was addressed by the modelling methods using spatiotemporal Gaussian process regression (ST-GPR) and DisMod-meta-regression 2.1(4).

Conclusion

Despite a relative reduction in the metabolic risk-related burden of NCDs in Australia over the last three decades, metabolic risks continue to contribute significantly to the burden of NCDs, primarily CVD, neoplasms, diabetes mellitus and chronic kidney disease in the country. The absolute burden of NCDs also contributes to increase. To reduce the substantial burden of NCDs, multifaceted strategy and policies with multi-sectoral collaboration should focus on metabolic risks and other upstream NCD risk factors.

Acknowledgment: The authors would like to thank the Institute for Health Metrics and Evaluation for providing the data.

Author Contributions: Conceptualised and designed the study: KTK MN; analysed the data: KTK; Interpreted the results: KTK MN AP KB; drafted the manuscript: KTK and MN. Revised the manuscript critically for important intellectual content: KTK MN AP KB FT. All authors have read and approved the manuscript.

For peer review only

Funding: This research received no specific grant from any funding agency or the commercial or not-for-profit sectors. KTK is supported by Dean's Postdoctoral Fellowship at Deakin University (N/A). KB is supported by a Heart Foundation Future Leader Fellowship (102047). AP is supported by a National Health and Medical Research Council (NHMRC) Investigator Grant (N/A). MN is supported by an NHMRC Ideas Grant (GNT2002334). The contents of this publication are solely the responsibility of the authors and do not reflect the views of the NHMRC.

Conflicts of interest: Authors have no conflicting interests to declare.

Data sharing statement: All the data used in this study are publicly available online on the Institute of Health Metrics and Evaluation website (https://vizhub.healthdata.org/gbd-results/).

Patient consent for publication: Not applicable

Ethics approval: Not applicable. We used publicly available data.

References

1. World Health Organization 2018. Noncommunicable diseases country profiles. Geneva, Switzerland: WHO; 2018.
2. World Health Organization. Cardiovascular diseases (CVDs). [Fact sheet], WHO 2021; Available at: https://www.who.int/news-room/fact-sheets/detail/cardiovascular-diseases-(cvds). .
3. WHO. World health statistics 2020: monitoring health for the SDGs, sustainable development goals. 2020.
4. Murray CJL, Aravkin AY, Zheng P, Abbafati C, Abbas KM, Abbasi-Kangevari M, et al. Global burden of 87 risk factors in 204 countries and territories, 1990–2019: a systematic analysis for the Global Burden of Disease Study 2019. The Lancet. 2020;396(10258):1223-49.
5. Australian Institute of Health and Welfare. Australian Burden of Disease Study: Impact and causes of illness and death in Australia, 2018. Available at https://www.aihw.gov.au/. .
6. Australian Bureau of Statistics 2018. National Health Survey: first results, 2017–18. ABS cat. no. 4364.0.55.001. Canberra: ABS.
7. United Nations 2022. Sustainable development goals (SDGs). Avilable at https://www.un.org/sustainabledevelopment/health/.
8. WHO. Global action plan for the prevention and control of noncommunicable diseases 2013–2020. Geneva: World Health Organization. 2013.
9. Kontis V, Mathers CD, Rehm J, Stevens GA, Shield KD, Bonita R, et al. Contribution of six risk factors to achieving the 25×25 non-communicable disease mortality reduction target: a modelling study. The Lancet. 2014;384(9941):427-37.
10. Wijnen A, Bishop K, Joshy G, Zhang Y, Banks E, Paige E. Observed and predicted premature mortality in Australia due to non-communicable diseases: a population-based study examining progress towards the WHO 25X25 goal. BMC Med. 2022;20(1):57.
11. Australian Institute of Health and Welfare 2020. A framework for monitoring overweight and obesity in Australia. Cat. no. PHE 272. Canberra: AIHW.
12. Crosland P, Ananthapavan J, Davison J, Lambert M, Carter R. The health burden of preventable disease in Australia: a systematic review. Aust N Z J Public Health. 2019;43(2):163-70.

For peer review only - http://bmjopen.bmj.com/site/about/guidelines.xhtml

13. Begg SJ, Vos T, Barker B, Stanley L, Lopez AD. Burden of disease and injury in Australia in the new millennium: measuring health loss from diseases, injuries and risk factors MJA 2008;188:36–40.
14. Vos T, Lim SS, Abbafati C, Abbas KM, Abbasi M, Abbasifard M, et al. Global burden of 369 diseases and injuries in 204 countries and territories, 1990–2019: a systematic analysis for the Global Burden of Disease Study 2019. The Lancet. 2020;396(10258):1204-22.
15. Cole T, Bellizzi M, Flegal K, Dietz W. Establishing a standard definition for child overweight and obesity worldwide: international survey. BMJ. 2000;320:1240–3.
16. World Cancer Research Fund & American Institute for Cancer Researc. Food, nutrition, physical activity and the prevention of cancer: a global perspective. Washington, D.C. 2018.
17. Gefeller O. Theory and application of poulation attributable risk estimates in cross sectional studies. Statistical app. 1990;2(4):323-31.
18. Roth GA, Abate D, Abate KH, Abay SM, Abbafati C, Abbasi N, et al. Global, regional, and national age-sex-specific mortality for 282 causes of death in 195 countries and territories, 1980–2017: a systematic analysis for the Global Burden of Disease Study 2017. The Lancet. 2018;392(10159):1736-88.
19. National Cancer Institute. Joinpoint Trend Analysis Software, Version 4.9.1.0; Methodology and tools, surveillance research program, National Cancer Institute, 2022. 2022.
20. Kim H-J, FAY MP, Feuer EJ, Midthune DN. Permutation tests for joinpoint regression with applications to cancer rates. Statist Med. 2000; 19:335-51.
21. Clegg LX, Hankey BF, Tiwari R, Feuer EJ, Edwards BK. Estimating average annual per cent change in trend analysis. Stat Med. 2009;28(29):3670-82.
22. Australian Bureau of Statistics. Changing Patterns of Mortality in Australia, 1968 - 2017. . 2018.
23. Howse E, Rychetnik L, Marks L, Wilson A. What does the future hold for chronic disease prevention research? Aust N Z J Public Health. 2020;44(5):336-40.
24. Austr alian Health Minister s' Advisor y Council. National Strategic Framework for Chronic Conditions, 2017. Australian Government, Canberra. Available from: https://www.health.gov.au/resources/publications/national-strategic-framework-for-chronic-conditions
25. Australian Bureau of Statistics. Australian demographic statistics, 2019. Canberra: ABS; 2020.
26. Australian Institute of Health and Welfare. Risk factors contributing to chronic disease. Cat No. PHE 157. Canberra: AIHW. 2012.
27. Institute for Health Metrics and Evaluation (IHME). GBD Compare; 2022 Available at https://vizhubhealthdataorg/gbd-compare/
28. Peters R, Ee N, Peters J, Beckett N, Booth A, Rockwood K, et al. Common risk factors for major noncommunicable disease, a systematic overview of reviews and commentary: the implied potential for targeted risk reduction. Ther Adv Chronic Dis. 2019;10:2040622319880392.
29. Budreviciute A, Damiati S, Sabir DK, Onder K, Schuller-Goetzburg P, Plakys G, et al. Management and Prevention Strategies for Non-communicable Diseases (NCDs) and Their Risk Factors. Front Public Health. 2020;8:574111.
30. World Health Organization. Global health risks: mortality and burden of disease attributable to selected major risks. Geneva: WHO. 2009.
31. Taylor AW, Grande ED, Jing Wu1 ZS, Campostrini S. Ten-year trends in major lifestyle risk factors using an ongoing population surveillance system in Australia Population Health Metrics 2014;12(31).
32. Fullman N, Yearwood J, Abay SM, Abbafati C, Abd-Allah F, Abdela J, et al. Measuring performance on the Healthcare Access and Quality Index for 195 countries and territories and selected subnational locations: a systematic analysis from the Global Burden of Disease Study 2016. The Lancet. 2018;391(10136):2236-71.

For peer review only - http://bmjopen.bmj.com/site/about/guidelines.xhtml

33. Adair T, Lopez AD. The role of overweight and obesity in adverse cardiovascular disease mortality trends: an analysis of multiple cause of death data from Australia and the USA. BMC Med. 2020;18(1):199.
34. Lin X, Xu Y, Xu J, Pan X, Song X, Shan L, et al. Global burden of noncommunicable disease attributable to high body mass index in 195 countries and territories, 1990-2017. Endocrine. 2020;69(2):310-20.
35. Dai H, Alsalhe TA, Chalghaf N, Ricco M, Bragazzi NL, Wu J. The global burden of disease attributable to high body mass index in 195 countries and territories, 1990-2017: An analysis of the Global Burden of Disease Study. PLoS Med. 2020;17(7):e1003198.
36. Obesity policy coalition. Obesity in Australia: A decade of inaction, available at https://www.opc.org.au/. 2020.
37. Commonwealth of Australia. The National Obesity Strategy 2022-2032. Health Ministers Meeting. 2022.

For peer review only

List of figures

Figure 1. Trends of NCDs deaths and DALYs attributable to combined and individual metabolic risks: Age-standardized death rates (A); proportion of deaths (B); Age-standardized DALY rates (C) and proportions of DALYs (D) between 1990 and 2019 in Australia from Join point regression analysis. (Note: * indicates APC is significantly different from zero at the α = 0.05 level)

Figure 2. Age-specific rates and proportions of non-communicable disease deaths (A), and DALYs (B) attributable to metabolic risk factors by sex in Australia in 2019 (Proportions are of all NCD deaths/DALYs).

Figure 3. Trends of NCDs deaths and DALYs attributable to combined metabolic risks over time (1990 to 2019) by sex in Australia from Join point regression analysis: Age-standardized death rates (A); proportions of deaths (B); Age standardised DALY rates (C) proportion of DALYs (D) (Note: * indicates APC is significantly different from zero at the α = 0.05 levels)

For peer review only - http://bmjopen.bmj.com/site/about/guidelines.xhtml

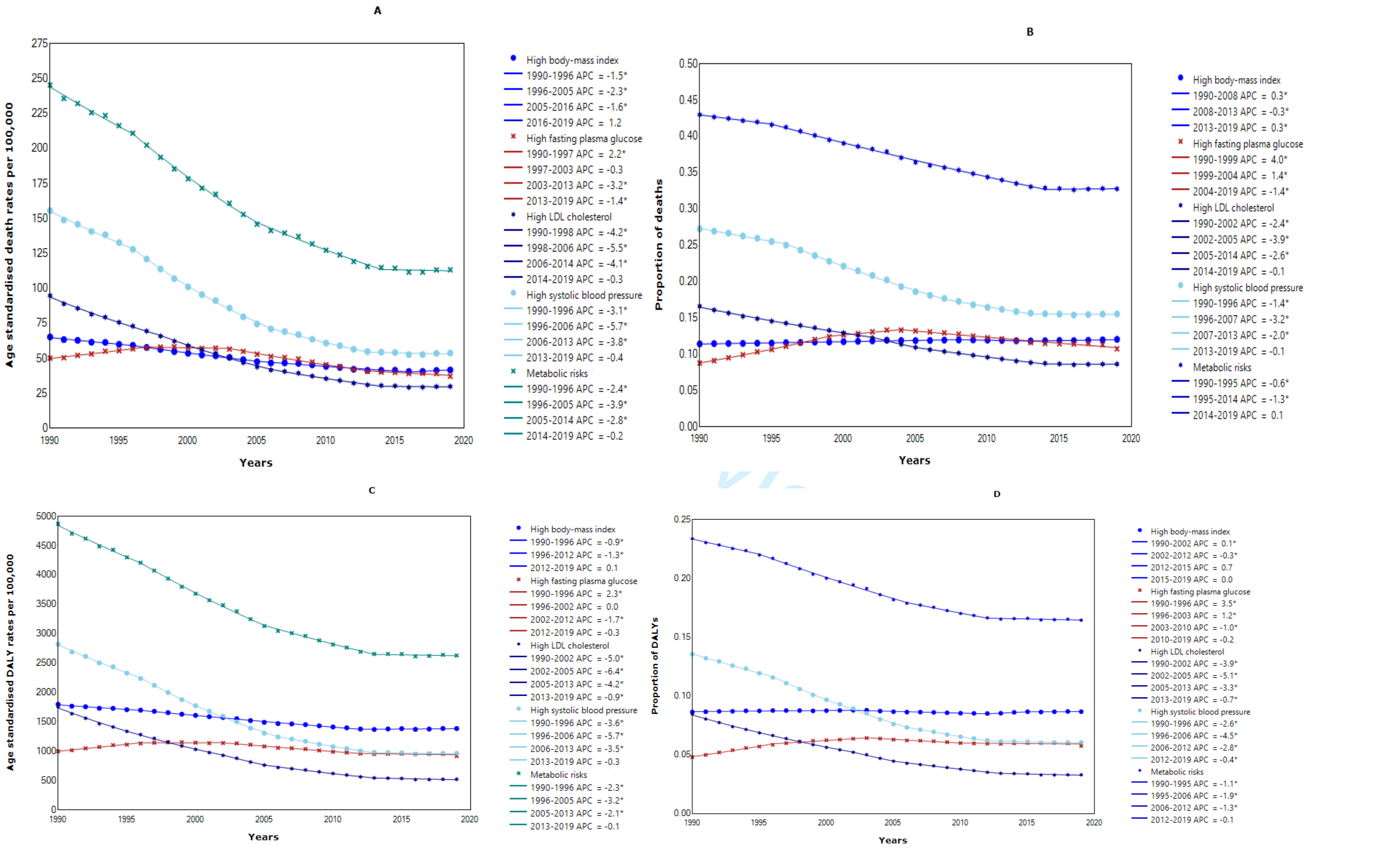

Figure 1. Trends of NCDs deaths and DALYs attributable to combined and individual metabolic risks: Age-standardized death rates (A); proportion of deaths (B); Age-standardized DALY rates (C) and proportions of DALYs (D) between 1990 and 2019 in Australia from Join point regression analysis. (Note: * indicates APC is significantly different from zero at the α = 0.05 level

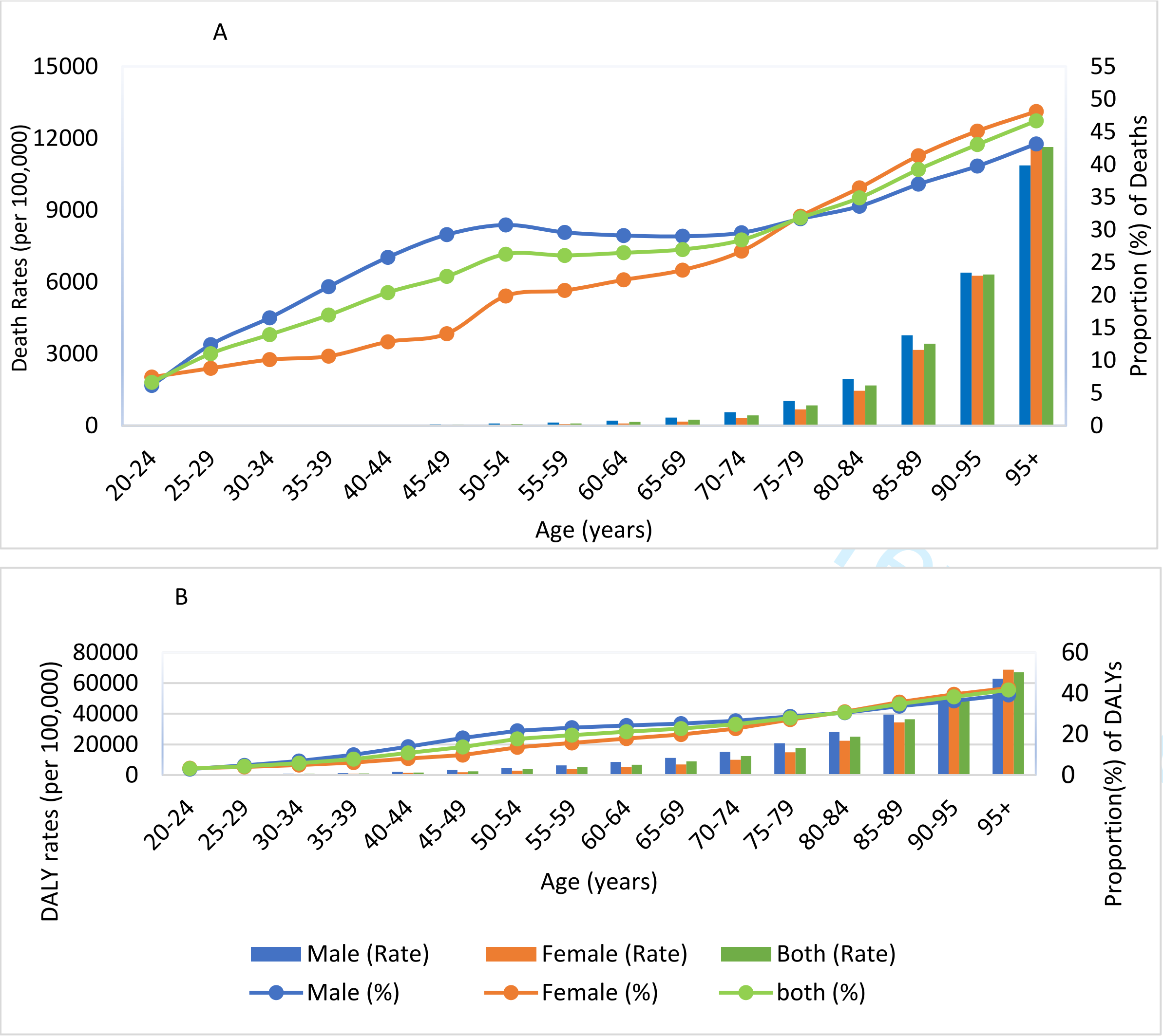

Figure 2. Age-specific rates and proportions of non-communicable disease deaths (A), and DALYs (B) attributable to metabolic risk factors by sex in Australia in 2019 (Proportions are of all NCD deaths/DALYs).

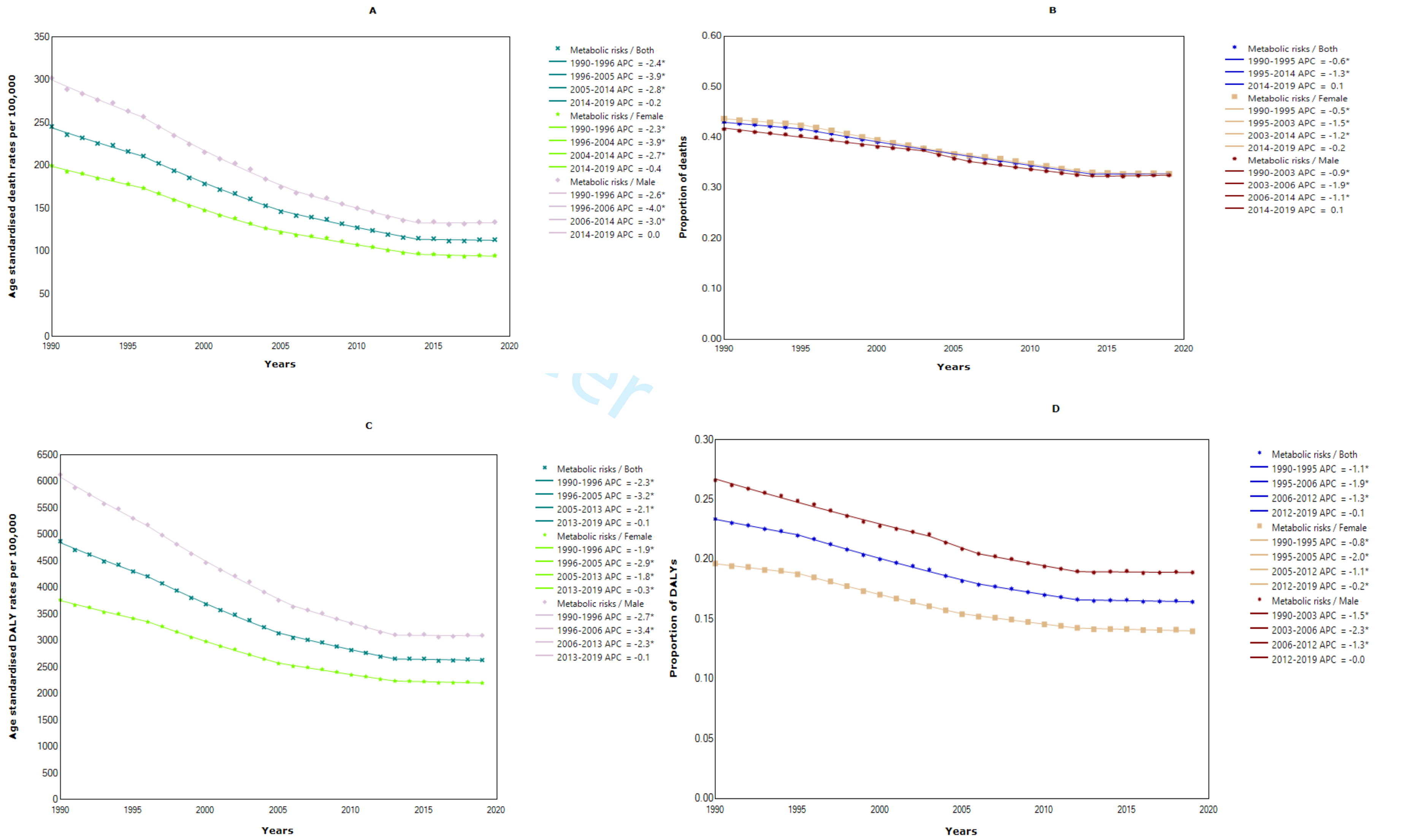

Figure 3. Trends of NCDs deaths and DALYs attributable to combined metabolic risks over time (1990 to 2019) by sex in Australia from Join point regression analysis: Age-standardized death rates (**A**); proportions of deaths **(B)**; Age standardised DALY rates **(C)** proportion of DALYs (**D**) (Note: * indicates APC is significantly different from zero at the α = 0.05 levels)

Trends and impacts of metabolic risk factors on non-communicable disease burdens in Australia, 1990–2019: Analysis of the Global Burden of Disease Study 2019

Kelemu Tilahun Kibret[1*], Kathryn Backholer[1] Anna Peeters[1] Fisaha Tesfay[1] Melanie Nichols[1]

[1]Deakin University, Global Centre for Preventive Health and Nutrition, Institute for Health Transformation, School of Health and Social Development, Faculty of Health, 1 Gheringhap Street, Geelong, VIC 3220, Australia.

*Corresponding Author: kelemu.kibret@deakin.edu.au; 221 Burwood Highway, Burwood VIC 3125, Australia

Supplementary Tables

Table 1 Age standardised rates of summary exposure value (SEV) of metabolic risk factors and average annual percentage change, 1990 - 2019

Risk factor	1990 SEV	2010 SEV	2019 SEV	AAPC 1990-2019	AAPC 2010-19
Metabolic risk factors (combined)	24.0 (19.1, 29.7)	30.8 (25.3, 36.7)	33.2 (27.4, 39.3)	1.1 (1.1, 1.1) *	0.8 (0.8,0.9) *
High systolic blood pressure	28.0 (26.1, 30.1)	21.8 (19.8, 23.9)	24.4 (20.7, 28.3)	-0.5 (-0.5, -0.4) *	1.2 (1.1,1.3) *
High body mass index	22.9 (16.7, 31.1)	33.4 (26.1, 41.5)	35.8 (27.9, 44.1)	1.5 (1.5, 1.6) *	0.8 (0.7,0.8) *
Fasting plasma glucose	4.56 (4.1, 5.1)	7.7 (6.7, 8.7)	8.3 (7.2, 9.6)	2.1 (1.6, 2.6) *	0.6 (0.5,1.7) *
High LDL	49.4 (46.5, 52.3)	47.3 (44.2, 50.3)	48.6 (45.5, 51.7)	0.0 (-0.1, 0.1)	0.4 (0.4,0.5) *

AAPC- average annual percentage change, SEV- summary exposure value, * indicates AAPC is significantly different from zero at the alpha = 0.05 level

For peer review only - http://bmjopen.bmj.com/site/about/guidelines.xhtml

Table 2. Burden of non-communicable diseases (deaths and DALYs) attributed to metabolic risk factors in Australia, 2019

Risk factors	Deaths (95%UI)			DALYS (95%UI)		
	Number	Proportion, %(95%UI)	ASR (per 100,000)	Number	Proportion, % (95%UI)	ASR (per 100,000)
Metabolic risk factors combined	53369 (46725,59687)	34.4(30.1, 38.5	113.33(99.49- 126.60)	1060720(911391, 1228621)	20.0(17.3, 22.8)	2630.33(246.30,3063.65)
HBP	25498 (20153,30851)	16.4 (12.9, 19.8)	53.8 (43.4, 64.4)	406445 (342906, 471339)	7.7 (6.3, 9.1)	966.5 (824.7, 1111.5)
BMI	18713 (11915,26149)	12.0 (7.7, 16.8)	41.7 (27.0, 57.5)	525135 (353676, 706414)	9.9 (6.8, 12.9)	1387.7 (954.66, 1855.0)
FPG	17640 (12073,25501)	11.4 (7.8, 16.4)	37.2 (25.7, 53.1)	381681 (286916, 499517)	7.2 (5.7, 9.1)	919.9 (698.2, 1187.7)
High LDL	14013 (9490,19216)	9.0 (6.1, 12.4)	30.0 (21.1, 40.10)	212393 (163123, 265910)	4.0 (3.0, 5.1)	525.2 (420.9, 639.0)

ASR- age standardised rate; DALY- disability adjusted life years; UI – uncertainty intervals

For peer review only

Table 3. Crude numbers, proportions of specific NCD deaths and DALYs attributable to metabolic risks in 1990 and 2019

Specific Causes of NCDs	Deaths attributable to combined metabolic risks n (95% UI)		DALYs attributable to combined metabolic risks n (95% UI)	
	1990	2019	1990	2019
All NCDs	45575 (49465, 41530)	53369 (59688, 46725)	935721 (846039, 1031253)	1060720 (911391, 1228621)
Neoplasms	2220 (1184, 3500)	5644 (3273, 8658)	47916 (26339, 73613)	110091 (65433, 164880)
Cardiovascular diseases	38412 (34749, 41552)	34921 (29085, 39517)	711313 (657997, 759437)	537319 (475508, 595062)
Chronic respiratory diseases	216 (122, 337)	160 (98, 236)	18915 (30673, 10535)	24758 (15038, 38755)
Neurological disorders	729 (118, 2228)	2954 (529, 8488)	11625 (3291, 29053)	41079 (12673, 98146)
Diabetes mellitus	2198 (2060, 2304.)	4239 (3726, 4629)	78885 (66264, 93765)	186528 (142543, 236662)
Chronic kidney disease	1712 (1557, 1827)	5207 (4293, 5893)	36869 (33602, 40498)	86550 (76642, 96199)
	Proportion % (95% UI)		Proportion % (95% UI)	
All NCDs	42.9 (39.0, 46.5)	34.4 (30.1, 38.5)	24.4 (2.3, 27.5)	20.0 (17.4, 22.8)
Neoplasms	7.1 (3.8, 11.0)	10.5 (6.1, 16.0)	6.4 (3.5, 9.8)	10.0 (5.9, 14.9)
Cardiovascular diseases	75.0 (69.5, 80.2)	67.7 (60.7, 73.7)	75.3 (70.7, 79.6)	68.3 (62.8, 73.3)
Chronic respiratory diseases	3.1 (1.8, 4.8)	1.4 (0.9, 2.1)	7.6 (4.4, 11.6)	7.5 (4.8, 11.1)
Neurological disorders	12.4 (4.0, 24.3)	17.5 (6.5, 30.4)	5.3 (1.8, 12.0)	9.8 (3.8, 19.4)
Diabetes mellitus	100.0 (100.0, 100.0)	100 (100.0,100.0)	100 (100.0, 100.0)	100 (100.0,100.0)
Chronic kidney disease	100 (100.0,100.0)	100 (100.0,100.0)	100 (100.0,100.0)	100 (100.0,100.0)

DALY- disability adjusted life years; NCDs – Non-communicable diseases; UI- uncertainty intervals

For peer review only

Supplementary Figures

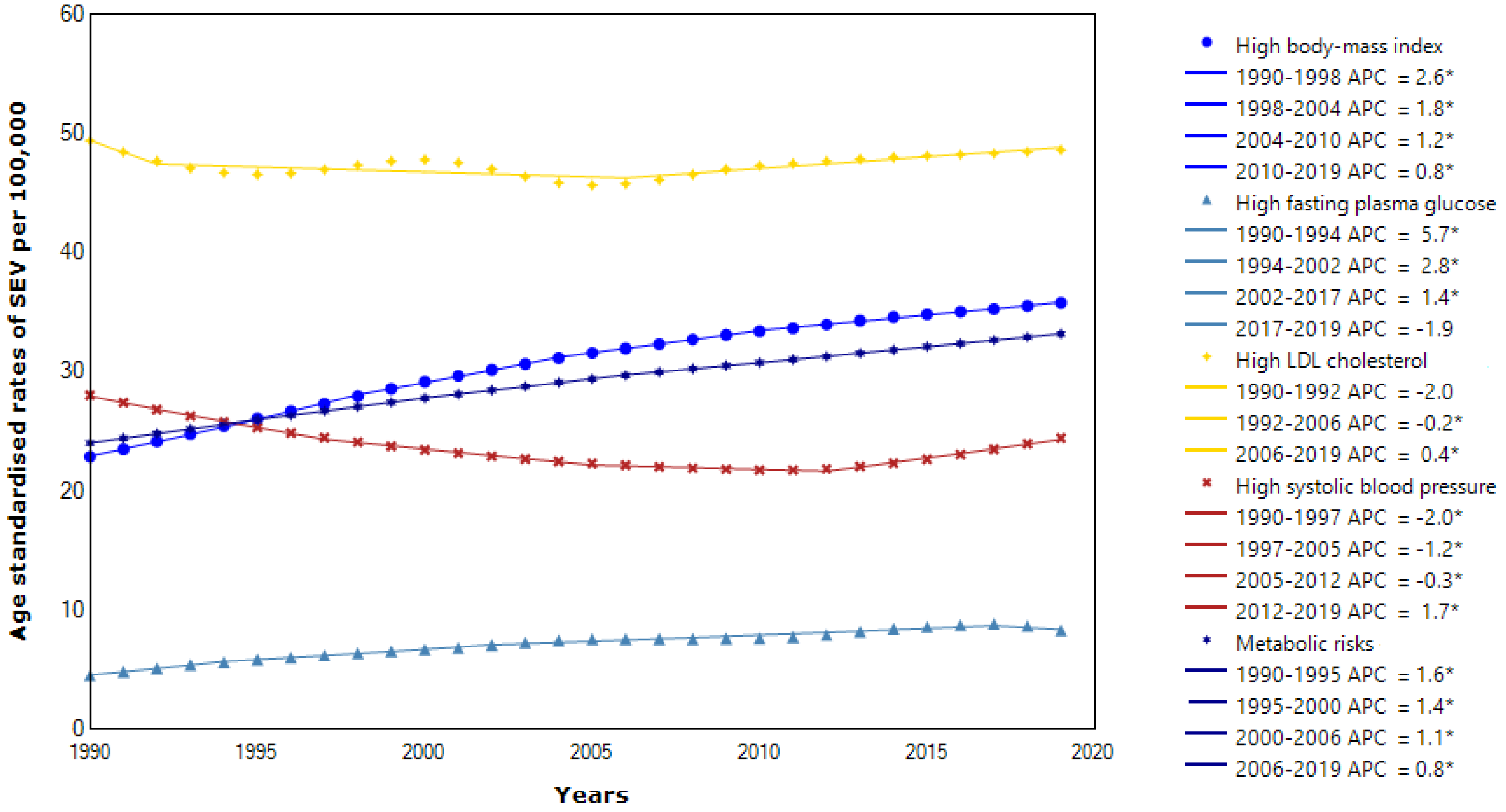

Supplementary Figure 1. Trends of SEV of combined and individual metabolic risks factors between 1990 and 2019, in Australia. Note: * indicates APC is significantly different from zero at the α = 0.05 level

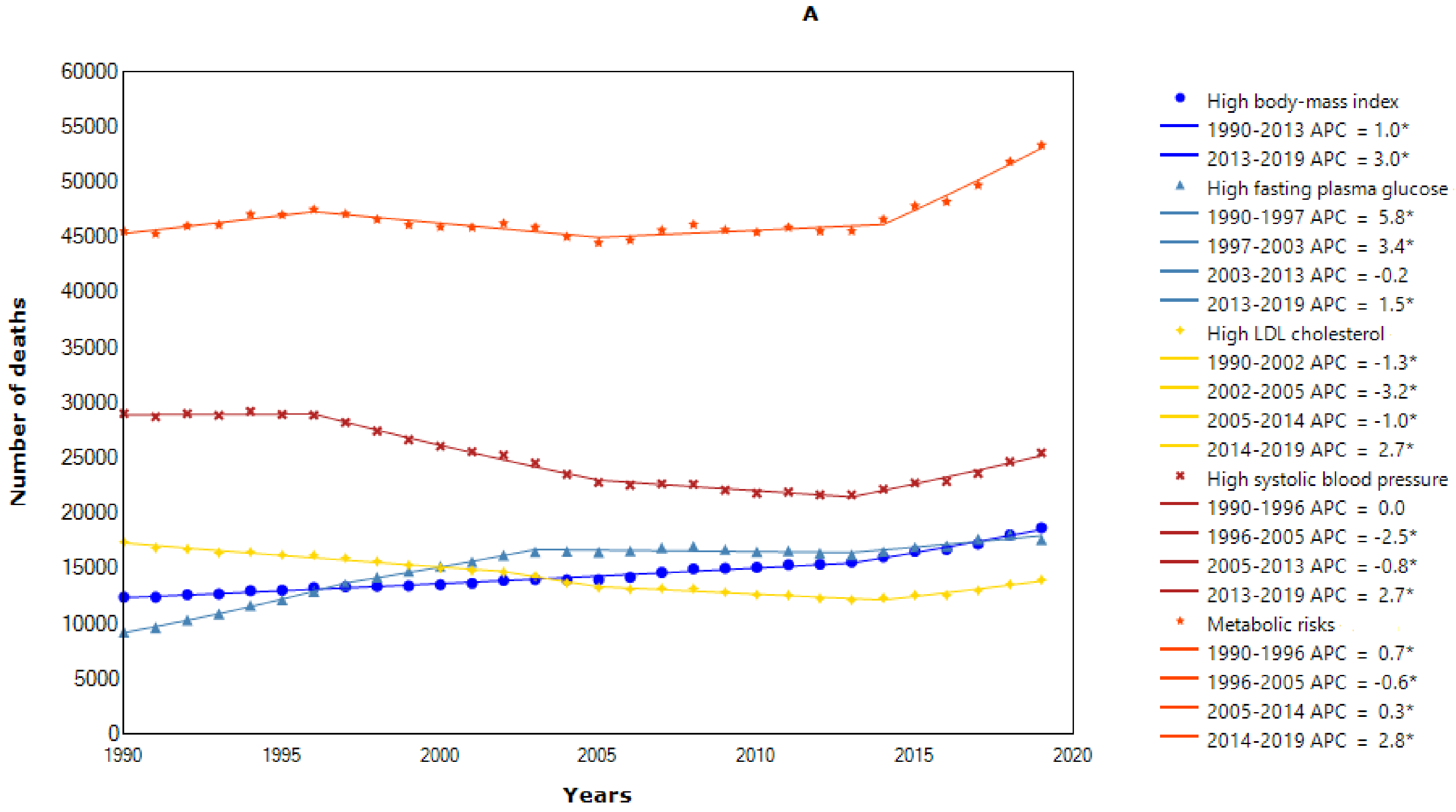
A
Number of deaths
60000
55000
50000
45000
40000
35000
30000
25000
20000
15000
10000
5000
0
1990
1995
2000
2005
2010
2015
2020
Years
High body-mass index
1990-2013 APC = 1.0*
2013-2019 APC = 3.0*
High fasting plasma glucose
1990-1997 APC = 5.8*
1997-2003 APC = 3.4*
2003-2013 APC = -0.2
2013-2019 APC = 1.5*
High LDL cholesterol
1990-2002 APC = -1.3*
2002-2005 APC = -3.2*
2005-2014 APC = -1.0*
2014-2019 APC = 2.7*
High systolic blood pressure
1990-1996 APC = 0.0
1996-2005 APC = -2.5*
2005-2013 APC = -0.8*
2013-2019 APC = 2.7*
Metabolic risks
1990-1996 APC = 0.7*
1996-2005 APC = -0.6*
2005-2014 APC = 0.3*
2014-2019 APC = 2.8*

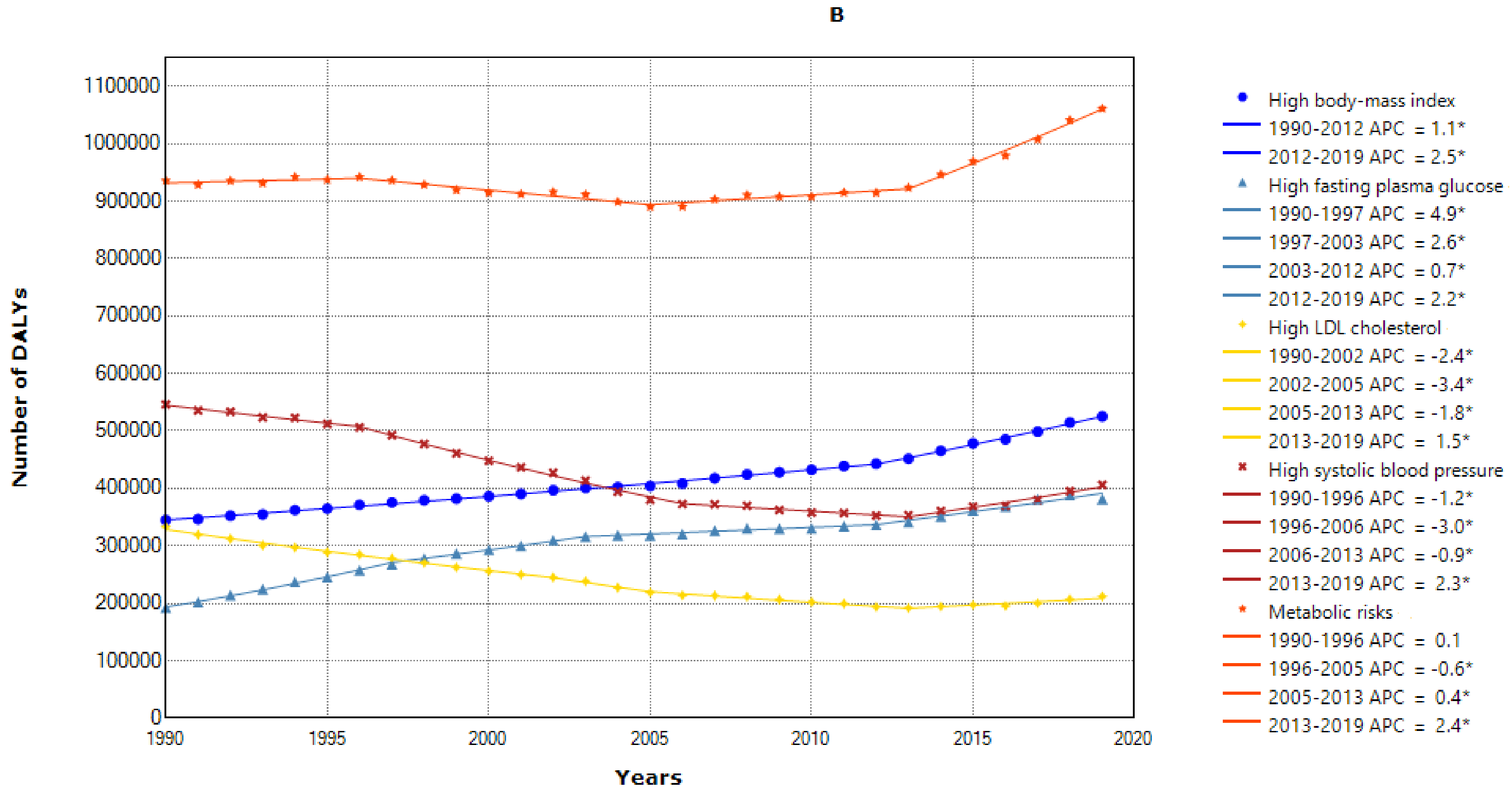

Supplementary Figures 2. Number of deaths (**A**) and DALYs (**B**) attributed to individual and combined metabolic risks overt time (1990-2019) in Australia from Join point regression analysis (Note: * indicates APC is significantly different from zero at the α = 0.05 levels).

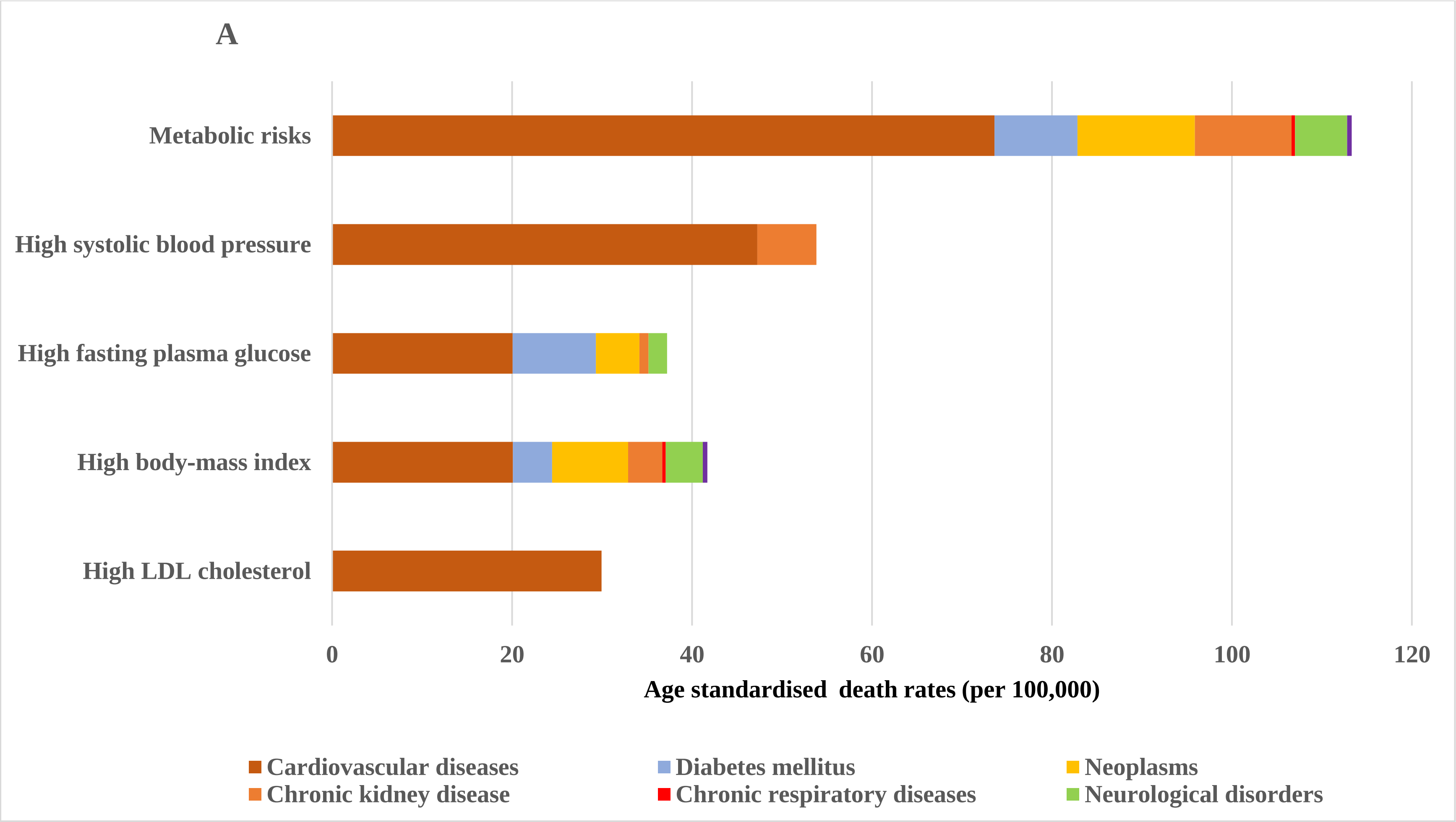
A
Metabolic risks
High systolic blood pressure
High fasting plasma glucose
High body-mass index
High LDL cholesterol
0
20
40
60
80
100
120
Age standardised death rates (per 100,000)
Cardiovascular diseases
Diabetes mellitus
Neoplasms
Chronic kidney disease
Chronic respiratory diseases
Neurological disorders

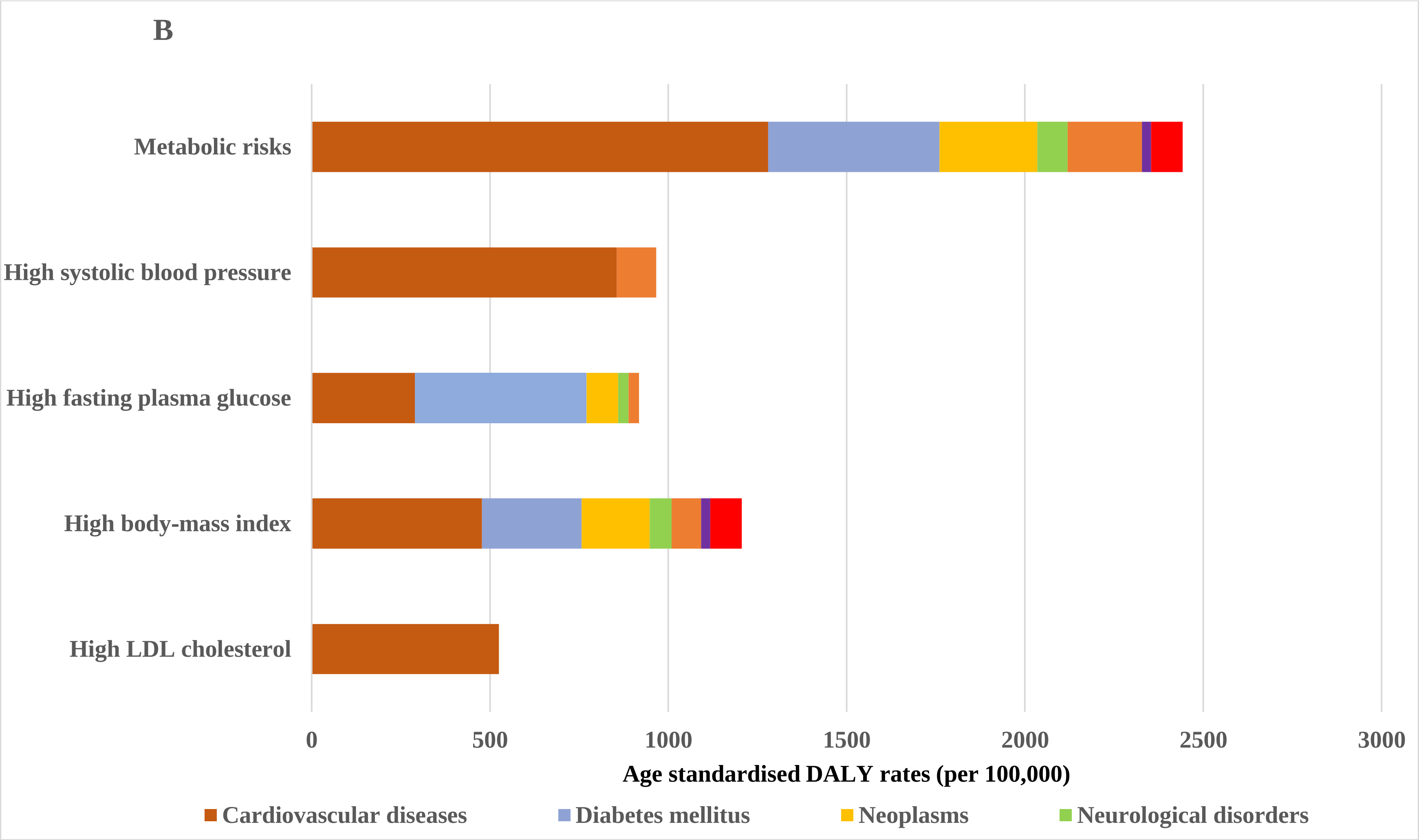

Supplementary Figure 3. Burden of specific NCDs attributable to individual and combined metabolic risks: age standardised death rates (A) and DALY rates (B) in Australia, 2019

STROBE 2007 (v4) checklist of items to be included in reports of observational studies in epidemiology*
Checklist for cohort, case-control, and cross-sectional studies (combined)

Section/Topic	Item #	Recommendation	Reported on page #
**Title and abstract**	1	(*a*) Indicate the study's design with a commonly used term in the title or the abstract	1
		(*b*) Provide in the abstract an informative and balanced summary of what was done and what was found	2
**Introduction**			
Background/rationale	2	Explain the scientific background and rationale for the investigation being reported	3-4
Objectives	3	State specific objectives, including any pre-specified hypotheses	4
**Methods**			
Study design	4	Present key elements of study design early in the paper	5
Setting	5	Describe the setting, locations, and relevant dates, including periods of recruitment, exposure, follow-up, and data collection	5
Participants	6	(*a*) *Cohort study*—Give the eligibility criteria, and the sources and methods of selection of participants. Describe methods of follow-up<br>*Case-control study*—Give the eligibility criteria, and the sources and methods of case ascertainment and control selection. Give the rationale for the choice of cases and controls<br>*Cross-sectional study*—Give the eligibility criteria, and the sources and methods of selection of participants	5
		(*b*) *Cohort study*—For matched studies, give matching criteria and number of exposed and unexposed<br>*Case-control study*—For matched studies, give matching criteria and the number of controls per case	
Variables	7	Clearly define all outcomes, exposures, predictors, potential confounders, and effect modifiers. Give diagnostic criteria, if applicable	5-6
Data sources/ measurement	8*	For each variable of interest, give sources of data and details of methods of assessment (measurement). Describe comparability of assessment methods if there is more than one group	*6*
Bias	9	Describe any efforts to address potential sources of bias	6
Study size	10	Explain how the study size was arrived at	7
Quantitative variables	11	Explain how quantitative variables were handled in the analyses. If applicable, describe which groupings were chosen and why	7
Statistical methods	12	(*a*) Describe all statistical methods, including those used to control for confounding	6-7
		(*b*) Describe any methods used to examine subgroups and interactions	
		(*c*) Explain how missing data were addressed	
		(*d*) *Cohort study*—If applicable, explain how loss to follow-up was addressed<br>*Case-control study*—If applicable, explain how matching of cases and controls was addressed	

For peer review only

		Cross-sectional study—If applicable, describe analytical methods taking account of sampling strategy	
		(*e*) Describe any sensitivity analyses	
**Results**			
Participants	13*	(a) Report numbers of individuals at each stage of study—eg numbers potentially eligible, examined for eligibility, confirmed eligible, included in the study, completing follow-up, and analysed	8
		(b) Give reasons for non-participation at each stage	
		(c) Consider use of a flow diagram	
Descriptive data	14*	(a) Give characteristics of study participants (eg demographic, clinical, social) and information on exposures and potential confounders	8
		(b) Indicate number of participants with missing data for each variable of interest	
		(c) *Cohort study*—Summarise follow-up time (eg, average and total amount)	
Outcome data	15*	*Cohort study*—Report numbers of outcome events or summary measures over time	9
		Case-control study—Report numbers in each exposure category, or summary measures of exposure	
		Cross-sectional study—Report numbers of outcome events or summary measures	
Main results	16	(*a*) Give unadjusted estimates and, if applicable, confounder-adjusted estimates and their precision (eg, 95% confidence interval). Make clear which confounders were adjusted for and why they were included	9-22
		(*b*) Report category boundaries when continuous variables were categorized	
		(*c*) If relevant, consider translating estimates of relative risk into absolute risk for a meaningful time period	
Other analyses	17	Report other analyses done—eg analyses of subgroups and interactions, and sensitivity analyses	
**Discussion**			
Key results	18	Summarise key results with reference to study objectives	23
Limitations	19	Discuss limitations of the study, taking into account sources of potential bias or imprecision. Discuss both direction and magnitude of any potential bias	26
Interpretation	20	Give a cautious overall interpretation of results considering objectives, limitations, multiplicity of analyses, results from similar studies, and other relevant evidence	23-25
Generalisability	21	Discuss the generalisability (external validity) of the study results	25
**Other information**			
Funding	22	Give the source of funding and the role of the funders for the present study and, if applicable, for the original study on which the present article is based	27

For peer review only

BMJ Open

The burdens of non-communicable disease attributable to metabolic risk factors in Australia, 1990–2019: Joinpoint Regression Analysis of the Global Burden of Disease Study

Journal:	*BMJ Open*
Manuscript ID	bmjopen-2022-071319.R1
Article Type:	Original research
Date Submitted by the Author:	30-May-2023
Complete List of Authors:	Kibret, Kelemu; Deakin University, Faculty of Health<br>Backholer, Kathryn; Deakin University, Faculty of Health<br>Peeters, A; Deakin University, Faculty of Health<br>Tesfay, Fisaha; Deakin University, Faculty of Health<br>Nichols, Melanie; Deakin University, Faculty of Health
**Primary Subject Heading**:	Public health
Secondary Subject Heading:	Epidemiology
Keywords:	CARDIOLOGY, EPIDEMIOLOGY, PUBLIC HEALTH

SCHOLARONE™
Manuscripts

I, the Submitting Author has the right to grant and does grant on behalf of all authors of the Work (as defined in the below author licence), an exclusive licence and/or a non-exclusive licence for contributions from authors who are: i) UK Crown employees; ii) where BMJ has agreed a CC-BY licence shall apply, and/or iii) in accordance with the terms applicable for US Federal Government officers or employees acting as part of their official duties; on a worldwide, perpetual, irrevocable, royalty-free basis to BMJ Publishing Group Ltd ("BMJ") its licensees and where the relevant Journal is co-owned by BMJ to the co-owners of the Journal, to publish the Work in this journal and any other BMJ products and to exploit all rights, as set out in our licence.

The Submitting Author accepts and understands that any supply made under these terms is made by BMJ to the Submitting Author unless you are acting as an employee on behalf of your employer or a postgraduate student of an affiliated institution which is paying any applicable article publishing charge ("APC") for Open Access articles. Where the Submitting Author wishes to make the Work available on an Open Access basis (and intends to pay the relevant APC), the terms of reuse of such Open Access shall be governed by a Creative Commons licence – details of these licences and which Creative Commons licence will apply to this Work are set out in our licence referred to above.

Other than as permitted in any relevant BMJ Author's Self Archiving Policies, I confirm this Work has not been accepted for publication elsewhere, is not being considered for publication elsewhere and does not duplicate material already published. I confirm all authors consent to publication of this Work and authorise the granting of this licence.

For peer review only

The burdens of non-communicable disease attributable to metabolic risk factors in Australia, 1990–2019: Joinpoint Regression Analysis of the Global Burden of Disease Study

Kelemu Tilahun Kibret[1*], Kathryn Backholer[1], Anna Peeters[1], Fisaha Tesfay[1], Melanie Nichols[1]

[1]Deakin University, Global Centre for Preventive Health and Nutrition, Institute for Health Transformation, School of Health and Social Development, Faculty of Health, 1 Gheringhap Street, Geelong, VIC 3220, Australia.

*Corresponding Author: kelemu.kibret@deakin.edu.au; 221 Burwood Highway, Burwood VIC 3125, Australia

For peer review only - http://bmjopen.bmj.com/site/about/guidelines.xhtml

Abstract

Background: Long-term and comparative assessments of trends in non-communicable disease (NCD) burden attributable to metabolic risk are sparse. This study aimed to assess burdens and trends of NCD attributable to metabolic risk factors in Australia, 1990 - 2019.

Design: Population-based observational study

Settings, and Data source: Data were extracted from the Global Burden of Disease (GBD) Study 2019 for Australia and trends in NCD burden attributable metabolic risks were estimated using the Joinpoint regression model.

Main Outcome Measures: NCD deaths and disability-adjusted life-years (DALYs) attributed to metabolic risk factors, 1990 -2019.

Results: Results indicate a 1.1% yearly increase in exposure to combined metabolic risk factors from 1990 to 2019. Between 1990 and 2019, the estimated absolute number of deaths from NCDs attributed to combined metabolic risks increased by 17%. However, metabolic risk-related NCD burdens in Australia decreased between 1990 and 2019. In 2019, 34% of NCD deaths and 20% of NCD DALYs were attributed to metabolic risk factors, compared to 42.9% and 24.4 % respectively in 1990. In 2019, cardiovascular diseases, neoplasms, and chronic kidney diseases were the most common NCD deaths attributed to metabolic risks. High body mass index accounted for the highest proportion of diabetes deaths (47%) and DALYs (58.1%) as well as chronic kidney disease deaths (35.4%) and DALYs (39.7%). Similarly, high systolic blood pressure contributed to a high proportion of chronic kidney disease deaths (60.9%) and DALYs (53.2%), and cardiovascular diseases (CVDs) deaths (44%) and DALYs (46%).

Conclusion: Whilst the contribution of metabolic risk factors to the burden of NCDs has declined from 1990 to 2019, their role in NCD death and disability remains a challenge as the prevalence of these risk factors has increased. Prevention strategies should focus on metabolic risks particularly high body mass index and high systolic blood pressure to substantially reduce NCD burdens.

Key words: trends noncommunicable diseases; metabolic risk factors; deaths; disability adjusted life years, Australia

For peer review only - http://bmjopen.bmj.com/site/about/guidelines.xhtml

Strength and limitation of this study

- Application of longitudinal statistical method (Joinpoint regression analysis) which help to model the long-term trend of NCD burden attributed to metabolic risks.
- The use of data from the GBD dataset, which is one of the most comprehensive and reliable data sources.
- The likelihood that the metabolic risk related disease burden may vary between rural and urban areas, different regions or states, and socio-cultural conditions, which could not be characterised in this study.
- In GBD, the use of similar relative risks in certain age and gender groups in all countries may overestimate or underestimate the exact picture of disease burden, because metabolic risk factors may not influence disease outcomes in the same way in different population groups.

Introduction

Currently, non-communicable diseases (NCDs) are the leading cause of mortality and morbidity worldwide, accounting for 71% of all deaths (1). In particular, cardiovascular diseases (CVDs) (e.g. ischemic heart disease and stroke) are leading causes of death in both high income and low and middle income countries (2, 3). Non-communicable diseases are multifactorial diseases caused by complex interactions of both behavioural and biological (metabolic) risk factors (4). Metabolic risk factors, including high systolic blood pressure (SBP), high body mass index (BMI) and high fasting plasma glucose (FPG) are among the top five leading risk factors (with smoking and unhealthy diet) for non-communicable diseases in the world (4). Globally, high SBP (SBP ≥ 130 mmHg) , high FPG (≥7 mmol/L), and high BMI (BMI ≥25 kg/m^2) were respectively responsible for 9.3%, 6.8% and 6.3% of disability-adjusted life years (DALYs) in 2019 (4). The largest proportion of disease burden in Australia in 2018 was attributed to metabolic risk factors - high body mass index (8.4%), high blood pressure (5.1%) and high blood plasma glucose (6.3%) (5).

Globally, the largest increases in the prevalence of modifiable risk factors from 2010 to 2019 were for metabolic risk factors such as high FPG and high BMI (4). According to the Australian National Health Survey 2017-18, the prevalence of high BMI (BMI ≥25 kg/m^2) was 67%, high blood pressure (SBP ≥ 130 mmHg or diastolic blood pressure (DBP) ≥ 90 mmHg was 22.8%,

For peer review only - http://bmjopen.bmj.com/site/about/guidelines.xhtml

high FPG was 5% in adults aged over 18 year (6). Further, 21% of individuals aged 65 years and over had high cholesterol level (≥240 mg/dL) (6).

NCDs have been recognised as a global priority in the United Nations' Sustainable Development Goals (SDG), which set a target of reducing premature death due to NCDs by one-third by 2030 (7). Similarly, the World Health Organization (WHO) has set a global target of a 25% reduction in premature death from CVDs, cancer, type 2 diabetes or chronic respiratory diseases by 2025, which can be achieved by reducing the burden of metabolic and behavioural risk factors using appropriate prevention approaches (8). A modelling study on the effect of risk factors on NCDs showed that premature mortality from the four main NCDs may be decreased worldwide by 22% in men and 19% in women if the SDG risk factor targets could be accomplished (9). A modelling study in Australia shows that a 25% relative reduction in premature deaths due to four NCDs in 2025, compared to 2010 levels, could be achieved if the age-standardised mortality rates continue to decrease by an average of 1.64% annually (10).

A clear understanding of the trends and impacts of metabolic risk factors for NCDs is necessary to prioritize prevention and control strategies to achieve NCD reduction goals. The evidence needs to be updated and tracked using the most recent available data (8, 11), and consistent, long-term and comparable findings at the national level are necessary. Assessing which risk factors notably increased or decreased over time and their impact on major NCDs is essential to prioritise and evaluate public health strategies and programmes at the regional or national level. Australia is a high-income country that has seen an overall decline in cardiovascular disease (CVD) mortality rates for some years now (12), but continues to experience a high prevalence of metabolic risk factors (5).). Australia's situation is not unique - many high-income countries also face similar challenges (13). For example, the United States, and many European countries also have high levels of obesity (14) and type 2 diabetes (15). Although some studies have examined the burden of diseases due to high BMI, blood glucose or cholesterol level in Australia covering short periods (5, 16, 17), long-term and comparative assessment of trends in NCD burden attributable to metabolic risk is still sparse. It is unclear which specific NCD burden attributed to metabolic risks has increased or decreased over time and for which metabolic risk factor. Thus, this study aims to investigate trends in the burden of NCDs attributable to metabolic risk factors in Australia between 1990 and 2019 using the latest Global Burden of Diseases (GBD) data. The objectives are to assess: 1) changes in exposure levels of metabolic risk factors in Australia between 1990 and 2019; 2) the burden of

For peer review only - http://bmjopen.bmj.com/site/about/guidelines.xhtml

NCDs (deaths and DALYs) attributable to metabolic risk factors in 2019; 3) temporal trends in the burden of NCDs attributable to metabolic risk from 1990 to 2019. This study provides context-specific insights into the burden of NCDs attributable to key metabolic risk factors in Australia. The study demonstrates the changing trends in this burden of NCDs attributable to metabolic risk factors over time. Our findings can inform the development of targeted prevention and management strategies for specific population groups, defined by high BMI and high blood pressure.

Methods

Patient and public involvement

Patients or members of the public were not involved in this study.

Study design and setting

This study is a Population based observational study with an in-depth analysis of the Global Burden of Diseases study 2019 data of Australia using a Joinpoint regression analysis.

Data sources

The GBD 2019 study data were used for this study (4, 18). The GBD study is an ongoing global collaborative epidemiological study that comprehensively assesses the burden of diseases, injuries, and risk factors for 204 countries and territories by sex and age group, and currently includes data from 1990 to 2019. The GBD provides estimates of deaths and DALYs due to 369 diseases and injuries, and 87 risk factors (4, 18). The GBD study results can be used by global, regional, national, and local policy and decision-makers to gain insights into the burden and trends of population health challenges and optimise responses.

The GBD study incorporates data from many various data sources, including censuses, household surveys, vital statistics, disease registries, disease notifications, health service use, government and international websites and other sources. All the data sources used in GBD are available on the Global Health Data Exchange (GHDx) website (https://ghdx.healthdata.org/gbd-2019/data-input-sources). The collated data are used to generate standard epidemiological estimates, including prevalence, mortality, and DALYs, as well as the attributable burdens (mortality and DALYs) for specific risk factors using a comparative risk assessment framework. Through this framework, GBD investigators: 1) identify evidence-based risk–outcome pairs; 2) determine the exposure level associated with

For peer review only - http://bmjopen.bmj.com/site/about/guidelines.xhtml

minimum risk, termed the theoretical minimum risk exposure level (TMREL); 3) estimate relative risks, population attributable fractions (PAFs) and attributable burdens (deaths and DALYs) for each risk factor-outcome pair. Detailed descriptions and methods used in GBD 2019 have been published elsewhere (4, 18). Brief descriptions of

Description of variables

Summary exposure value (SEV)

The GBD study estimated the exposure distributions for risk factors as a summary exposure value (SEV) (4). SEV is a relative risk-weighted prevalence of exposure, that measures the extent to which a population is exposed to a risk factor and the severity by which that risk contributes to the disease burden. SEV is reported on a scale of 0-100, where 0 means that no excess risk exists for a population, and 100 means that the population is at its highest risk. We assessed the combined metabolic risks (high BMI, high SBP, high FPG and LDL cholesterol), and each individual metabolic risk.

Theoretical minimum risk exposure level (TMREL)

For each risk factor-outcome pair, the GBD study defines what level of exposure to a risk factor leads to the lowest amount of disease (4). The theoretical minimum risk exposure level value (TMREL) is the level of risk exposure associated with the lowest risk (4). Detailed TMREL estimation for each risk factor was described elsewhere (4). In the GBD, the TMREL for 'high body-mass index' for adults (ages 20+) was defined as BMI 20 – 25kg/m^2, and for children based upon International Obesity Task Force standards (19). The TMREL level for FPG is defined as 4.8-5.4 mmol/L; and for low-density lipoprotein (LDL) cholesterol the TMREL defined as 0.7–1.3 mmol/L. The TMREL level for SBP is defined as 110–115 mmHg (4).

Estimation of effect size, population attributable fractions and attributable burdens

The GBD 2019 evaluated the strength of epidemiological evidence on the causal relationship between each metabolic risk factor and disease outcome pair, using the World Cancer Research Fund evidence appraisal tool (20). For those risk factor-outcome pairs graded as having convincing or probable evidence, relative risks were estimated using data from meta-analysis of cohort, case-control and/or intervention studies (4). The outcome-specific burden of disease attributable to risk factors was determined by estimating the population attributable fraction (PAF) (4). PAF is the proportion of the disease burden that would be reduced in a population if exposure to a risk factor was equivalent to the level of TMREL. The PAFs provide estimates of the proportion of cases that might be prevented if a particular risk factor were to be reduced

For peer review only - http://bmjopen.bmj.com/site/about/guidelines.xhtml

in the population. PAF incorporates the strength of association between risk factors and the outcome of interest, as well as the observed prevalence of risk factors in the population (21).

In the GBD study, the metrics used to quantify burden of disease were deaths and DALYs (3). DALYs are the sum of years of life lost and years lived with disability (18). For each risk-outcome pair, the attributable burden was estimated by multiplying the numbers of deaths or DALYs observed in the population by the PAF (4). In the GBD study, age-standardised rates (ASRs) (per 100,000) were estimated using the GBD world standard population, computed using population estimates for 2010–35 from the World Population Prospects (18, 22). In GBD study, the age standardised proportion is population attributable fraction (PAF) (4). For some diseases or outcomes in the GBD study, certain risk factors may have a PAF of 100%. The PAF can range from 0% to 100%, but a PAF of 100% does not necessarily mean that the risk factor or exposure is the sole cause of the health outcome or disease. For instance, age-standardised proportion of diabetes mellitus and chronic kidney disease deaths was 100% attributed to metabolic risks (high fasting plasma glucose and kidney dysfunction) (4). But it does not necessarily mean that the FPG and kidney dysfunction is the sole cause of diabetes and CKD respectively.

Data processing and statistical analysis

This study focused on the burden of NCDs attributable to individual metabolic risk factors (high BMI, high SBP, high FPG and high LDL cholesterol) and combined metabolic risks in Australia. We extracted numbers, proportions, and age-standardised rates (per 100,000) of metabolic risk-related deaths and DALYs and their 95% uncertainty intervals (UIs) of NCDs in Australia from 1990 to 2019 from the GBD study's GHDx results tool, (http://ghdx.healthdata.org/gbd-results-tool). We selected specific some NCDs (neoplasms, CVDs, diabetes mellitus, chronic kidney diseases (CKD), neurological disorders, chronic respiratory diseases (CRD)) that have summary estimates with common metabolic risk factors. We also extracted NCD deaths and DALYs attributable to metabolic risks by sex and age (20 years and over). Similarly, we extracted the age-standardised summary exposure values (SEV) for Australia for each metabolic risk factor from 1990 to 2019.

Using these extracted data from GBD results tool, the temporal trends in NCD burdens (deaths and DALYs) attributable to each metabolic risk factor over time (1990 to 2019) were assessed using the Joinpoint regression model (separately by sex) (23). This regression model

For peer review only

segments the entire period and identifies the time points (years) at which there has been a significant change in trends (Joinpoints) (24). Then, the annual percentage change (APC) with its 95% confidence interval (CI) of rates between these inflection points is estimated. The average annual percentage change (AAPC) with its 95% CI for the entire period (1990 -2019) and for the last decade (2010-2019) was also calculated, with respect to the underlying model fitted by the Joinpoint analysis. We examined the trends of the most recent decade for which data were available (2010-2019) to more closely examine the recent trends, which are of policy relevance, and because key policies and strategies related to NCDs have been published in the last decade, such as a Global Action Plan for the Prevention and Control of NCDs (8) and National Primary Health Care Strategy of Australia (25). AAPC is estimated as a weighted average of the APCs using the Joinpoint segment lengths as weights (26). The AAPC is a summary measure of the trend for the entire period. If there are no Joinpoints identified, the model produces only one APC, which is equal to the overall AAPC.

We used the Grid Search Modelling Method with the default recommended parameter values - the minimum number of observations at either end of Joinpoint is two, and the minimum number of measurements between two Joinpoints is two. We specified a minimum of zero and a maximum of four Joinpoints and a Monte Carlo Permutation method was used to test significant changes in trends over the specified period. The permutation test was also used to determine whether APCs and AAPCs differed from zero using $p < 0.05$ as statistically significant (24). Data extracted from the GBD were processed in an excel sheet, and the trend analysis was carried out using the Joinpoint Regression Program version 4.9.1.0 (23).

Results

Trends in risk exposure for metabolic risk factors in Australia from 1990 to 2019

Most age standardised SEV of individual metabolic risk factors showed an increasing trend; the exposure to combined metabolic risk factors increased by 1.1% per from 1990 to 2019, though the annual rate of increase was smaller in the most recent decade, at 0.8% from 2010 to 2019. Exposure to high BMI and high FPG showed a yearly increase of 1.5% and 2.0%, respectively, but high SBP decreased by 0.5% per year from 1990 to 2019, with a recent decline in the SEV of high FPG from 2017 and a rise in the SEV of high SBP from 2012 onwards. (Supplementary Table 1 and Supplementary Figure 1)

For peer review only - http://bmjopen.bmj.com/site/about/guidelines.xhtml

The burden of NCDs attributable to metabolic risk factors in Australia

In 2019, metabolic risks accounted for approximately 34% of all NCD deaths and 20% of NCD DALYs in Australia in 2019, with similar age-standardized proportions for both sexes, but a higher proportion of NCD DALYs were attributed to metabolic risks in males than in females. (Supplementary Table 2). The age-standardized death rates for NCDs linked to metabolic risks were 133.9 per 100,000 for males and 94.7 per 100,000 for females, while the metabolic risk-related age-standardized DALY rate was 3095.8 per 100,000 for males and 2198.7 per 100,000 for females (Supplementary Table 2). Age-specific rates and proportion of NCD deaths and DALYs related to metabolic risks increased with age in both sexes (Supplementary Figures 2A, 2B).

Of metabolic risk factors, high SBP and high BMI were the largest contributors to NCD deaths and DALYs, with high SBP estimated to contribute 16.4% of NCD deaths and 8% of NCD DALYs, while BMI was estimated to contribute to 12.0% of NCD deaths and 10% of NCD DALYs (Supplementary Table 3), followed by high FPG which was a significant contributor to NCD deaths (11.4%) and DALYs (7.2%), (Supplementary Table 3).

Temporal trends of metabolic risk related NCD burden from 1990 to 2019 in Australia

Between 1990 and 2019, the estimated absolute number of NCD deaths and DALYs attributed to combined metabolic risks increased by 17% and 13.4%, respectively (Supplementary Table 2 and Supplementary Figure 3). However, the ASRs of NCD deaths and DALYs attributable to metabolic risks roughly halved between 1990 and 2019, with AAPCs of -2.6% and -2.1% respectively (Table 1, Figure 1A and 1B).

The proportion of deaths an DALYs had a decreasing trend from 1990 to 2019 (Figure 1C and 1D). The proportions and ASRs of NCD deaths and DALYs related to metabolic risks have decreased for both males and females over time (1990 - 2019), with higher proportions of metabolic risk-related deaths in females but higher ASRs in males (Supplementary Figure 4A & 4B, Supplementary Table 2). The ASRs and proportions of metabolic risk related DALYs were also higher in men (Supplementary Table 2 & Supplementary Figure 4C & 4D).

Age-standardized NCD death rates attributed to high SBP (AAPC= -3.6%) and high LDL cholesterol (AAPC = -3.9%) showed a significant decrease, resulting in a total decline of 65.4%

For peer review only - http://bmjopen.bmj.com/site/about/guidelines.xhtml

and 68.4%, respectively from 1990 to 2019. Similarly, age standardized NCD DALY rates for high SBP and high LDL cholesterol decreased by an average of 3.6% and 4.1% per year, leading to a total reduction of 65.8% and 70.1% (Table 1).

For peer review only

For peer review only - http://bmjopen.bmj.com/site/about/guidelines.xhtml

1 Table 1. The age-standardised death rates and disability adjusted life years (DALYs) rates of non-communicable diseases (NCDs) attributable to
2 metabolic risks and their temporal trends between 1990 to 2019 by sex.

Risk factors	sex	Deaths per 100,000(95% UI)			DALYs per 100,000(95% UI)		
		1990	2019	AAPC (1990-2019)	1990	2019	AAPC (1990-2019)
Metabolic risk factors	Both	245.3 (221.7, 266.8)	113.3 (99.5, 126.6)	-2.6 (-2.8, -2.5) *	4870.9 (4402.1, 5377.5)	2630.3 (2246.3, 3063.7)	-2.1 (-2.2, -2.0) *
	Male	302.0 (274.7, 327.6)	133.9 (118.6, 149.9)	-2.8 (-2.9, -2.6) *	6124.6 (5580.6, 6704.6)	3095.8 (2692.8, 3578.5	-2.3 (-2.4, -2.2) *
	Female	199.8 (177.6, 219.0)	94.7 (81.5, 107.4)	-2.6 (-2.7, -2.4) *	3764.7 (3346.0, 4212.5)	2198.7 (1827.9, 2623.3)	-1.8 (-1.9, -1.7) *
High systolic blood pressure	Both	155.6 (131.2, 177.0)	53.8 (43.4, 64.4)	-3.6 (-3.8, -3.5) *	2822.6 (2506.5, 3140.8)	966.5 (824.7, 1111.5)	-3.6(-3.8, -3.5) *
	Male	67.9 (47.5, 98.8)	47.9 (33.0, 67.8)	-3.7 (-3.9, -3.5) *	1292.4 (1005.1, 1698.2)	1138.0 (878.9, 1451.8)	-3.7 (-3.9, -3.5) *
	Female	39.1 (27.6, 55.8)	28.3 (18.7, 42.)	-3.6 (-3.8, -3.4) *	784.2 (606.7, 1006.7	725.1 (538.8, 961.3)	-3.5 (-3.7, -3.4) *
High? Body	Both	65.3 (38.2, 95.6)	41.7 (27.0, 57.5)	-1.5 (-1.7, -1.3) *	1801.0 (1102.2, 2556.7)	1387.7 (954.7, 1855.0)	-0.9 (-0.9, -0.8) *

For peer review only - http://bmjopen.bmj.com/site/about/guidelines.xhtml

For peer review only

mass index	Male	75.6 (42.8, 113.0)	45.9 (27.9, 65.7)	-1.7 (-1.9, -1.6) *	2091.4 (1252.7, 3014.0)	1519.1 (1008.5, 2063.2)	-1.1 (-1.1, -1.0) *
	Female	55.1 (33.1, 81.4)	36.9 (24.4, 50.7)	-1.4 (-1.6, -1.1) *	1511.5 (947.8, 2146.6)	1255.7 (874.5, 1670.6)	-0.6 (-0.7, -0.6) *
High fasting plasma glucose	Both	50.2 (35.9, 71.0)	37.2 (25.7, 53.1)	-0.9 (-1.2, -0.7) *	1001.0 (782.8, 1275.1)	919.9 (698.2, 1187.71)	-0.2 (-0.4, -0.1) *
	Male	67.9 (47.5, 98.8)	47.9 (33.0, 67.8)	-1.2 (-1.3, -1.0) *	1292.4 (1005.1, 1698.2)	1138.1 (878.9, 1451.9)	-0.4 (-0.5, -0.2) *
	Female	39.1 (27.6, 55.8)	28.3 (18.7, 42.1)	-1.0 (-1.3, -0.7) *	784.2 (606.7, 1006.7)	725.1 (538.8, 961.3)	-0.3 (-0.5, -0.0) *
High LDL cholesterol	Both	94.9 (70.4, 122.1)	30.0 (21.1, 40.1)	-3.9 (-4.1, -3.7) *	1758.0 (1443.6, 2100.7)	525.2 (420.9, 639.0)	-4.1 (-4.4, -3.8) *
	Male	124.9 (95.7, 157.5)	38.3 (27.9, 49.9)	-4.0 (-4.2, -3.8) *	2467.1 (2060.7, 2905.8)	732.6 (601.9, 876.7)	-4.1 (-4.4, -3.8) *
	Female	70.4 (49.1, 94.7)	22.5 (14.6, 31.7)	-3.9 (-4.1, -3.6) *	1116.1 (855.4, 1393.3)	331.9 (249.8, 430.0)	-4.1 (-4.3, -3.9) *

LDL- low-density lipoprotein, AAPC – Average annual percentage change, *AAPC is significantly different from zero at the alpha = 0.05 level

For peer review only

For peer review only - http://bmjopen.bmj.com/site/about/guidelines.xhtml

Specific type of NCD deaths and DALYs attributable to metabolic risk factors

In 2019, cardiovascular disease deaths (34,921) and DALYs (537319) attributed to metabolic risks were the highest, followed by neoplasms with 5644 deaths and 110,091 DALYs and chronic kidney diseases with 5207 deaths and 86,550 DALYs. (Supplementary Table 4). The age-standardised proportion of diabetes mellitus and chronic kidney disease deaths was 100% attributed to metabolic risks in Australia. Similarly, a high proportion of age-standardised death rates of CVDs (68%), neurological disorders (16.6%) and neoplasms (10.3%) were attributed to combined metabolic risks (Table 2).

Each metabolic risk factor was responsible for a sizeable proportion of age-standardised death and DALY rates of specific NCDs (Table 2 and Supplementary Figure 5). About 47.0% deaths and 58.1% DALYs of diabetes mellitus, and about 35.4% deaths and 39.7% DALYs of CKD were attributed to high BMI. Similarly, 18.61% of CVD deaths and 25.5% of CVD DALYs were attributable to high BMI (Table 2). Furthermore, about 18.6% of deaths and 15.5% of DALY for CVDs related to high FPG (Table 2). High SBP was responsible for 60.9% deaths and 53.2% DALYs of CKD, and 44% deaths and 46% DALYs of CVD (Table 2).

For peer review only - http://bmjopen.bmj.com/site/about/guidelines.xhtml

Table 2. Burden of specific NCDs (deaths and DALYs) attributable to metabolic risks in Australia, 2019.

Specific Causes of NCDs	Deaths		DALYs	
	Proportion, % (95% UI)	ASRs per 100,000(95% UI)	Proportion, % (95% UI)	ASR per 100,000(95% UI)
**Combined metabolic risks**				
Neoplasms	10.3 (6.0, 15.61)	13.0 (7.5, 19.9)	9.5 (5.7, 14.1)	274.2 (164.4, 407.6)
Cardiovascular diseases	68.1 (61.6, 73.8)	73.6 (62.2, 82.8)	68.3 (63.0, 72.9)	1279.8 (1145.9, 1410.1)
Chronic respiratory diseases	1.6 (1.0, 2.3)	0.4 (0.2, 0.6)	9.3 (6.1, 13.1)	88.5 (54.1, 138.7)
Neurological disorders	16.6 (5.9, 29.2)	5.8 (1.0, 16.6)	7.02 (2.5, 14.8)	85.1 (26.4, 202.8)
Diabetes mellitus	100.0 (100.0, 100.0)	9.2 (8.2, 10.0)	100.0 (100.0, 100.0)	480.8 (365.6, 613.0)
Chronic kidney disease	100.0 (100.0, 100.0)	10.8 (9.0, 12.1)	100.0 (100.0, 100.0)	208.5 (184.8, 233.2)
**High body mass index**				
Neoplasms	6.7 (4.1, 9.5)	8.4 (5.2, 12.0)	6.6 (4.1, 9.2)	190.3 (118.8, 265.9)
Cardiovascular diseases	18.6 (11.8, 26.1)	20.1 (12.7, 28.4)	25.5 (17.6, 33.7)	477.8 (327.8, 641.5)
Chronic respiratory diseases	1.6 (1.0, 2.3)	0.4 (0.2, 0.6)	9.3 (6.12, 13.1)	88.5 (54.1, 138.7)
Neurological disorders	11.8 (3.8, 23.2)	4.1 (0.7, 12.2)	5.1 (1.5, 11.4)	61.6 (17.4, 152.5)
Diabetes mellitus	47.0 (31.9, 62.2)	4.3 (2.9, 5.9)	58.1 (44.9, 68.6)	279.5 (192.1, 383.4)
Chronic kidney disease	35.4 (16.5, 55.5)	3.8 (1.8, 6.0)	39.7 (25.4, 54.5)	82.7 (52.6, 116.2)

For peer review only - http://bmjopen.bmj.com/site/about/guidelines.xhtml

**High fast plasma glucose**				
Neoplasms	3.8 (1.0, 7.9)	4.8 (1.3, 9.9)	31 (0.8, 6.4)	89.1 (23.9, 184.0)
Cardiovascular diseases	18.6 (11.0, 31.0)	20.1 (11.8, 33.4)	15.5 (10.1, 23.8)	289.8 (186.2, 455.0)
Neurological disorders	5.9 (1.1, 14.3)	2.1(0.2, 7.8)	2.4(0.4, 6.7)	29.1 (5.1, 95.7)
Diabetes mellitus	100.0 (100.0, 100.0)	9.2 (8.2, 10.0)	100.0 (100.0,100.0)	480.8 (365.6, 613.0)
Chronic kidney disease	9.5 (6.6, 13.6)	1.0 (0.7, 1.5)	13.9 (11.1, 17.0)	29.1 (22.1, 37.3)
**High Systolic blood pressure**				
Cardiovascular diseases	43.7 (36.2, 51.6)	47.3 (37.5, 57.3)	45.7 (39.6, 51.3)	855.6 (720.6, 989.9)
Chronic kidney disease	60.9 (52.7, 68.2)	6.5 (5.3, 7.7)	53.2 (46.0, 59.7)	110.9 (92.3, 131.4)
**High LDL cholesterol**				
Cardiovascular diseases	27.71 (20.0, 36.3)	30.0 (21.1, 40.1)	28.0 (23.0, 33.9)	525.2 (420.9, 639.0)

ASR- Age-standardized rate; DALYs- Disability adjusted life years; NCDs – Non-communicable diseases, UI- uncertainty interval

For peer review only

For peer review only - http://bmjopen.bmj.com/site/about/guidelines.xhtml

Trends of specific NCD deaths and DALYs attributable to metabolic risks

Between 1990 and 2019, the metabolic risk-related age-standardised death rates of CVDs, chronic respiratory diseases and diabetes mellitus significantly declined by an average 3.5%, 3.7% and 0.8% per year (Supplementary Table 5). In contrast, age-standardised death rates of neoplasms, neurological disorders and chronic kidney disease attributed to metabolic risks significantly increased by 0.5%, 1.0% and 0.5% per year, respectively (Supplementary Table 5). Specifically, age-standardised death rates of chronic kidney disease related to BMI and FPG significantly increased by 1.1% and 2.0% per year, and death rates of neurological disorders linked to BMI and FPG increased by 1.0% and 0.9% per year. In contrast, age-standardised CVD death rates attributable to high BMI, high FPG, high SBP and high LDL cholesterol decreased by 2.8%, 1.5%, 3.9%, and 3.9% per year, respectively, between 1990 and 2019 (Supplementary Table 5).

The metabolic risk-related age-standardised DALY rates for CVD and chronic respiratory diseases significantly decreased by 3.6% and 0.6% per year between 1990 and 2019, whereas metabolic risk-related DALY rates of neoplasms, neurological disorders, diabetes mellitus and chronic kidney disease increased by 0.4%, 1.0%, 0.6% and 0.3% per year, respectively. However, the decline of metabolic risk-related CVD DALY rates between 2010 and 2019 (1.7% per year) were slow compared to over the last 30 years (3.6% per year) (Supplementary Table 6).

Similarly, age-standardised DALY rates of CVD attributable to High BMI, high FPG, high SPB and high LDL cholesterol declined by 2.7%, 1.5%, 3.9% and 4.1% per year between 1990 and 2019. But between 2010 and 2019, the rate of decline has slowed with an average annual rate of change of 1.2%, 1.9%, 1.6%, and 2.0% for high BMI, high FPG, high SPB and high LDL cholesterol, respectively (Supplementary Table 6).

Discussion

We assessed the trends and impact of metabolic risk factors on the burden of NCDs in the Australian population across age and sex between 1990 and 2019. Although the relative burden of metabolic risk related NCDs in Australia has declined between 1990 and 2019, metabolic risks continue to account for a significant proportion, and absolute number, of the NCD burden. Approximately one-third (34%) of NCD deaths and one-fifth (20%) of NCD DALYs were

For peer review only

attributed to metabolic risk factors in 2019. Moreover, most cardiovascular disease deaths and deaths from type 2 diabetes and chronic kidney diseases were attributed to metabolic risks.

The age-standardised rate of deaths and DALYs attributed to metabolic risks decreased between 1990 and 2019, although the absolute number of metabolic risk-related deaths and DALYs have increased. The reductions in age standardised NCD burdens attributed to metabolic risks could be because of enhancement in prevention programs, and the availability of effective treatments (27-29). In addition, the increase in NCD burden due to increased prevalence of BMI and FPG could offset by reduced risks from SBP, and LDL. The rise in the absolute number of metabolic risk-related NCD burdens could be due to the growing population size and aging population in Australia and advances in diagnostic methods that help diagnose the disease at its early stages (30). The incidence of many NCDs increases with age and many older individuals now live with chronic health conditions (6). In addition to the metabolic risk factors, other non-metabolic factors (16) might contribute to the NCDs burden in Australia. For instance, dietary risks and tobacco significantly contributed for the burden of NCD in 2018 and are risk factors themselves for the metabolic risks we examined in this study (5).

We show that NCD deaths and DALYs related to metabolic risks differed substantially by sex. The burden of NCDs attributed to metabolic risks was higher for males than for females and decreased overtime for both sexes at different rate. This might be due to the difference in risk factor distributions among genders (e.g. proportion of overweight is higher in males (75%) than females (60%), and males had higher smoking rates (16.5%) than females (11%) (6).

High BMI, SBP and FPG were responsible for high proportions of metabolic risk attributable NCD burden in 2019, each accountable for 10-16% of age standardised NCD deaths, and 5-8% of NCD DALYs. This is consistent with the results of the studies that show a high burden of NCD deaths was associated with high BMI and high SBP in Australian population in 2018 (5, 16). Metabolic risk factors are one of the health risk factors targeted for NCD prevention in the current Australian NCD National Framework (29). Metabolic risk factors along with other behavioural risk factors are important in Australia: nearly 90% of individuals over 45 years of age had at least one risk factor, and two-thirds of them had over three risk factors in 2012 (31). The prevalence of high BMI, SBP and FPG was also high in the Australian population in 2017/18 (6).

For peer review only - http://bmjopen.bmj.com/site/about/guidelines.xhtml

Our analysis of 2019 GBD data showed that high SBP, BMI, high FPG and high LDL cholesterol were ranked first, third (behind smoking), fourth and fifth among the top five leading risk factors of NCD deaths in Australia, respectively (32). BMI (first), SBP (third, behind smoking) and FPG (fourth) were the leading risk factors for NCD DALYs in the country (32). This metabolic risk related NCD burden is evident worldwide (33, 34), with evidence showing that a considerable proportion of NCD deaths is attributed to high blood pressure (13%), high blood sugar (6%), and overweight (5%) (35).

Our study showed that the proportion of the age standardised NCD death rates related to BMI and FPG slightly increased, while the proportion of NCDs attributable to SBP and LDL decreased, between 1990 to 2019. In line with these findings, BMI and FPG were the significant contributors to NCD deaths in Australia in 2018 (5, 16). The recent Australian Health Survey also revealed that the prevalence of high BMI has increased from 63% in 2014-15 to 67% in 2017-18 in the adult population (6). The other study findings also show that the proportions of individuals with obesity, high cholesterol, and high blood glucose increased between the year 2004 to 2013 (36). Sub-optimal dietary intake and low physical activity levels are intermediary risk factors between the metabolic risks and NCDs (35). Reducing the burden of NCDs will therefore require, in additional to better treatment and management of metabolic risks, population-based prevention actions focussed on improving diets and increasing physical activity levels (34).

Our study demonstrated that, between 1990 and 2019, age-standardised metabolic risk factors have increased. Particularly, exposure to high BMI, FPG and LDL cholesterol levels have increasing over this period. Throughout this period of increasing metabolic risk exposure, however, age-standardised CVD deaths in Australia declined, which is in line with global trends toward decreasing age-standardised CVD deaths, as reported in GBD 2019 (18). The justification for the apparent paradox may be the effect of access to care and great improvements in treatment, sociocultural factors, cohort effects, and other behavioural and environmental factors not recorded in the GBD study (37).

The findings revealed that in 2019, CVDs, neoplasm, and CKD are the leading causes of NCDs deaths attributable to metabolic risk, and CVD, diabetes mellitus and neoplasms are the leading causes of NCD DALYs attributable to metabolic risks. Of metabolic risk factors, BMI was accountable for a substantial proportion of deaths and DALYs due to diabetes, CKD and CVD. This is in line with other study findings that metabolic risks have been a significant contributor

For peer review only - http://bmjopen.bmj.com/site/about/guidelines.xhtml

for high burden of NCD, especially for CVD and diabetes in Australia (5) and globally (33). Notably, overweight and obesity have been the main driver of mortality due to CVD, diabetes and chronic disease in Australia and the United States (38). Worldwide, high BMI has an increasing trend and was responsible for a high burden of CVD and diabetes mellitus (39, 40).

The result of this study shows that age-standardised death rates of CVD related to combined metabolic risks have significantly decreased while age-standardised death rates of neurological disease and CKD related to combined metabolic risks have increased. This is in line with the evidence that shows deaths of CVD have substantially declined in Australia, whilst the burden of neurological disorders has increased (5). In 2018, neoplasm (34%) and CVDs (21%) were the leading causes of death in Australia and high BMI and FPG were the main contributors to this burden (5). In addition, CVD deaths and DALYs attributed to metabolic risks decreased significantly (3.5% and 3.6% per year, respectively) between 1990 and 2019, but the rate of decline slowed in the last decade (2010–2019) (1.9% and 1.7% per year). In Australia, there has been a recent increase in the prevalence of metabolic risk factors such as obesity and diabetes (6), which may slow the decline in CVD deaths and DALYs. In addition, the ageing of the Australian population may also partly explain the slower decline in CVD deaths and DALYs over the past decade (30).

The findings of this study provide the estimates of the level and trends of the metabolic risk-related NCD burden at the national level covering three-decades. The results underscore the importance of metabolic risks for the prevention of NCD burden. The prevention of NCDs requires a multifaceted strategy that targets the 'causes of the causes' of NCDs (28), and prioritizing prevention strategies for metabolic risk factors to improve population health. Reducing overweight in Australia needs to be comprehensive and wide-ranging, involving multi-sectoral collaboration and regulatory reforms (41). The most recent National Obesity Strategy, launched in Australia in 2022, focuses on the addressing the causes of obesity to reduce the population prevalence of obesity. Australian governments (States, Territories and Commonwealth) should urgently implement the strategy (42) to address the rising absolute burden of NCDs. Further study should assess the spatiotemporal pattern of NCD burdens attributed to metabolic risks incorporating the sociocultural contexts to inform practices and strategies at the lower local levels.

This study has strengths and limitations. One of the strengths of this study is that we used a longitudinal statistical method (Joinpoint regression analysis) to model the long-term trend of

For peer review only - http://bmjopen.bmj.com/site/about/guidelines.xhtml

NCD burden attributed to metabolic risks. This modelling method helps to segment the long period (1990 -2019) into parts and identify the changing pattern of NCD burden for each period interval. Another strength of this study is the use of data from the GBD dataset, which is one of the most comprehensive and reliable data sources.

The limitations of this study include the likelihood that the metabolic risk related disease burden may vary between rural and urban areas, different regions or states, and socio-cultural conditions, which could not be characterised in this study. In GBD, the use of similar relative risks in certain age and gender groups in all countries may overestimate or underestimate the exact picture of disease burden, because metabolic risk factors may not influence disease outcomes in the same way in different population groups (4). Additionally, because the GBD study used secondary data, there might be a lack of metabolic risk data to estimate exposure levels, although this was addressed by the modelling methods using spatiotemporal Gaussian process regression (ST-GPR) and DisMod-meta-regression 2.1(4).

Conclusion

Despite a relative reduction in the metabolic risk-related burden of NCDs in Australia between 1990 and 2019, metabolic risks continue to contribute significantly to the burden of NCDs, primarily CVD, neoplasms, diabetes mellitus and chronic kidney disease in the country. The absolute burden of NCDs also contributes to increase. This study adds to the growing body of evidence that metabolic risk factors are important contributors to the burden of NCDs in Australia and underscores the need for effective prevention and management strategies to address these risk factors. To reduce the substantial burden of NCDs, multifaceted strategy and policies with multi-sectoral collaboration should focus on metabolic risks and other upstream NCD risk factors.

Acknowledgment: The authors would like to thank the Institute for Health Metrics and Evaluation for providing the data.

Author Contributions: Conceptualised and designed the study: KTK MN; analysed the data: KTK; Interpreted the results: KTK MN AP KB; drafted the manuscript: KTK and MN. Revised the manuscript critically for important intellectual content: KTK MN AP KB FT. All authors have read and approved the manuscript.

Funding: This research received no specific grant from any funding agency or the commercial or not-for-profit sectors. KTK is supported by Dean's Postdoctoral Fellowship at Deakin University (N/A). KB is supported by a Heart Foundation Future Leader Fellowship (102047). AP is supported by a National Health and Medical Research Council (NHMRC) Investigator

For peer review only

Grant (N/A). MN is supported by an NHMRC Ideas Grant (GNT2002334). The contents of this publication are solely the responsibility of the authors and do not reflect the views of the NHMRC.

Conflicts of interest: Authors have no conflicting interests to declare.

Data sharing statement: All relevant data for this study are presented in the manuscript and provided as supplementary file.

Patient consent for publication: Not applicable

Ethics approval: Not applicable. We used publicly available data.

For peer review only

References

1. World Health Organization 2018. Noncommunicable diseases country profiles. Geneva, Switzerland: WHO; 2018.
2. World Health Organization. Cardiovascular diseases (CVDs). [Fact sheet], WHO 2021; Available at: https://www.who.int/news-room/fact-sheets/detail/cardiovascular-diseases-(cvds). .
3. WHO. World health statistics 2020: monitoring health for the SDGs, sustainable development goals. 2020.
4. Murray CJL, Aravkin AY, Zheng P, Abbafati C, Abbas KM, Abbasi-Kangevari M, et al. Global burden of 87 risk factors in 204 countries and territories, 1990–2019: a systematic analysis for the Global Burden of Disease Study 2019. The Lancet. 2020;396(10258):1223-49.
5. Australian Institute of Health and Welfare. Australian Burden of Disease Study: Impact and causes of illness and death in Australia, 2018. Available at https://www.aihw.gov.au/. .
6. Australian Bureau of Statistics 2018. National Health Survey: first results, 2017–18. ABS cat. no. 4364.0.55.001. Canberra: ABS.
7. United Nations 2022. Sustainable development goals (SDGs). Avilable at https://www.un.org/sustainabledevelopment/health/.
8. WHO. Global action plan for the prevention and control of noncommunicable diseases 2013–2020. Geneva: World Health Organization. 2013.
9. Kontis V, Mathers CD, Rehm J, Stevens GA, Shield KD, Bonita R, et al. Contribution of six risk factors to achieving the 25×25 non-communicable disease mortality reduction target: a modelling study. The Lancet. 2014;384(9941):427-37.
10. Wijnen A, Bishop K, Joshy G, Zhang Y, Banks E, Paige E. Observed and predicted premature mortality in Australia due to non-communicable diseases: a population-based study examining progress towards the WHO 25X25 goal. BMC Med. 2022;20(1):57.
11. Australian Institute of Health and Welfare 2020. A framework for monitoring overweight and obesity in Australia. Cat. no. PHE 272. Canberra: AIHW.
12. Australian Institute of Health and Welfare 2017. Trends in cardiovascular deaths. Avilable at https://www.aihw.gov.au/getmedia/2ba74f7f-d812-4539-a006-ca39b34d8120/aihw-21213.pdf.

For peer review only - http://bmjopen.bmj.com/site/about/guidelines.xhtml

13. Roth GA, Mensah GA, Johnson CO, Addolorato G, Ammirati E, Baddour LM, et al. Global Burden of Cardiovascular Diseases and Risk Factors, 1990-2019: Update From the GBD 2019 Study. J Am Coll Cardiol. 2020;76(25):2982-3021.
14. Hruby A, Hu FB. The Epidemiology of Obesity: A Big Picture. Pharmacoeconomics. 2015;33(7):673-89.
15. Goodall R, Alazawi A, Hughes W, Bravis V, Salciccioli JD, Marshall DC, et al. Trends in type 2 diabetes mellitus disease burden in European Union countries between 1990 and 2019. Sci Rep. 2021;11(1):15356.
16. Crosland P, Ananthapavan J, Davison J, Lambert M, Carter R. The health burden of preventable disease in Australia: a systematic review. Aust N Z J Public Health. 2019;43(2):163-70.
17. Begg SJ, Vos T, Barker B, Stanley L, Lopez AD. Burden of disease and injury in Australia in the new millennium: measuring health loss from diseases, injuries and risk factors MJA 2008;188:36–40.
18. Vos T, Lim SS, Abbafati C, Abbas KM, Abbasi M, Abbasifard M, et al. Global burden of 369 diseases and injuries in 204 countries and territories, 1990–2019: a systematic analysis for the Global Burden of Disease Study 2019. The Lancet. 2020;396(10258):1204-22.
19. Cole T, Bellizzi M, Flegal K, Dietz W. Establishing a standard definition for child overweight and obesity worldwide: international survey. BMJ. 2000;320:1240–3.
20. World Cancer Research Fund & American Institute for Cancer Researc. Food, nutrition, physical activity and the prevention of cancer: a global perspective. Washington, D.C. 2018.
21. Gefeller O. Theory and application of poulation attributable risk estimates in cross sectional studies. Statistical app. 1990;2(4):323-31.
22. Roth GA, Abate D, Abate KH, Abay SM, Abbafati C, Abbasi N, et al. Global, regional, and national age-sex-specific mortality for 282 causes of death in 195 countries and territories, 1980–2017: a systematic analysis for the Global Burden of Disease Study 2017. The Lancet. 2018;392(10159):1736-88.
23. National Cancer Institute. Joinpoint Trend Analysis Software, Version 4.9.1.0; Methodology and tools, surveillance research program, National Cancer Institute, 2022. 2022.
24. Kim H-J, FAY MP, Feuer EJ, Midthune DN. Permutation tests for joinpoint regression with applications to cancer rates. Statist Med. 2000; 19:335-51.
25. Australian Govermnent Department of Health and Aging. Building a 21st Century Primary Health Care System, Australia's First National Primary Health Care Strategy. Avaiulable at https://wwwnationalplanningcyclesorg/sites/default/files/country_docs/Australia/6552_nphc_1205pdf. 2010.
26. Clegg LX, Hankey BF, Tiwari R, Feuer EJ, Edwards BK. Estimating average annual per cent change in trend analysis. Stat Med. 2009;28(29):3670-82.
27. Australian Bureau of Statistics. Changing Patterns of Mortality in Australia, 1968 - 2017. . 2018.
28. Howse E, Rychetnik L, Marks L, Wilson A. What does the future hold for chronic disease prevention research? Aust N Z J Public Health. 2020;44(5):336-40.
29. Austr alian Health Minister s' Advisor y Council. National Strategic Framework for Chronic Conditions, 2017. Australian Government, Canberra. Available from: https://www.health.gov.au/resources/publications/national-strategic-framework-for-chronic-conditions

For peer review only - http://bmjopen.bmj.com/site/about/guidelines.xhtml

30. Australian Bureau of Statistics. Australian demographic statistics, 2019. Canberra: ABS; 2020.
31. Australian Institute of Health and Welfare. Risk factors contributing to chronic disease. Cat No. PHE 157. Canberra: AIHW. 2012.
32. Institute for Health Metrics and Evaluation (IHME). GBD Compare; 2022 Available at https://vizhubhealthdataorg/gbd-compare/
33. Peters R, Ee N, Peters J, Beckett N, Booth A, Rockwood K, et al. Common risk factors for major noncommunicable disease, a systematic overview of reviews and commentary: the implied potential for targeted risk reduction. Ther Adv Chronic Dis. 2019;10:2040622319880392.
34. Budreviciute A, Damiati S, Sabir DK, Onder K, Schuller-Goetzburg P, Plakys G, et al. Management and Prevention Strategies for Non-communicable Diseases (NCDs) and Their Risk Factors. Front Public Health. 2020;8:574111.
35. World Health Organization. Global health risks: mortality and burden of disease attributable to selected major risks. Geneva: WHO. 2009.
36. Taylor AW, Grande ED, Jing Wu1 ZS, Campostrini S. Ten-year trends in major lifestyle risk factors using an ongoing population surveillance system in Australia Population Health Metrics 2014;12(31).
37. Fullman N, Yearwood J, Abay SM, Abbafati C, Abd-Allah F, Abdela J, et al. Measuring performance on the Healthcare Access and Quality Index for 195 countries and territories and selected subnational locations: a systematic analysis from the Global Burden of Disease Study 2016. The Lancet. 2018;391(10136):2236-71.
38. Adair T, Lopez AD. The role of overweight and obesity in adverse cardiovascular disease mortality trends: an analysis of multiple cause of death data from Australia and the USA. BMC Med. 2020;18(1):199.
39. Lin X, Xu Y, Xu J, Pan X, Song X, Shan L, et al. Global burden of noncommunicable disease attributable to high body mass index in 195 countries and territories, 1990-2017. Endocrine. 2020;69(2):310-20.
40. Dai H, Alsalhe TA, Chalghaf N, Ricco M, Bragazzi NL, Wu J. The global burden of disease attributable to high body mass index in 195 countries and territories, 1990-2017: An analysis of the Global Burden of Disease Study. PLoS Med. 2020;17(7):e1003198.
41. Obesity policy coalition. Obesity in Australia: A decade of inaction, available at https://www.opc.org.au/. 2020.
42. Commonwealth of Australia. The National Obesity Strategy 2022-2032. Health Ministers Meeting. 2022.

List of figures

Figure 1. Trends of NCDs deaths and DALYs attributable to combined and individual metabolic risks: Age-standardized death rates (A); proportion of deaths (B); Age-standardized DALY rates (C) and proportions of DALYs (D) between 1990 and 2019 in Australia from Join point regression analysis. (Note: * indicates APC is significantly different from zero at the α = 0.05 level)

For peer review only - http://bmjopen.bmj.com/site/about/guidelines.xhtml

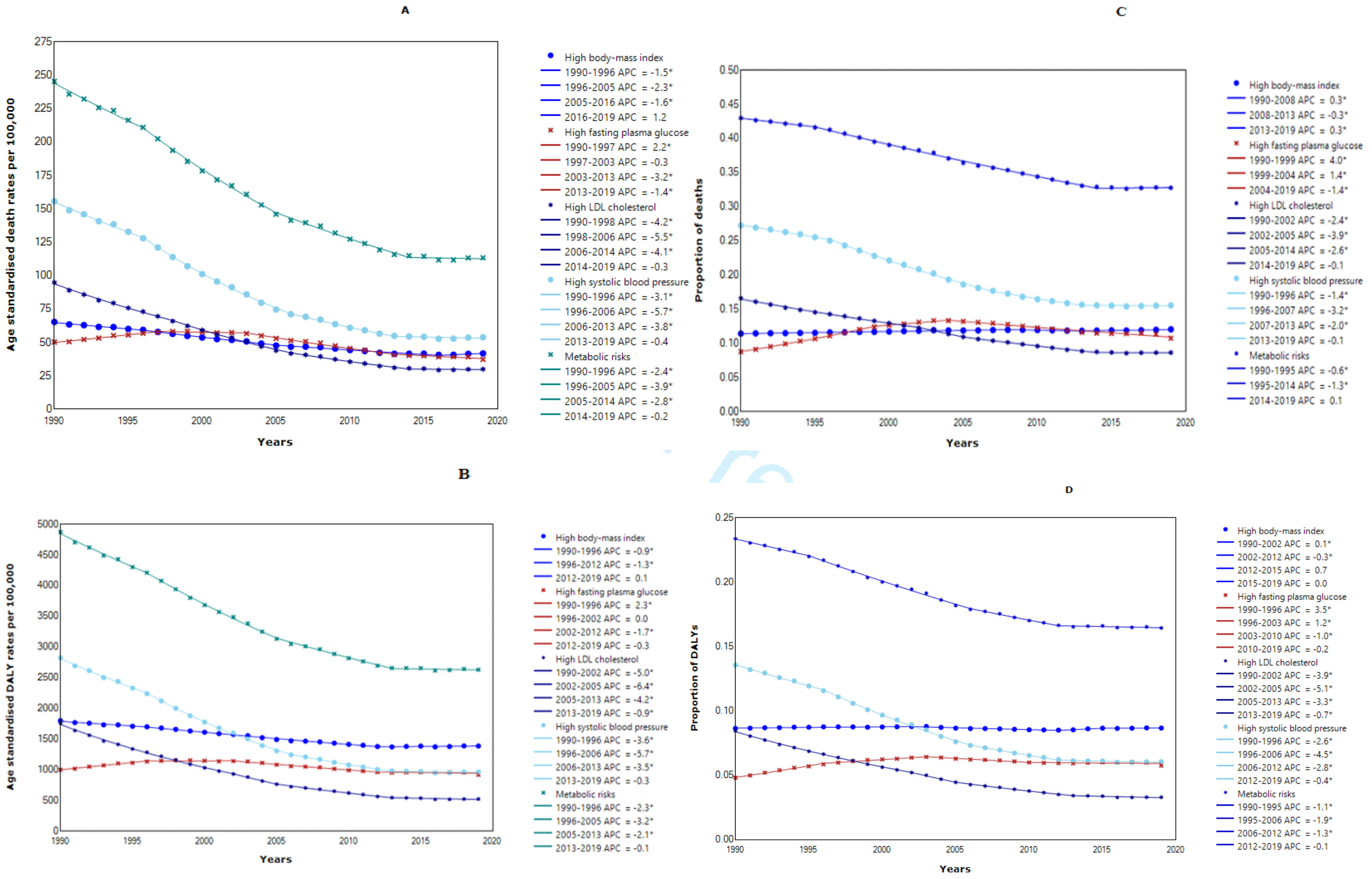

Figure 1. Trends of NCDs deaths and DALYs attributable to combined and individual metabolic risks: Age-standardized death rates (A); Age-standardized DALY rates (B) proportion of deaths (C); and proportions of DALYs (D) between 1990 and 2019 in Australia from Join point regression analysis. (Note: * indicates APC is significantly different from zero at the α = 0.05 level

The burdens of non-communicable disease attributable to metabolic risk factors in Australia, 1990–2019: Joinpoint Regression Analysis of the Global Burden of Disease Study

Kelemu Tilahun Kibret[1*], Kathryn Backholer[1] Anna Peeters[1] Fisaha Tesfay[1] Melanie Nichols[1]

[1]Deakin University, Global Centre for Preventive Health and Nutrition, Institute for Health Transformation, School of Health and Social Development, Faculty of Health, 1 Gheringhap Street, Geelong, VIC 3220, Australia.

*Corresponding Author: kelemu.kibret@deakin.edu.au

Supplementary Tables

Supplementary Table 1 Age standardised rates of summary exposure value (SEV) of metabolic risk factors and average annual percentage change, 1990 - 2019

Risk factor	1990 SEV	2010 SEV	2019 SEV	AAPC 1990-2019	AAPC 2010-19
Metabolic risk factors (combined)	24.0 (19.1, 29.7)	30.8 (25.3, 36.7)	33.2 (27.4, 39.3)	1.1 (1.1, 1.1) *	0.8 (0.8,0.9) *
High systolic blood pressure	28.0 (26.1, 30.1)	21.8 (19.8, 23.9)	24.4 (20.7, 28.3)	-0.5 (-0.5, -0.4) *	1.2 (1.1,1.3) *
High body mass index	22.9 (16.7, 31.1)	33.4 (26.1, 41.5)	35.8 (27.9, 44.1)	1.5 (1.5, 1.6) *	0.8 (0.7,0.8) *
Fasting plasma glucose	4.56 (4.1, 5.1)	7.7 (6.7, 8.7)	8.3 (7.2, 9.6)	2.1 (1.6, 2.6) *	0.6 (0.5,1.7) *
High LDL	49.4 (46.5, 52.3)	47.3 (44.2, 50.3)	48.6 (45.5, 51.7)	0.0 (-0.1, 0.1)	0.4 (0.4,0.5) *

AAPC- average annual percentage change, SEV- summary exposure value, * indicates AAPC is significantly different from zero at the alpha = 0.05 level

For peer review only

Supplementary Table 2. Numbers, age standardised rates and proportions of metabolic risk related NCDs death and DALYs by sex in Australia between 1990 and 2019.

	Number (95% UI)				Age standardised Proportion, % (95%UI)				Age standardised rate per 100,000 (95% UI)			
	1990	2010	2019	% Change 1990-2019	1990	2010	2019	% Change 1990-2019	1990	2010	2019	% Change 1990-2019
**Deaths**												
Male	23052 (21126, 24865)	21726 (19333, 24152)	26,399 (23330, 29621)	13.5 (10.4, 9.1)	41.6 (37.9, 5.1)	33.6 (30.0, 37.3)	32.5 (28.8, 36.3)	-20.2 (-23.9, -19.6)	302.0 (274.7, 327.6)	150.0 (133.7,166.7)	133.9 (118.6, 149.9)	-51.3 (-56.9, -54.3)
Female	22523 (20061, 24693)	23767 (20558, 26597)	26,970 (23156, 30516)	19.8 (15.43, 23.58)	43.7 (38.9, 48.0)	34.8 (30.2, 39.0)	32.8 (28.2, 37.2)	-25.0 (-27.5, -22.5)	199.8 (177.6, 219.0)	107.2 (93.2, 120.2)	94.7 (81.6, 107.4)	52.6 (-54.1, -51.0)
Both	45575 (41530, 49465)	45492 (40228, 50512)	53369 (46725, 59687)	17.1 (12.51, 20.67)	43.0 (38.8, 46.8)	34.4 (30.5, 38.2)	32.8 (28.7, 36.6)	-23.8 (-26.0, -21.8)	245.3 (221.7, 266.8)	127.3 (112.6, 141.1)	113.3 (99.5, 126.6)	-53.8 (-55.1, -52.6)
**DALYs**												
Male	524488 (478223, 573378)	482011 (421511, 548065)	571369 (497917, 659110)	8.9 (4.1, 15.0)	26.6 (23.8, 29.6)	19.4 (17.0, 22.1)	18.9 (16.4, 21.5)	-30.0 (-31.0, -27.5)	6124.6 (5580.6, 6704.6)	3,321.8 (2903.8, 3787.0)	3095.8 (2692.8, 3578.5)	-49.5 (-51.8, -46.6)

For peer review only

Female	411233 (366423, 458999)	425811 (362905, 493866)	489350 (411090, 574086)	19.0 (12.2, 25.1)	19.6 (16.8, 22.6)	14.6 (12.28, 16.9)	14.0 (11.8, 16.4)	-28.8 (-29.7, -27.3)	3,764.7 (3346.1, 4212.5)	2,351.2 (1973.1, 2770.7)	2198.7 (1827.9, 2623.3)	-41.6 (-45.4, -37.7)
Both	935,721 (846039, 1031253)	907822 (786590, 1040661)	1,060719 (911391, 1228621)	13.4 (7.7, 19.1)	23.4 (20.6, 26.4)	17.0 (14.7, 19.6)	16.4 (14.15, 19.0)	-29.7 (-31.1, -28.1)	4,870.9 (4402.1, 5377.5)	2818.2 (2439.8, 3246.5)	2,630.3 (2,246.3, 3063.7)	-46.0 (-49.0, -43.0)

Supplementary Table 3. Burden of non-communicable diseases (deaths and DALYs) attributed to metabolic risk factors in Australia, 2019

Risk factors	Deaths (95%UI)			DALYS (95%UI)		
	Number	Proportion, %(95%UI)	ASR (per 100,000)	Number	Proportion, % (95%UI)	ASR (per 100,000)
Metabolic risk factors combined	53369 (46725,59687)	34.4(30.1, 38.5	113.33(99.49- 126.60)	1060720(911391,1228621)	20.0(17.3, 22.8)	2630.33(246.30,3063.65)
HBP	25498 (20153,30851)	16.4 (12.9, 19.8)	53.8 (43.4, 64.4)	406445 (342906, 471339)	7.7 (6.3, 9.1)	966.5 (824.7, 1111.5)
BMI	18713 (11915,26149)	12.0 (7.7, 16.8)	41.7 (27.0, 57.5)	525135 (353676, 706414)	9.9 (6.8, 12.9)	1387.7 (954.66, 1855.0)
FPG	17640 (12073,25501)	11.4 (7.8, 16.4)	37.2 (25.7, 53.1)	381681 (286916, 499517)	7.2 (5.7, 9.1)	919.9 (698.2, 1187.7)
High LDL	14013 (9490,19216)	9.0 (6.1, 12.4)	30.0 (21.1, 40.10)	212393 (163123, 265910)	4.0 (3.0, 5.1)	525.2 (420.9, 639.0)

ASR- age standardised rate; DALY- disability adjusted life years; UI – uncertainty intervals

For peer review only

Supplementary Table 4. Crude numbers, proportions of specific NCD deaths and DALYs attributable to metabolic risks in 1990 and 2019

Specific Causes of NCDs	Deaths attributable to combined metabolic risks n (95% UI)		DALYs attributable to combined metabolic risks n (95% UI)	
	1990	2019	1990	2019
All NCDs	45575 (49465, 41530)	53369 (59688, 46725)	935721 (846039, 1031253)	1060720 (911391, 1228621)
Neoplasms	2220 (1184, 3500)	5644 (3273, 8658)	47916 (26339, 73613)	110091 (65433, 164880)
Cardiovascular diseases	38412 (34749, 41552)	34921 (29085, 39517)	711313 (657997, 759437)	537319 (475508, 595062)
Chronic respiratory diseases	216 (122, 337)	160 (98, 236)	18915 (30673, 10535)	24758 (15038, 38755)
Neurological disorders	729 (118, 2228)	2954 (529, 8488)	11625 (3291, 29053)	41079 (12673, 98146)
Diabetes mellitus	2198 (2060, 2304.)	4239 (3726, 4629)	78885 (66264, 93765)	186528 (142543, 236662)
Chronic kidney disease	1712 (1557, 1827)	5207 (4293, 5893)	36869 (33602, 40498)	86550 (76642, 96199)
	Proportion % (95% UI)		Proportion % (95% UI)	
All NCDs	42.9 (39.0, 46.5)	34.4 (30.1, 38.5)	24.4 (2.3, 27.5)	20.0 (17.4, 22.8)
Neoplasms	7.1 (3.8, 11.0)	10.5 (6.1, 16.0)	6.4 (3.5, 9.8)	10.0 (5.9, 14.9)
Cardiovascular diseases	75.0 (69.5, 80.2)	67.7 (60.7, 73.7)	75.3 (70.7, 79.6)	68.3 (62.8, 73.3)
Chronic respiratory diseases	3.1 (1.8, 4.8)	1.4 (0.9, 2.1)	7.6 (4.4, 11.6)	7.5 (4.8, 11.1)
Neurological disorders	12.4 (4.0, 24.3)	17.5 (6.5, 30.4)	5.3 (1.8, 12.0)	9.8 (3.8, 19.4)
Diabetes mellitus	100.0 (100.0, 100.0)	100 (100.0,100.0)	100 (100.0, 100.0)	100 (100.0,100.0)
Chronic kidney disease	100 (100.0,100.0)	100 (100.0,100.0)	100 (100.0,100.0)	100 (100.0,100.0)

DALY- disability adjusted life years; NCDs – Non-communicable diseases; UI- uncertainty intervals

For peer review only

Supplementary Table 5. Trends in metabolic risk-related death rates of specific NCDs (age standardised rates) in Australia, 1990 – 2019

Specific Causes of NCDs	Age-standardized death rate (95% UI) per 100,000)			AAPC (95% CI)	
	1990	2010	2019	1990-2019	2010-2019
**Metabolic risks combined**					
Neoplasms	11.4 (6.1 18.0)	13.1 (7.6 , 20.0)	13.0 (7.5, 19.9)	0.5 (0.4, 0.6) *	0.0 (-0.0, 0.1)
Cardiovascular diseases	206.8 (185.1, 224.5)	86.2 (74.6, 96.2)	73.6 (62.2, 82.8)	-3.5 (-3.7, -3.3) *	-1.9 (-2.2, -1.5) *
Chronic respiratory diseases	1.1 (0.6, 1.8)	0.4 (0.3, 0.6)	0.4 (0.2, 0.6)	-3.7 (-4.3, -3.1) *	-1.0 (-1.2, -0.8) *
Neurological disorders	4.5 (0.7, 13.3)	5.8 (1.0, 16.7)	5.8 (1.0, 16.6)	1.0 (0.9, 1.0) *	-0.0 (-0.1, 0.0)
Diabetes mellitus	11.6 (10.7, 12.1)	11.1 (9.9, 11.8)	9.2 (8.2, 10.0)	-0.8 (-1.0, -0.5) *	-2.1 (-2.7, -1.4) *
Chronic kidney disease	9.5 (8.6, 10.1)	10.1 (8.7, 11.0)	10.8 (9.03, 12.1)	0.5 (0.2, 0.9) *	0.7 (0.5, 0.9) *
**High body mass index**					
Neoplasms	7.5 (4.2, 11.4)	8.3 (5.1, 11.8)	8.5 (5.2, 12.0)	0.4 (0.3, 0.5) *	0.1 (0.1, 0.2) *
Cardiovascular diseases	45.6 (26.2, 68.1)	22.5 (14.1, 32.0)	20.1 (12.7, 28.4)	-2.8 (-3.0, -2.6) *	-1.4 (-1.7, -1.1) *
Chronic respiratory diseases	2.8 (1.4, 4.5)	3.5 (1.6, 5.5)	0.4 (0.2, 0.6)	-3.7 (-4.3, -3.1) *	-1.0 (-1.2, -0.8) *

For peer review only

Neurological disorders	3.1 (0.5, 9.5)	4.0 (0.7, 11.8)	4.1 (0.7, 12.2)	1.1 (1.0, 1.0) *	0.3 (0.2, 0.5) *
Diabetes mellitus	4.8 (3.1, 6.6)	5.1 (3.4, 6.9)	4.3 (2.9, 5.9)	-0.4 (-0.6, 0.1) *	-2.0 (-2.6, -1.4) *
Chronic kidney disease	2.8 (1.4, 4.5)	3.5 (1.6, 5.5)	3.8 (1.8, 6.0)	1.1 (0.8, 1.4) *	0.9 (0.5, 1.4) *
**High fast plasma glucose**					
Neoplasms	4.1 (1.1, 8.5)	5.1 (1.4, 10.5)	4.8 (1.3, 9.9)	0.6 (0.4, 0.8) *	-0.6 (-1.1, -0.0) *
Cardiovascular diseases	32.4 (19.9, 53.5)	26.0 (14.8, 44.2)	20.1 (11.8, 33.4)	-1.5 (-1.8, -1.2) *	-2.5 (-2.9, -2.1) *
Neurological disorders	1.6 (0.2, 6.3)	2.2 (0.2, 8.4)	2.1 (0.2, 7.8)	0.9 (0.8, 0.9) *	-0.8 (-1.0, -0.7) *
Diabetes mellitus	11.6 (10.7, 12.1)	11.1 (9.9, 11.8)	9.2 (8.2, 10.0)	-0.8 (-1.0, -0.5) *	-2.1 (-2.7, -1.4) *
Chronic kidney disease	0.6 (0.4, 0.8)	1.0 (0.9, 1.2)	1.0 (0.7, 1.5)	2.0 (1.7, 2.2) *	-0.4 (-0.6, -0.1) *
**High Systolic blood pressure**					
Cardiovascular diseases	149.6 (125.5, 170.5	54.7 (44.2, 65.5)	47.3 (37.5, 57.3)	-3.9 (-4.1, -3.7) *	-1.8 (-2.2, -1.5) *
Chronic kidney disease	6.1 (5.2, 6.9)	6.2 (5.2, 7.1)	6.5 (5.3, 7.7)	0.3 (-0.1, 0.6)	0.6 (0.4,0.8)
**High LDL cholesterol**					

For peer review only

Cardiovascular diseases	94.9 (70.4, 122.1	35.5 (24.8, 47.7)	30.0 (21.1, 40.1)	-3.9 (-4.1, -3.7) *	-2.0 (-2.4, -1.6) *

AAPC- Average annual percent change; LDL-low density lipoprotein; NCDs – Non-communicable diseases; UI- uncertainty intervals

*AAPC is significantly different from zero at the $\alpha = 0.05$ levels

For peer review only

Supplementary Table 6. Trends in metabolic risk-attributed DALY rates of specific NCDs (age standardised rates) in Australia

	Age-standardized DALY rates (95% UI) per 100,000)			AAPC (95% CI)	
Specific Causes of NCDs	1990	2010	2019	1990-2019	2010-2019
**Combined metabolic risks**					
Neoplasms	247.4 (136.6, 379.1)	274.8 (162.7, 410.0)	274.2 (164.4, 407.6)	0.4 (0.3, 0.4) *	-0.0 (-0.1, 0.0)
Cardiovascular diseases	3689.4 (3407.5, 3945.1)	1479.7 (1338.2, 1615.6)	1279.8 (1145.9, 1410.1)	-3.6 (-3.8, -3.4) *	-1.7 (-2.0, -1.4) *
Chronic respiratory diseases	104.0 (58.7, 167.7)	83.7 (50.8, 127.7)	88.52 (54.1, 138.7)	-0.6 (-0.8, -0.4) *	0.9 (0.7, 1.1) *
Neurological disorders	65.0 (18.1, 161.8)	84.6 (26.5, 198.8)	85.1 (26.4, 202.8)	1.0 (0.9, 1.0) *	0.1 (0.0, 0.2) *
Diabetes mellitus	409.5 (343.5, 486.2)	484.9 (386.0, 610.1)	480.8 (365.6, 613.0)	0.6 (0.5, 0.6) *	-0.1 (-0.6, 0.5)
Chronic kidney disease	195.4 (178.0, 214.3)	198.5 (177.7, 220.9)	208.5 (184.8, 233.2)	0.3 (0.0, 0.5) *	0.5 (-0.2, 1.3) *
**High body mass index**					
Neoplasms	173.6 (99.7, 260.5)	187.5 (116.6, 264.1)	190.3 (118.8, 265.9)	0.3 (0.2, 0.4) *	0.1 (0.0, 0.2) *
Cardiovascular diseases	1057.9 (633.0, 1511.9)	532.5 (361.1, 716.4)	477.8 (327.8, 641.5)	-2.7 (-3.0, -2.5) *	-1.2 (-1.5, -0.1) *
Chronic respiratory diseases	104.0 (58.7, 167.7)	83.7 (50.8, 127.7)	88.5 (54.1, 138.7)	-0.6 (-0.8, -0.4) *	0.9 (0.7, 1.1) *

For peer review only

Neurological disorders	46.3 (11.4, 120.2)	59.4 (16.6, 144.5)	61.6 (17.4, 152.5)	1.0 (0.9, 1.0) *	0.4 (0.3, 0.5) *
Diabetes mellitus	198.7 (134.2, 270.9)	270.2 (187.7, 366.0)	279.5 (192.1, 383.4)	1.2 (1.0, 1.5) *	0.5 (0.4, 0.5) *
Chronic kidney disease	64.6 (37.0, 95.1)	76.0 (47.0, 106.9)	82.7 (52.6, 116.2)	0.9 (0.7, 1.1) *	0.9 (0.2, 1.6) *
**High fast plasma glucose**					
Neoplasms	76.9 (19.7, 161.3)	92.2 (24.6, 189.4)	89.1 (23.9, 184.0)	0.6 (0.5, 0.7) *	-0.2 (-0.3, 0.2) *
Cardiovascular diseases	471.5 (314.5, 714.9)	353.4 (218.7, 561.1)	289.8 (186.2, 455.0)	-1.5 (-1.8, -1.3) *	-1.9 (-2.3, -1.5) *
Neurological disorders	21.7 (3.5,74.0)	30.9 (5.2, 101.0)	29.1 (5.1, 95.7)	1.0 (0.9, 1.1) *	-0.7 (-0.8, -0.5) *
Diabetes mellitus	409.5 (343.5, 486.2)	484.9 (386.0, 610.1)	480.8 (365.6, 613.0)	0.6 (0.5, 0.6) *	-0.1 (-0.1, 0.0)
Chronic kidney disease	19.9 (15.9, 24.8)	29.3 (24.8, 35.1)	29.1 (22.1, 37.3)	1.3 (1.3, 1.4) *	0.1 (-0.1, 0.2)
**High Systolic blood pressure**					
Cardiovascular diseases	2713.3 (2402.2, 3033.0)	974.9 (839.3, 1112.3)	855.6 (720.6, 989.9)	-3.9 (-4.1, -3.8) *	-1.6 (-1.9, -1.3) *
Chronic kidney disease	109.3 (92.8, 126.6)	104.7 (88.6, 120.8)	110.9 (92.3, 131.4)	0.1 (-0.0, 0.3)	0.7 (0.6, 0.9) *
**High LDL cholesterol**					
Cardiovascular diseases	1757.9 (1443.6, 2100.7)	623.8 (504.1, 761.0)	525.2 (420.9, 639.0)	-4.1 (-4.4, -3.8) *	-2.0 (-2.3, -1.7) *

AAPC- Average annual percent change; LDL-low density lipoprotein; NCDs – Non-communicable diseases; UI- uncertainty intervals; *AAPC is significantly different from zero at the alpha = 0.05 levels

For peer review only

Supplementary Figures

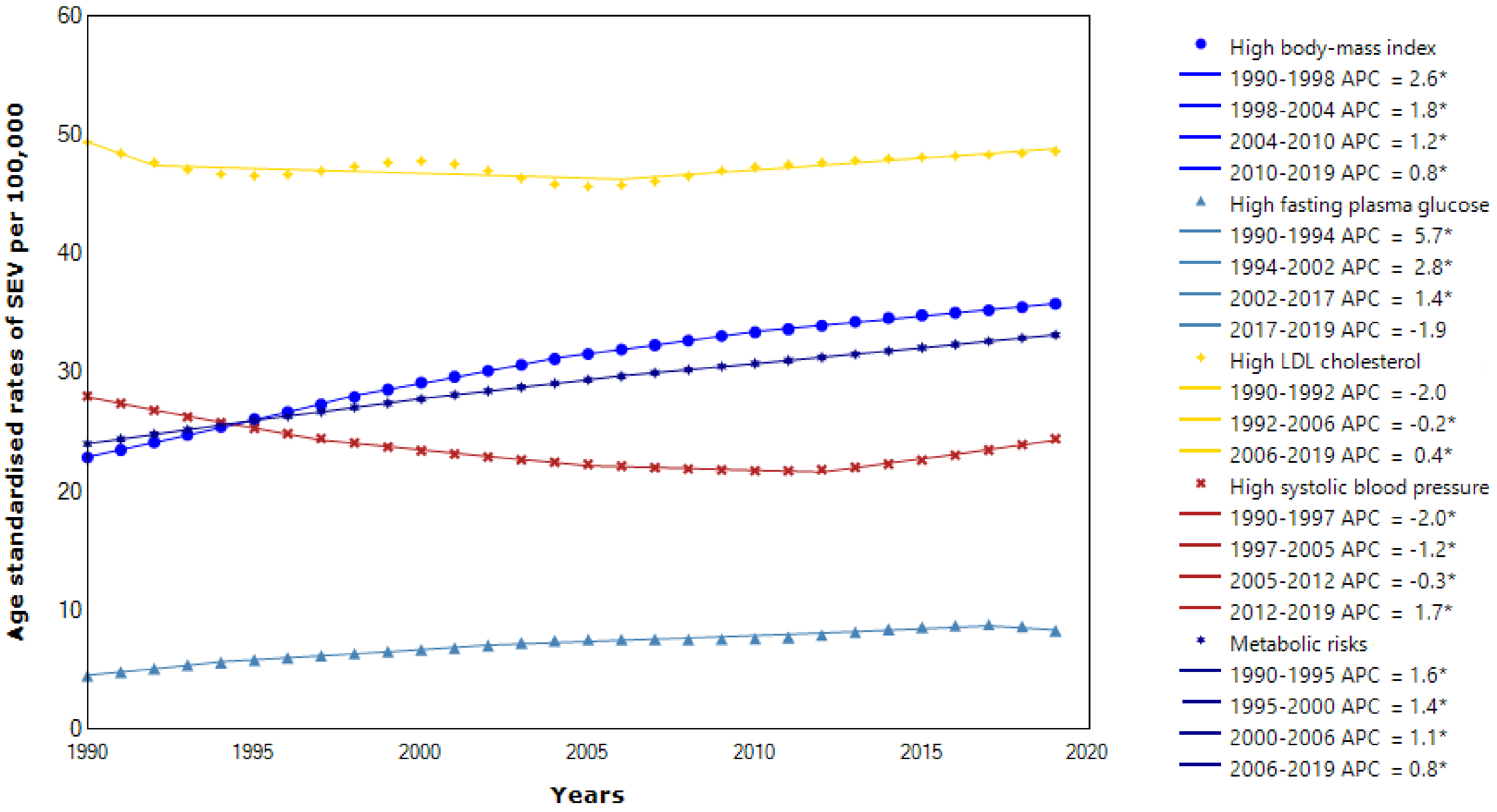

Supplementary Figure 1. Trends of SEV of combined and individual metabolic risks factors between 1990 and 2019, in Australia. Note: * indicates APC is significantly different from zero at the $\alpha = 0.05$ level

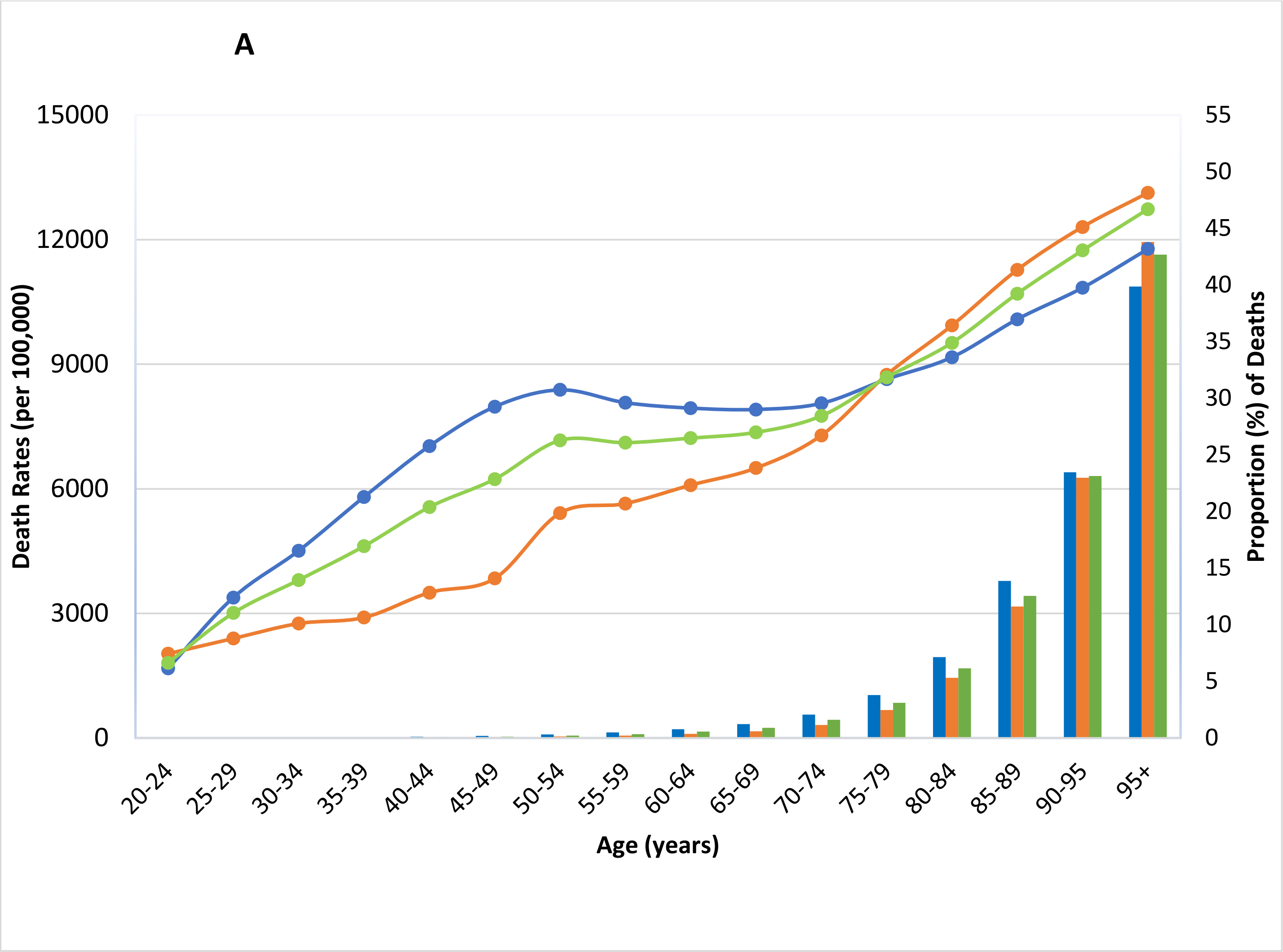
A
Death Rates (per 100,000)
15000
12000
9000
6000
3000
0
Proportion (%) of Deaths
55
50
45
40
35
30
25
20
15
10
5
0
20-24
25-29
30-34
35-39
40-44
45-49
50-54
55-59
60-64
65-69
70-74
75-79
80-84
85-89
90-95
95+
Age (years)

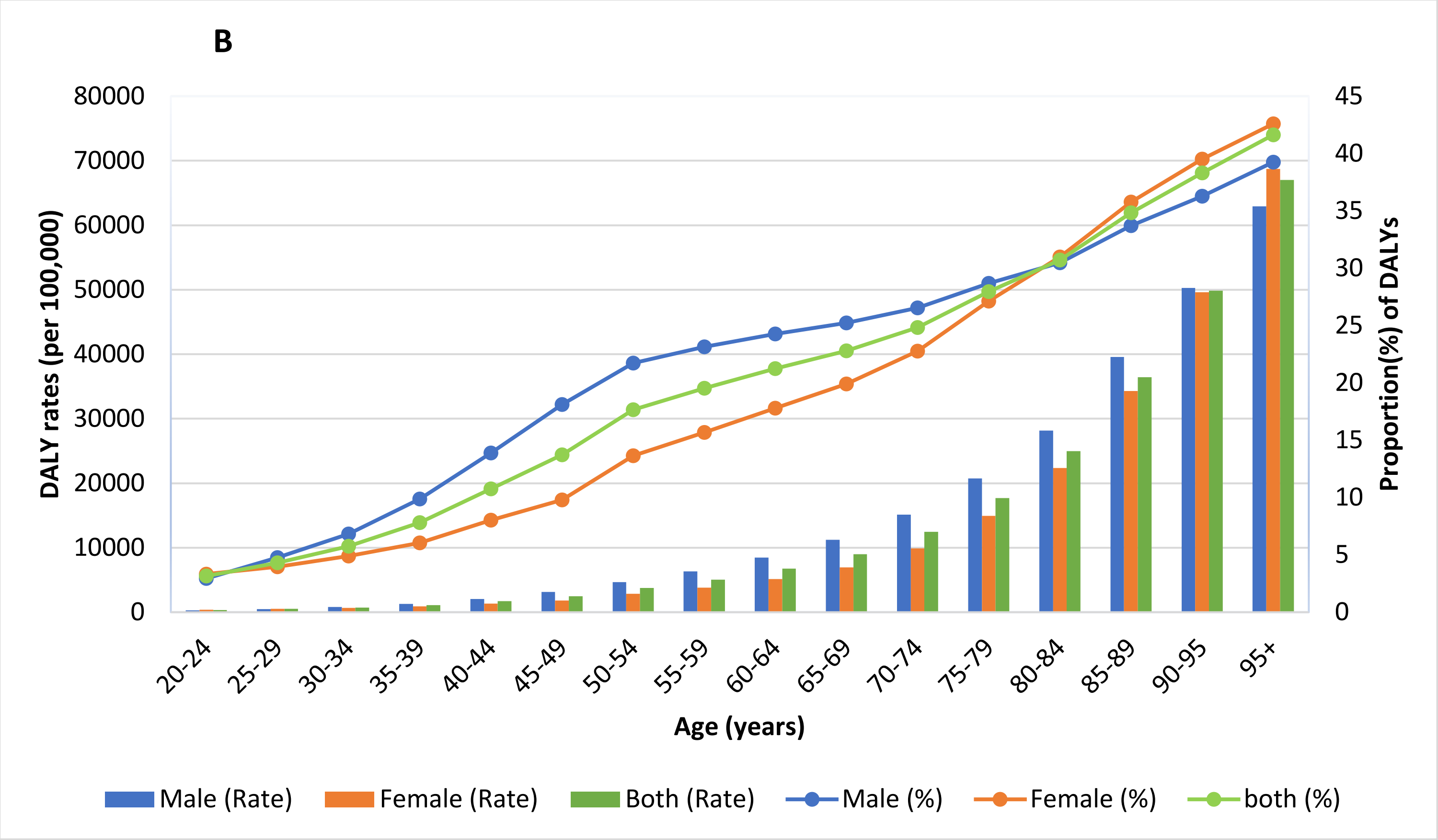

Supplementary Figure 2. Age-specific rates and proportions of non-communicable disease deaths (**A**), and DALYs (**B**) attributable to metabolic risk factors by sex in Australia in 2019 (Proportions are of all NCD deaths/DALYs).

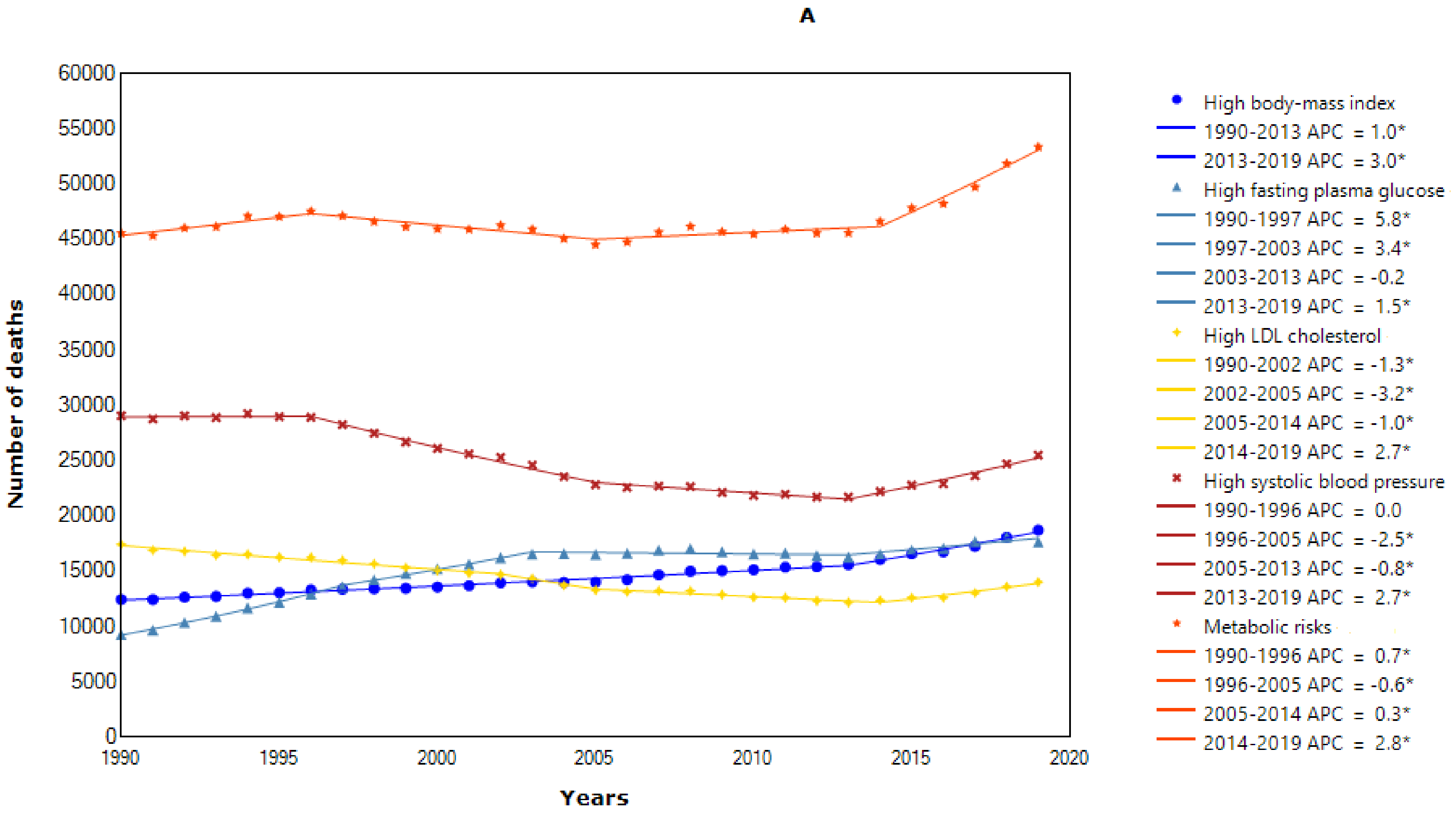
A
Number of deaths
60000
55000
50000
45000
40000
35000
30000
25000
20000
15000
10000
5000
0
1990
1995
2000
2005
2010
2015
2020
Years
High body-mass index
1990-2013 APC = 1.0*
2013-2019 APC = 3.0*
High fasting plasma glucose
1990-1997 APC = 5.8*
1997-2003 APC = 3.4*
2003-2013 APC = -0.2
2013-2019 APC = 1.5*
High LDL cholesterol
1990-2002 APC = -1.3*
2002-2005 APC = -3.2*
2005-2014 APC = -1.0*
2014-2019 APC = 2.7*
High systolic blood pressure
1990-1996 APC = 0.0
1996-2005 APC = -2.5*
2005-2013 APC = -0.8*
2013-2019 APC = 2.7*
Metabolic risks
1990-1996 APC = 0.7*
1996-2005 APC = -0.6*
2005-2014 APC = 0.3*
2014-2019 APC = 2.8*

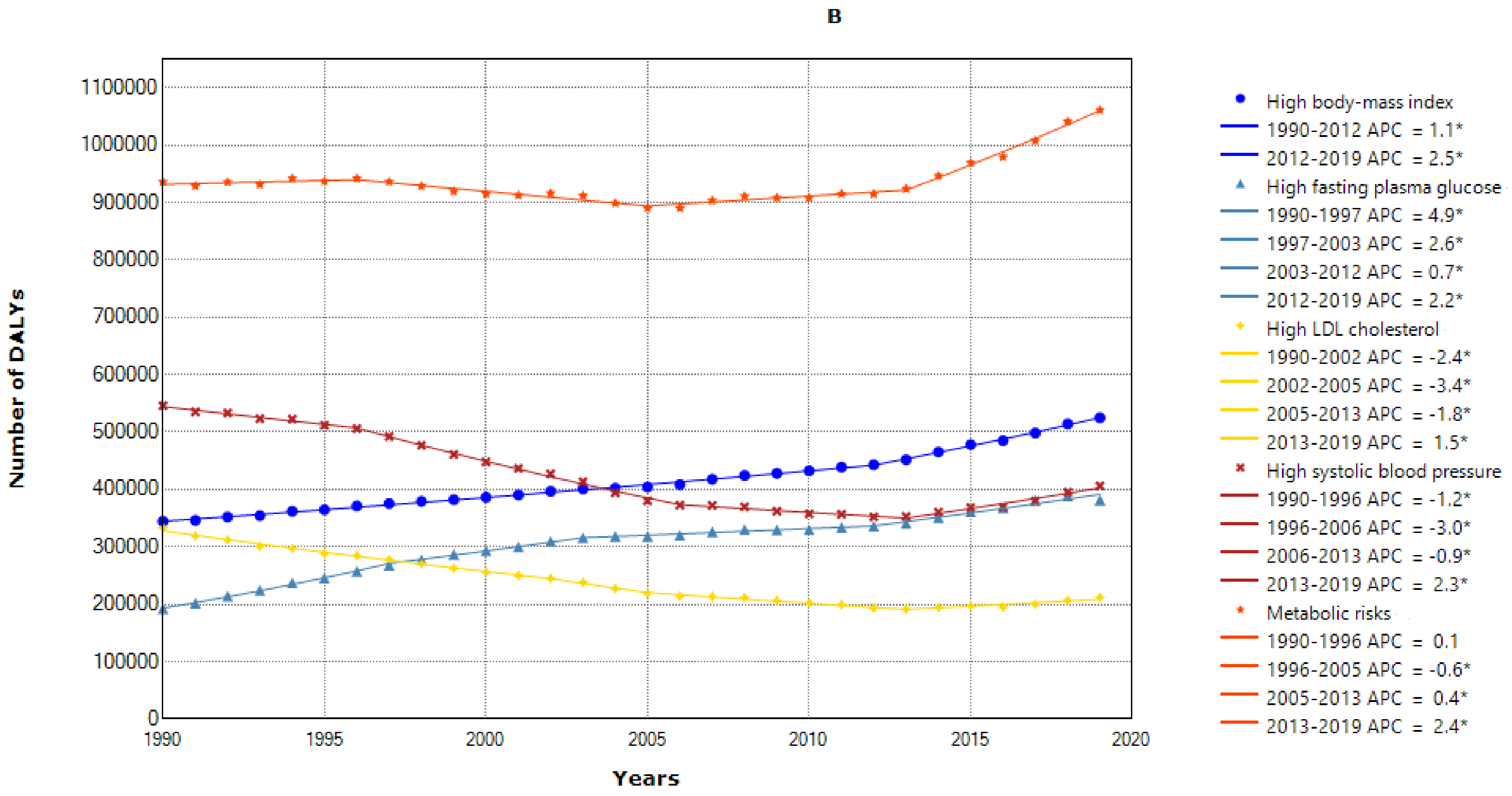

Supplementary Figures 3. Number of deaths (**A**) and DALYs (**B**) attributed to individual and combined metabolic risks overt time (1990-2019) in Australia from Join point regression analysis (Note: * indicates APC is significantly different from zero at the α = 0.05 levels).

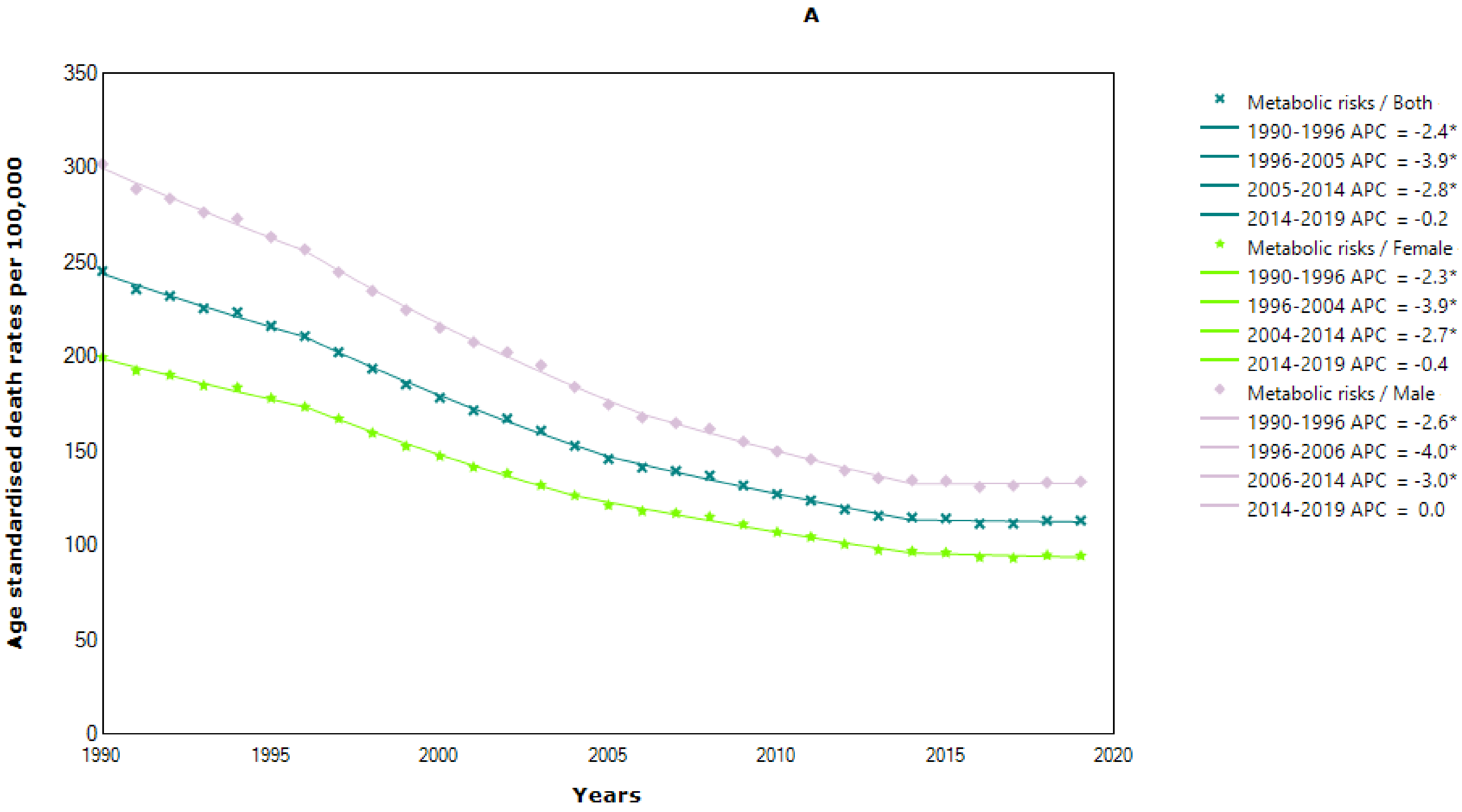
A
Age standardised death rates per 100,000
350
300
250
200
150
100
50
0
1990
1995
2000
2005
2010
2015
2020
Years
Metabolic risks / Both
1990-1996 APC = -2.4*
1996-2005 APC = -3.9*
2005-2014 APC = -2.8*
2014-2019 APC = -0.2
Metabolic risks / Female
1990-1996 APC = -2.3*
1996-2004 APC = -3.9*
2004-2014 APC = -2.7*
2014-2019 APC = -0.4
Metabolic risks / Male
1990-1996 APC = -2.6*
1996-2006 APC = -4.0*
2006-2014 APC = -3.0*
2014-2019 APC = 0.0

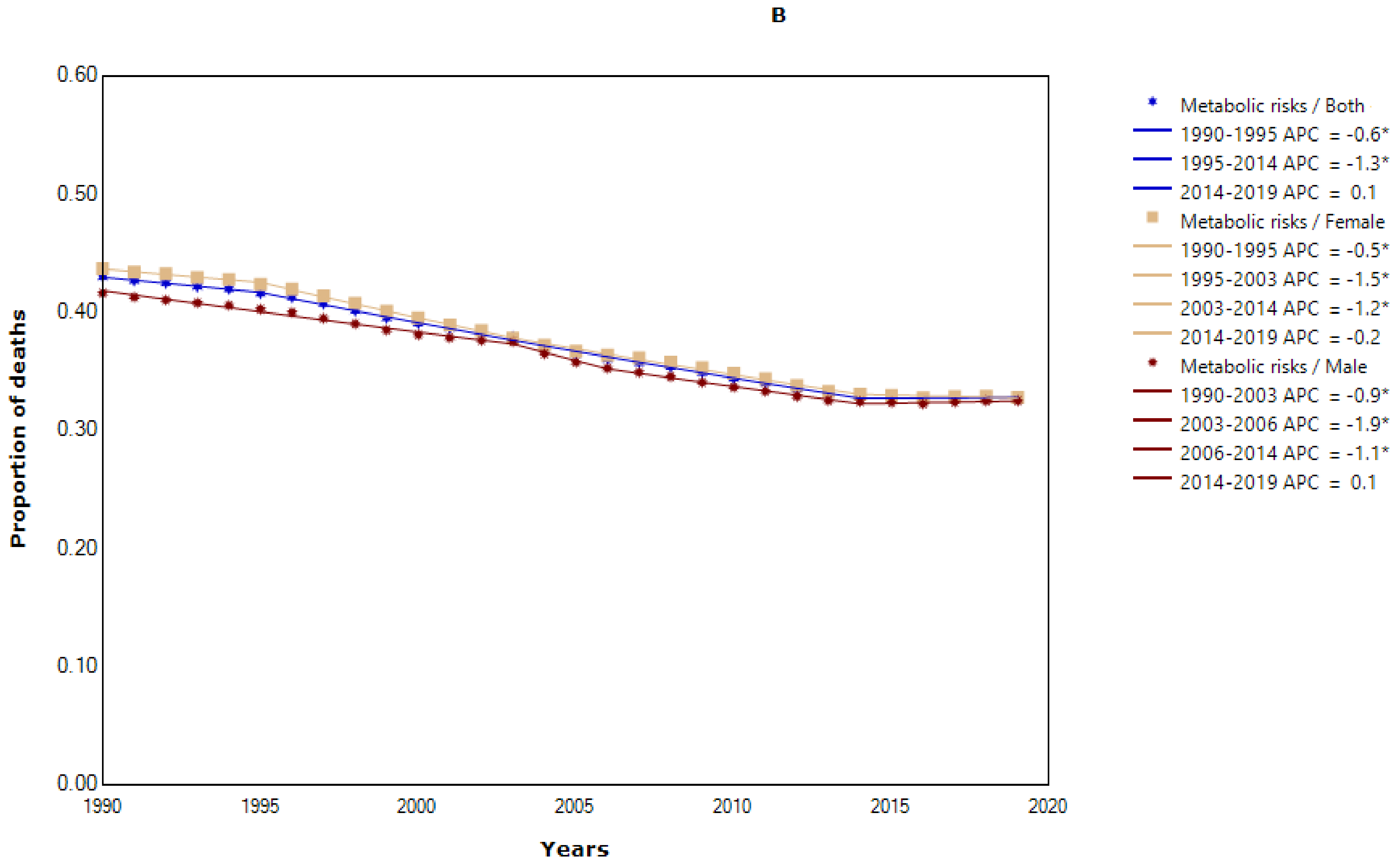
B
0.60
0.50
0.40
0.30
0.20
0.10
0.00
1990
1995
2000
2005
2010
2015
2020
Proportion of deaths
Years
Metabolic risks / Both
1990-1995 APC = -0.6*
1995-2014 APC = -1.3*
2014-2019 APC = 0.1
Metabolic risks / Female
1990-1995 APC = -0.5*
1995-2003 APC = -1.5*
2003-2014 APC = -1.2*
2014-2019 APC = -0.2
Metabolic risks / Male
1990-2003 APC = -0.9*
2003-2006 APC = -1.9*
2006-2014 APC = -1.1*
2014-2019 APC = 0.1

C

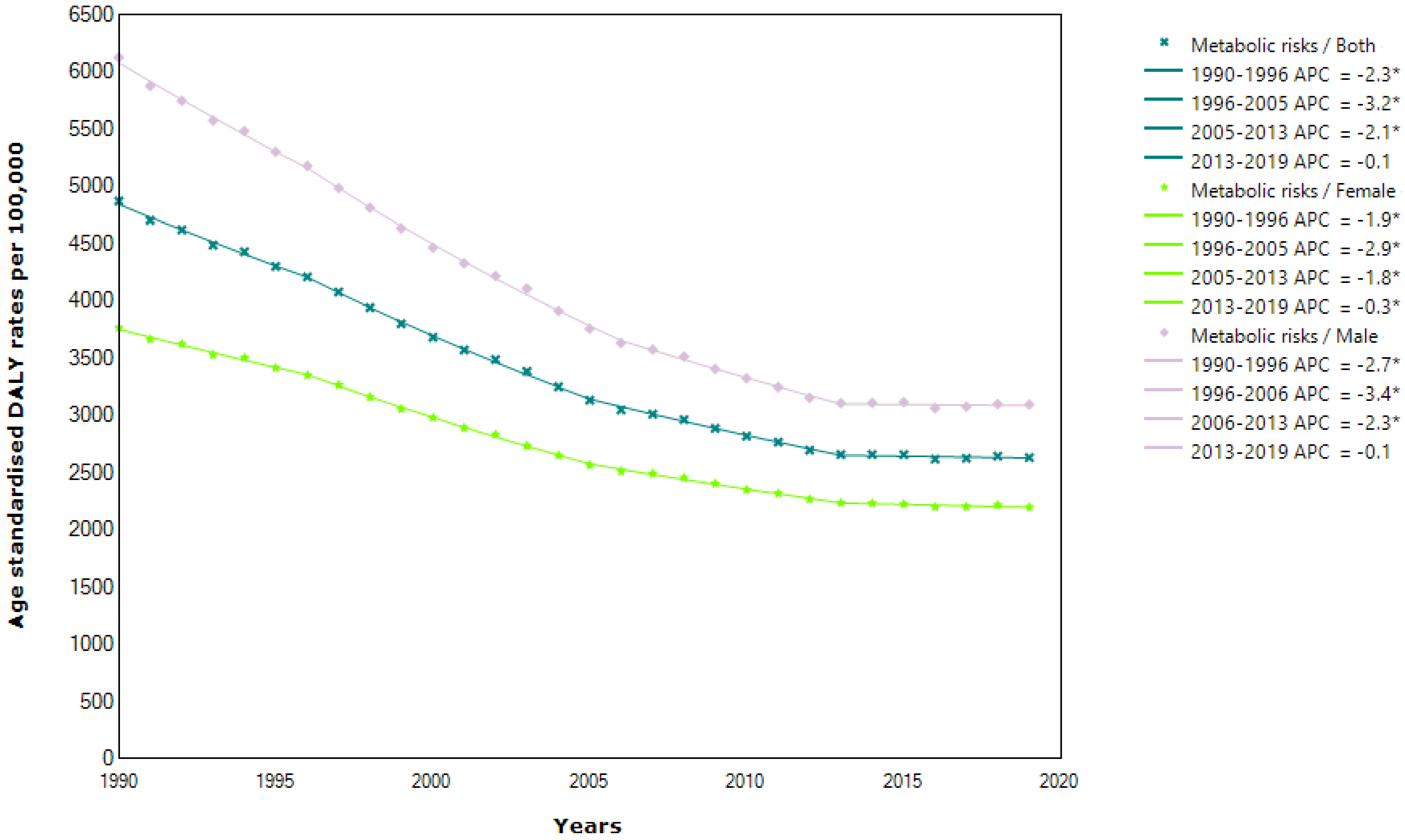
6500
6000
5500
5000
4500
4000
3500
3000
2500
2000
1500
1000
500
0
Age standardised DALY rates per 100,000
1990
1995
2000
2005
2010
2015
2020
Years
Metabolic risks / Both
1990-1996 APC = -2.3*
1996-2005 APC = -3.2*
2005-2013 APC = -2.1*
2013-2019 APC = -0.1
Metabolic risks / Female
1990-1996 APC = -1.9*
1996-2005 APC = -2.9*
2005-2013 APC = -1.8*
2013-2019 APC = -0.3*
Metabolic risks / Male
1990-1996 APC = -2.7*
1996-2006 APC = -3.4*
2006-2013 APC = -2.3*
2013-2019 APC = -0.1

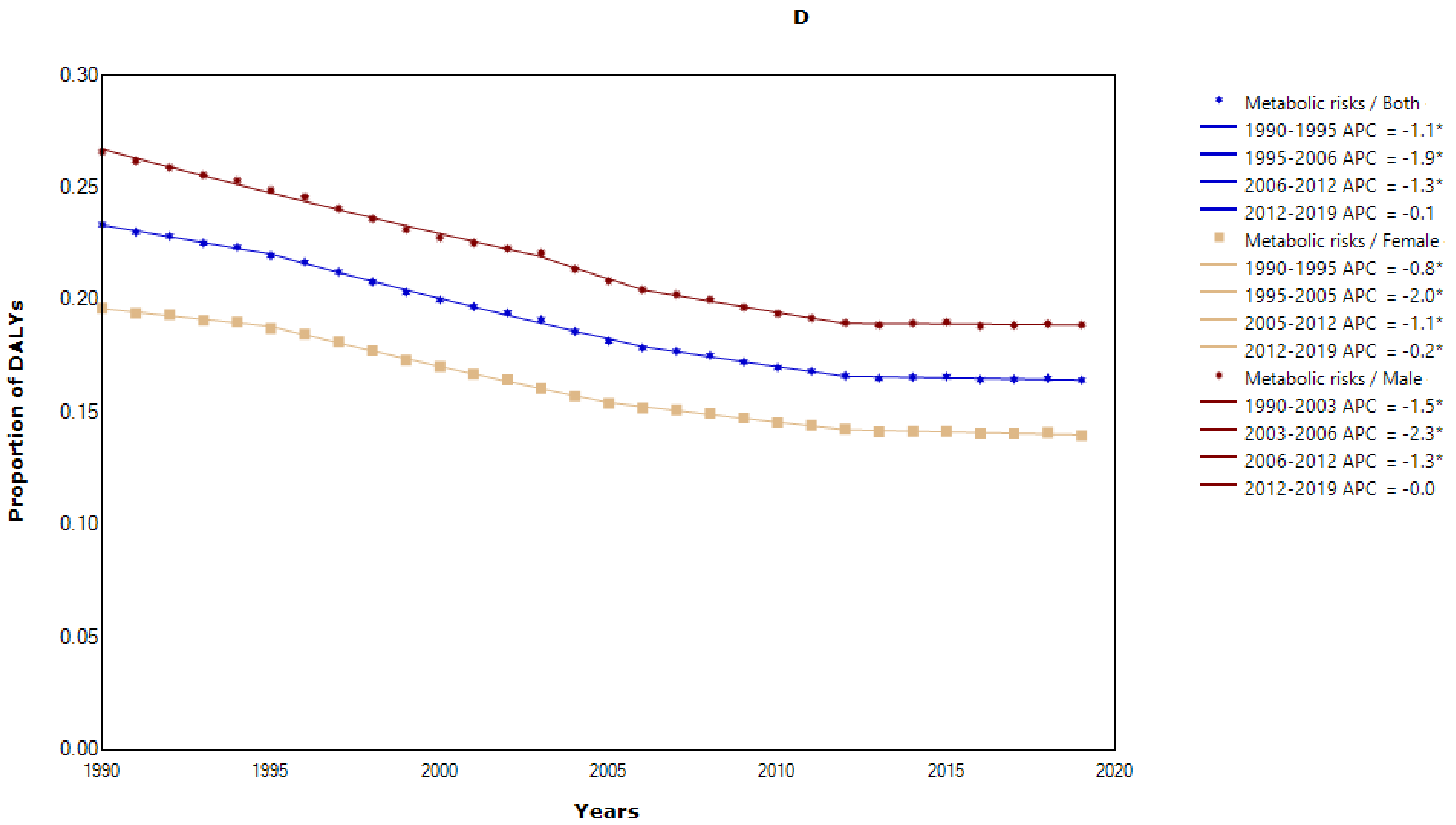

Supplementary Figure 4. Trends of NCDs deaths and DALYs attributable to combined metabolic risks over time (1990 to 2019) by sex in Australia from Join point regression analysis: Age-standardized death rates (**A**); proportions of deaths **(B)**; Age standardised DALY rates **(C)** proportion of DALYs (**D**) (Note: * indicates APC is significantly different from zero at the α = 0.05 levels)

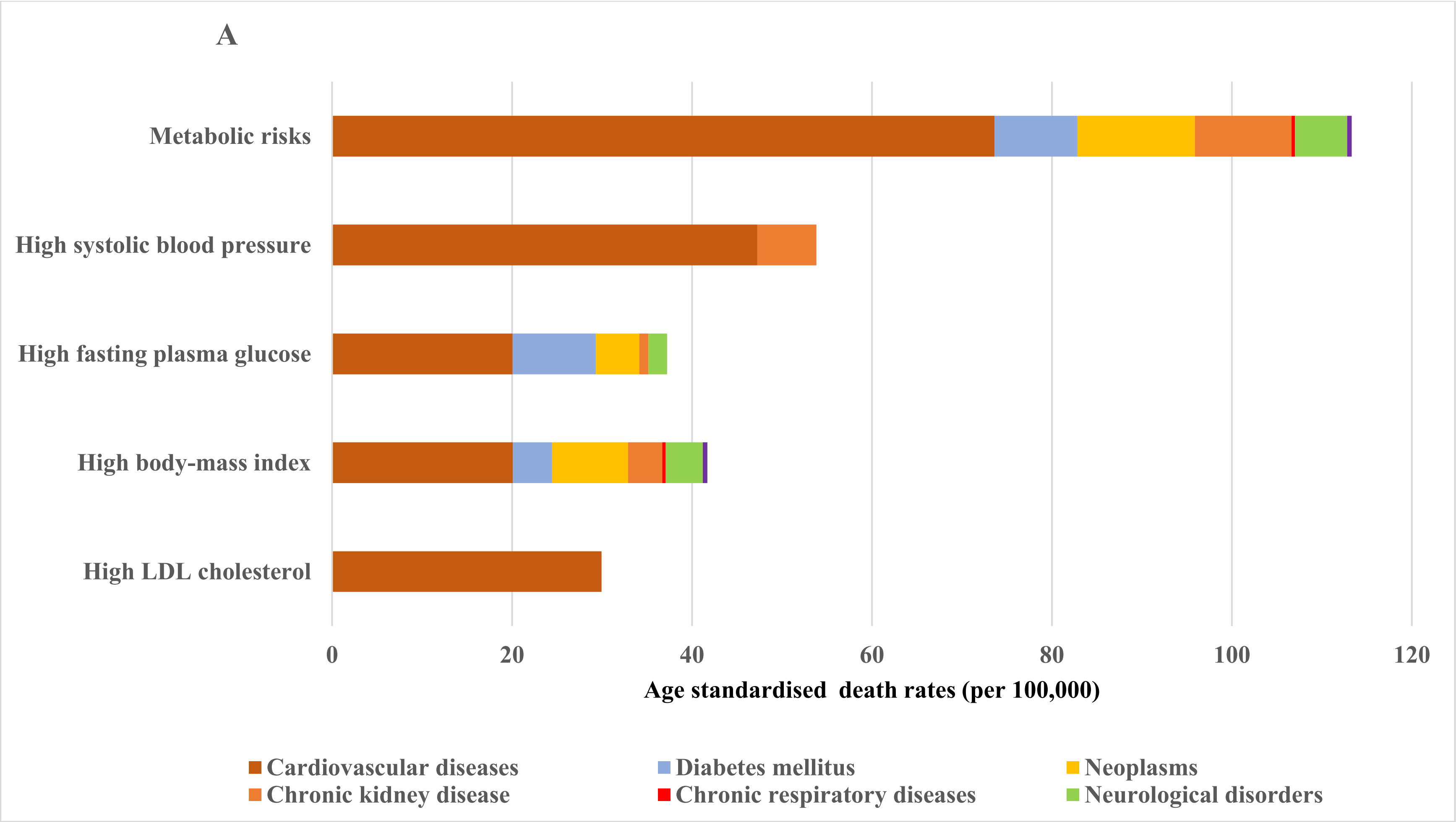
A
Metabolic risks
High systolic blood pressure
High fasting plasma glucose
High body-mass index
High LDL cholesterol
0
20
40
60
80
100
120
Age standardised death rates (per 100,000)
Cardiovascular diseases
Diabetes mellitus
Neoplasms
Chronic kidney disease
Chronic respiratory diseases
Neurological disorders

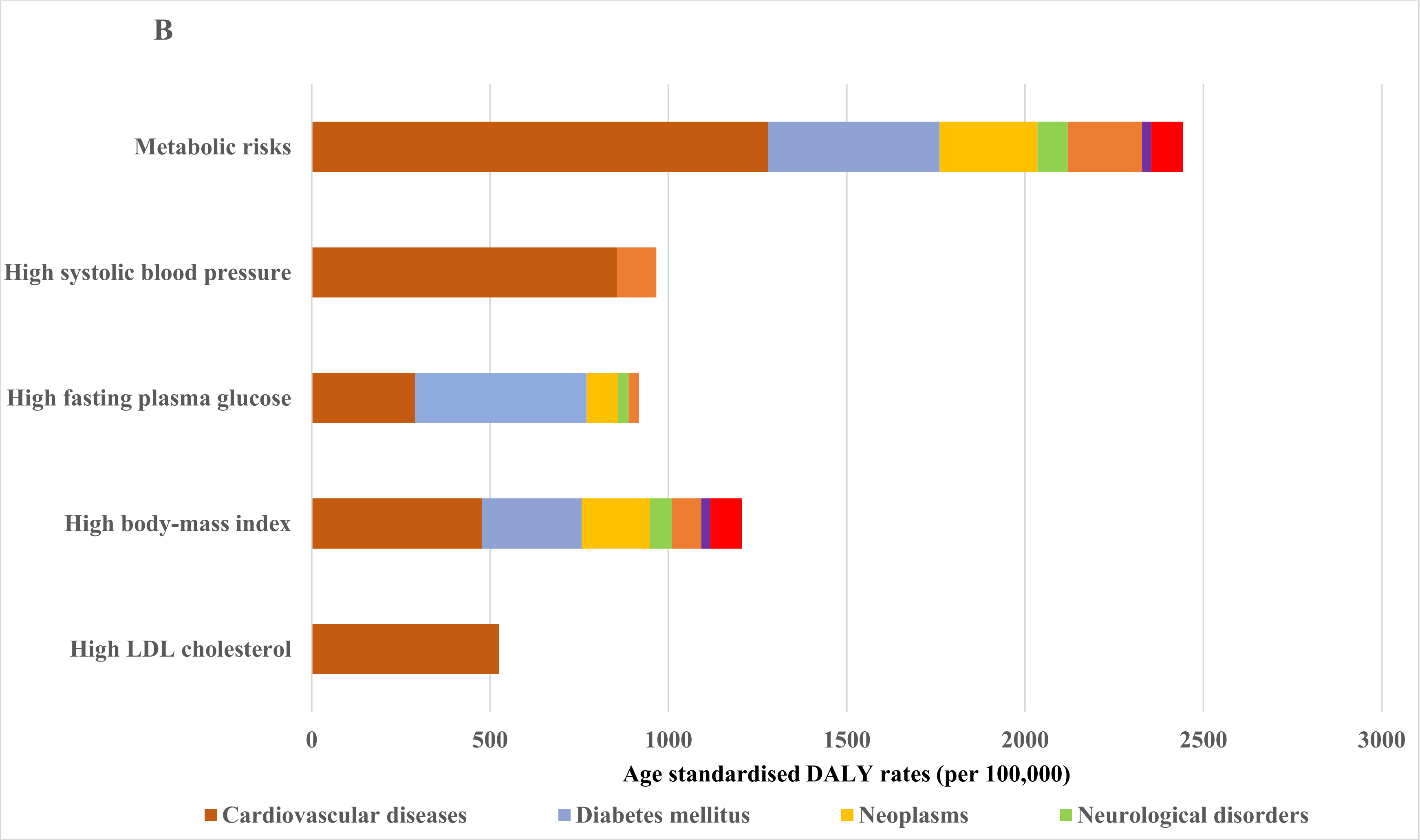

Supplementary Figure 5. Burden of specific NCDs attributable to individual and combined metabolic risks: age standardised death rates (A) and DALY rates (B) in Australia, 2019

STROBE 2007 (v4) checklist of items to be included in reports of observational studies in epidemiology*

Checklist for cohort, case-control, and cross-sectional studies (combined)

**Section/Topic**	**Item #**	**Recommendation**	**Reported on page #**
**Title and abstract**	1	(*a*) Indicate the study's design with a commonly used term in the title or the abstract	1,2
		(*b*) Provide in the abstract an informative and balanced summary of what was done and what was found	2
**Introduction**			
Background/rationale	2	Explain the scientific background and rationale for the investigation being reported	3-4
Objectives	3	State specific objectives, including any pre-specified hypotheses	4
**Methods**			
Study design	4	Present key elements of study design early in the paper	5
Setting	5	Describe the setting, locations, and relevant dates, including periods of recruitment, exposure, follow-up, and data collection	5
Participants	6	(*a*) *Cohort study*—Give the eligibility criteria, and the sources and methods of selection of participants. Describe methods of follow-up<br>*Case-control study*—Give the eligibility criteria, and the sources and methods of case ascertainment and control selection. Give the rationale for the choice of cases and controls<br>*Cross-sectional study*—Give the eligibility criteria, and the sources and methods of selection of participants	5
		(*b*) *Cohort study*—For matched studies, give matching criteria and number of exposed and unexposed<br>*Case-control study*—For matched studies, give matching criteria and the number of controls per case	
Variables	7	Clearly define all outcomes, exposures, predictors, potential confounders, and effect modifiers. Give diagnostic criteria, if applicable	5-6
Data sources/ measurement	8*	For each variable of interest, give sources of data and details of methods of assessment (measurement). Describe comparability of assessment methods if there is more than one group	*6*
Bias	9	Describe any efforts to address potential sources of bias	6
Study size	10	Explain how the study size was arrived at	7
Quantitative variables	11	Explain how quantitative variables were handled in the analyses. If applicable, describe which groupings were chosen and why	7
Statistical methods	12	(*a*) Describe all statistical methods, including those used to control for confounding	7-8
		(*b*) Describe any methods used to examine subgroups and interactions	
		(*c*) Explain how missing data were addressed	
		(*d*) *Cohort study*—If applicable, explain how loss to follow-up was addressed<br>*Case-control study*—If applicable, explain how matching of cases and controls was addressed	

For peer review only

		Cross-sectional study—If applicable, describe analytical methods taking account of sampling strategy	
		(*e*) Describe any sensitivity analyses	
**Results**			
Participants	13*	(a) Report numbers of individuals at each stage of study—eg numbers potentially eligible, examined for eligibility, confirmed eligible, included in the study, completing follow-up, and analysed	8
		(b) Give reasons for non-participation at each stage	
		(c) Consider use of a flow diagram	
Descriptive data	14*	(a) Give characteristics of study participants (eg demographic, clinical, social) and information on exposures and potential confounders	8,9
		(b) Indicate number of participants with missing data for each variable of interest	
		(c) *Cohort study*—Summarise follow-up time (eg, average and total amount)	
Outcome data	15*	*Cohort study*—Report numbers of outcome events or summary measures over time	9
		Case-control study—Report numbers in each exposure category, or summary measures of exposure	
		Cross-sectional study—Report numbers of outcome events or summary measures	
Main results	16	(*a*) Give unadjusted estimates and, if applicable, confounder-adjusted estimates and their precision (eg, 95% confidence interval). Make clear which confounders were adjusted for and why they were included	9-16
		(*b*) Report category boundaries when continuous variables were categorized	
		(*c*) If relevant, consider translating estimates of relative risk into absolute risk for a meaningful time period	
Other analyses	17	Report other analyses done—eg analyses of subgroups and interactions, and sensitivity analyses	
**Discussion**			
Key results	18	Summarise key results with reference to study objectives	16
Limitations	19	Discuss limitations of the study, taking into account sources of potential bias or imprecision. Discuss both direction and magnitude of any potential bias	19, 20
Interpretation	20	Give a cautious overall interpretation of results considering objectives, limitations, multiplicity of analyses, results from similar studies, and other relevant evidence	16-20
Generalisability	21	Discuss the generalisability (external validity) of the study results	20
**Other information**			
Funding	22	Give the source of funding and the role of the funders for the present study and, if applicable, for the original study on which the present article is based	20,21

For peer review only
